# Non-coding variants impact *cis*-regulatory coordination in a cell type-specific manner

**DOI:** 10.1186/s13059-024-03333-4

**Published:** 2024-07-18

**Authors:** Olga Pushkarev, Guido van Mierlo, Judith Franziska Kribelbauer, Wouter Saelens, Vincent Gardeux, Bart Deplancke

**Affiliations:** 1 Laboratory of Systems Biology and Genetics, Institute of Bioengineering, School of Life Sciences, École Polytechnique Fédérale de Lausanne (EPFL), Lausanne, Switzerland; 2 Swiss Institute of Bioinformatics (SIB), Lausanne, Switzerland

**Keywords:** *Cis*-regulatory interactions, Epigenomics, Genome-wide association studies, Quantitative trait loci, Gene regulation

## Abstract

**Background:**

Interactions among *cis*-regulatory elements (CREs) play a crucial role in gene regulation. Various approaches have been developed to map these interactions genome-wide, including those relying on interindividual epigenomic variation to identify groups of covariable regulatory elements, referred to as chromatin modules (CMs). While CM mapping allows to investigate the relationship between chromatin modularity and gene expression, the computational principles used for CM identification vary in their application and outcomes.

**Results:**

We comprehensively evaluate and streamline existing CM mapping tools and present guidelines for optimal utilization of epigenome data from a diverse population of individuals to assess regulatory coordination across the human genome. We showcase the effectiveness of our recommended practices by analyzing distinct cell types and demonstrate cell type specificity of CRE interactions in CMs and their relevance for gene expression. Integration of genotype information revealed that many non-coding disease-associated variants affect the activity of CMs in a cell type-specific manner by affecting the binding of cell type-specific transcription factors. We provide example cases that illustrate in detail how CMs can be used to deconstruct GWAS loci, assess variable expression of cell surface receptors in immune cells, and reveal how genetic variation can impact the expression of prognostic markers in chronic lymphocytic leukemia.

**Conclusions:**

Our study presents an optimal strategy for CM mapping and reveals how CMs capture the coordination of CREs and its impact on gene expression. Non-coding genetic variants can disrupt this coordination, and we highlight how this may lead to disease predisposition in a cell type-specific manner.

**Supplementary Information:**

The online version contains supplementary material available at 10.1186/s13059-024-03333-4.

## Background

The genome inside the nucleus is spatially organized with every chromosome occupying its own territory. Each chromosome is coarsely divided into active (A) and inactive (B) compartments [1], and topologically associating domains (TADs) [2, 3]. Within TADs, fine-grained chromatin organization is established through interactions of non-coding, *cis*-regulatory elements (CREs; here defined as enhancers and promoters). Such CRE interactions are critical for gene regulation, as most promoters alone possess low intrinsic power for regulating gene expression [4], and active genes interact on average with 2–4 enhancers [5, 6]. Depending on the context, perturbation of one or multiple enhancers can be sufficient to largely attenuate gene expression through disruption of regulatory CRE interactions [7–9].

CREs can be accurately mapped using epigenome profiling assays such as ATAC-seq and ChIP-seq, and stratified into different categories according to their regulatory properties (enhancer vs promoter, weak vs strong, etc.) on a per cell type basis [10]. Further epigenome-based studies on cohorts of non-related individuals in single cell types revealed that not every CRE harbors equal chromatin accessibility or activity across individuals [11]. Downstream association studies pairing this activity to commonly occurring genetic variants allowed to identify loci (i.e., quantitative trait loci; QTLs) that impact either CRE accessibility due to disruption of transcription factor (TF) binding site(s) or histone modification deposition [12, 13]. Such a “population epigenomics” approach has been used to link genetic variants to the activity of CREs and gene expression in a range of cell types and conditions [14–16]. While highly valuable, these studies mostly focused on individual CREs and thus did not explicitly assess how CREs interact to regulate the expression of nearby genes.

Chromosome conformation capture (3C) as well as microscopy-based approaches allows linking enhancers and promoters into units of functionally collaborating CREs, although this can be challenging in terms of resolution and throughput [17]. An orthogonal approach is to perform epigenome analyses on many different genotypes, enabling the identification of covariable CREs. This allows the grouping of CREs based on their interindividual variability into coordinated, epigenomic hubs within TADs, which are referred to as chromatin modules (CMs [17]). Such an approach allows the mapping of CRE coordination in a genome-wide manner and, as compared to Hi-C and microscopy assays, provides direct epigenomic readouts of CRE activity and therefore allows revealing the impact of genetic variation on epigenome regulation. Nevertheless, the methods for mapping of CMs are diverse in terms of underlying statistical principles (varying from hierarchical clustering of interindividual peak correlations to Bayesian modeling [17–21]), required input data and format and interpretation of output data. Effective implementation of these approaches requires a profound understanding of underlying computational techniques, including data standardization and experience with handling different data modalities, and accurate interpretation of the results. Moreover, broad application of these methods is hindered due to the lack of streamlined pipelines and associated methodological guidelines.

To address these challenges, we evaluated the existing approaches for CM mapping in terms of methodological and CM characteristics. We assessed the (dis)advantages of each of the methods and provided guidelines related to evaluation criteria, positive controls and data and sample size. We provide executable code that will allow each researcher to execute DO you the workflows on their own data, leading to standardized data output formats and convenient interpretation. Next, we leveraged our method benchmarking efforts to perform CM mapping in six different cell types, revealing that regulatory coordination, captured with CMs, is organized in a cell type-specific manner, with cell type-specific CREs embedded in CMs being hallmarked by binding sites for lineage-specific TFs. Through association with the underlying genotypes and focusing on distinct immune cell types, we identified multiple (autoimmune) disease-associated genetic variants that impact CM activity. These analyses complement canonical chromatin QTL mapping efforts by providing regulatory insights into how non-coding variants contribute to disease by influencing CRE coordination and gene regulation in a cell type-specific manner.

## Results

### Comparative mapping of CMs across individuals with bulk epigenome data

**Fig. 1:**
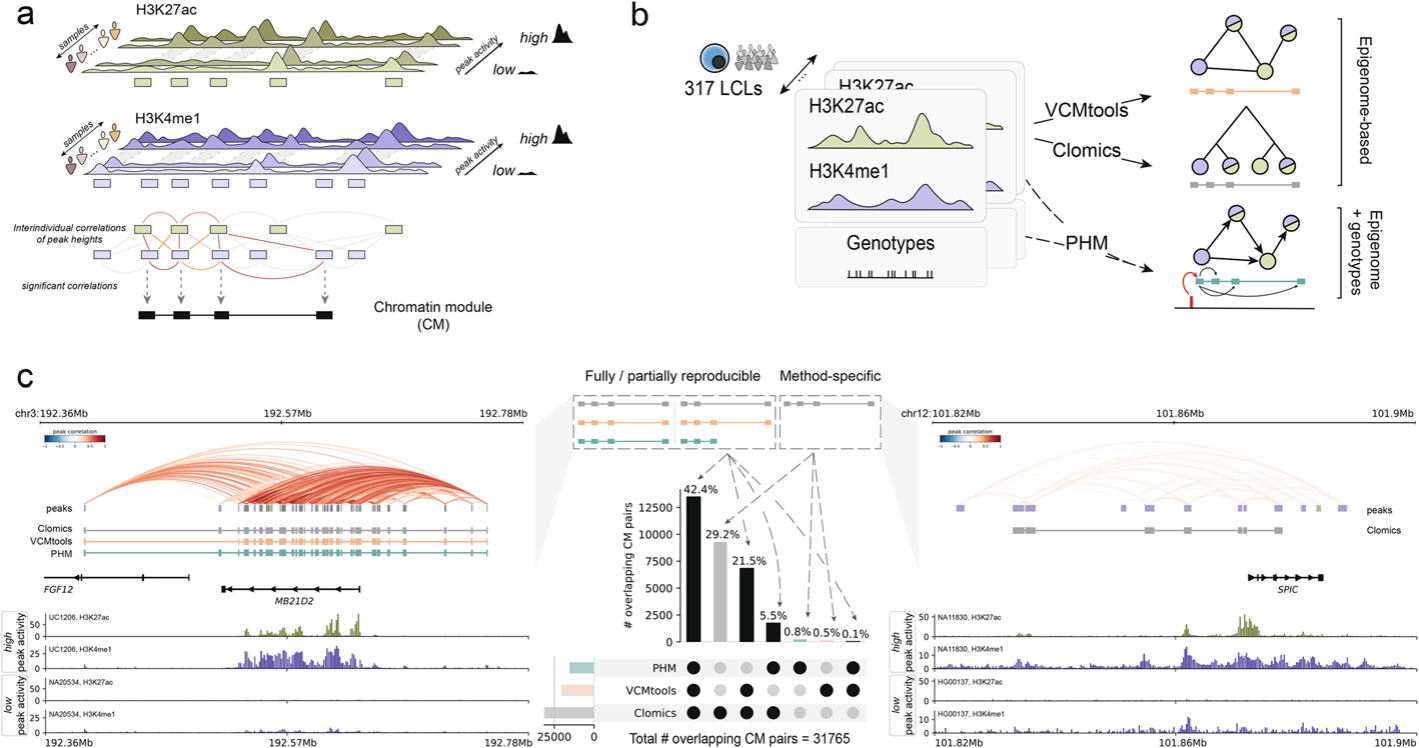
Systematic mapping of chromatin modules (CMs) in LCLs. **a** Schematic representation of ChIP-seq profiles (H3K27ac in green, H3K4me1 in purple) for individuals with differential chromatin activity at the loci. ChIP-seq peaks are shown with purple and green rectangles, where color indicates different histone modifications. Only covariable peaks are used to define a CM, which is depicted with black rectangles at the bottom of the panel. **b** Schematic representation of the pipeline. We collected available H3K27ac and H3K4me1 ChIP-seq data for LCLs and associated genotypes for 317 individuals. We then used the data to map covariable regions with three approaches: correlation-based approaches (VCMtools, Clomics) that depend only on the epigenome data for CM mapping, and a Bayesian hierarchical method (PHM) that requires genotype information in addition to epigenome data. **c** The depicted UpSet plot shows the percentage of overlapping CM pairs by at least one base pair across different methods. Left panel: example of the most reproducible CMs across three methods in the *MD21D2* gene locus. The top track represents peak-to-peak correlations in the locus. The tracks below show CMs mapped with Clomics (gray), VCMtools (orange), and PHM (green). The bottom tracks show ChIP-seq tracks for two individuals with the most differential CM signal (H3K27ac in dark green, H3K4me1 in dark blue). Right panel: example of the Clomics-specific CM (in gray) in the *SPIC* gene locus. The top track represents peak-to-peak correlations; the bottom tracks show ChIP-seq tracks for two individuals with the most differential CM signal

Chromatin modules (CMs) represent genomic regions where CREs display covariable activity for active histone marks such as H3K4me1 and H3K27ac [17] (Fig. 1a). CMs can be mapped using correlation-based approaches that quantify epigenome signal covariability across individuals to group CREs into modules using either graph-based community detection ([18], which we will refer to as VCMtools [22]) or hierarchical clustering (Clomics [19, 20]). A complementary strategy (PHM [21]) uses interindividual variation to infer different modes of associations between peak pairs conditioned on the underlying genetic variant(s) (Fig. 1b). So far, there has been no systematic comparison of existing techniques for CM mapping. To address this, we downloaded available H3K4me1 and H3K27ac ChIP-seq data for 317 lymphoblastoid cell lines (LCLs [19]) together with respective genotype and RNA-seq data [19]. We performed peak calling on all chromosomes for H3K4me1 (218,542 peaks) and H3K27ac (127.060 peaks), and considered H3K4me1 and/or H3K27ac peaks as putative CREs.

We started method performance evaluation by mapping CMs with VCMtools, Clomics, and PHM using the smallest chromosome 22 to evaluate several aspects of computational performance (i.e., elapsed time, memory consumption), and CM-related quantitative outputs (i.e., the number of mapped CMs, median module length, and coefficient of variation of CM length) across various sample sizes (Additional file 1: Fig. S1.1a; “Methods”). Execution-wise, Clomics was the most user-friendly tool (i.e., it required the least input data formatting and was simple to use), the fastest (in terms of elapsed time), and the most efficient in terms of RAM usage, followed by VCMtools and PHM (Additional file 1: Fig. S1.1b, c). Overall, Clomics produced the largest number of CMs yielding two to three times more modules as compared to VCMtools and PHM (Additional file 1: Fig. S1.1d). The number of identified CMs and genetic associations scaled with the number of individuals with the relative gain of CMs reducing after ~175 (Clomics and VCMtools), even though features such as median CM size remained relatively stable from 75 individuals onwards. PHM on another hand showed consistent linear trend with respect to the number of mapped CMs with increasing sample size (Additional file 1: Fig. S1.1d–g). Next, we devised a strategy to quantify the reproducibility of mapped CMs in terms of included CREs, and also quantified how well the methods preserve individual CM peak composition across randomized sample size groups with respect to the number of peaks in CMs (“Methods”). CMs mapped with VCMtools showed the highest average reproducibility across pairwise comparisons of CMs for different sample sizes (Additional file 1: Fig. S1.1h) and exhibited greater consistency in peak composition for smaller CM sizes (2–3 peaks) and lower sample cohorts (Additional file 1: Fig. S1.1i). Clomics and PHM showed a trend toward higher average scores for CMs with a larger number of peaks and larger sample sizes (Additional file 1: Fig. S1.1h, i). All methods revealed higher average reproducibility scores with larger cohort sizes, with the best average reproducibility scores achieved for > 175 (Clomics/VCMtools) or 250 (PHM) samples (Additional file 1: Fig. S1.1h, i). Together, these analyses highlight that CM identification robustness scales with the number of included individuals, only increases marginally when more than 250 individuals are included, and requires a minimum of ~50–75 individuals.

### Evaluation of method and sequencing parameters for CM mapping

To facilitate the use of each method and provide the rationale behind the method selection, we comprehensively evaluated the robustness of each method by testing a range of critical parameters involved in CM mapping. We first assessed how sequencing depth affects each method by downsampling BAM files for each individual to 25, 20, and 10 million reads and mapping CMs with each method (“Methods”). We observed that a large proportion of the CMs mapped across all chromosomes with the full set of reads can be identified with only 10 million reads (Additional file 1: Fig. S1.2a). However, the reproducibility scores for mapped CMs increase from 20 million reads onward (Additional file 1: Fig. S1.2b). The randomization strategy on chromosome 22 for the downsampled data allowed us to show the effect of varying the total number of individuals on the general CM characteristics. For example, the total number of mapped CMs and median CM length showed better resemblance with the full set of reads for higher sequencing depths, thus reinforcing the observation that sample number is a critical parameter for CM mapping for all methods (Additional file 1: Fig. S1.2c–e). We thus recommend to sequence at least 20 million reads per sample, which aligns with the ENCODE recommendations [23].

Next, we evaluated the effect of the two main parameters that can be varied when mapping CMs: (1) the genomic window size left and right of the tested peaks (tested at ranges from 10 kb to 1 Mb) and (2) the cut-off for significance of peak association (*p* value threshold, VCMtools; background correlation, Clomics; posterior probability, PHM). We observed that the number of identified CMs and their size scales inversely with the stringency of the significance cut-off for all methods. For VCMtools and PHM, we observed that the number of CMs and their size is proportional to increasing window sizes (Additional file 1: Fig. S1.3a–c). We complemented this analysis by intersecting the CMs for each parameter pair with their localization in or outside TADs or A/B compartments. This revealed that, for example, with small window sizes (< 100 kb) VCMtools maps many CMs in B (inactive) compartments with limited TAD overlap (Additional file 1: Fig. S1.3d–f). We quantified the reproducibility of the mapped CMs within each method across the range of parameters, which indicated the robustness of Clomics outputs (i.e., an F1 score of overall average reproducibility = 0.545), as compared to VCMtools (F1 score = 0.208) and PHM (F1 score = 0.365). PHM showed the highest reproducibility scores for the posterior probability thresholds > 0.7 across all window sizes, indicating that the approach is robust under stringent cutoffs, whereas VCMtools requires more careful selection of both window sizes and *p* value cutoffs to allow for consistent detection of CMs (Additional file 1: Fig. S1.4). Based on these analyses, and the results summarized in Additional file 1: Figs. S1.2–S1.3, we determined the recommended ranges (indicated with red rectangles in the respective figures) for the significance cutoffs and window sizes yielding reproducible CMs with consistent descriptive statistics (Additional file 3: Table S2).

### Functional relevance of CM localization and embedded genes

Considering the recommended parameter ranges, we mapped CMs on all chromosomes with the full set of individuals using VCMtools (*n* = 9071, window size = 0.5 Mb, *p* value ≤ 0.001), Clomics (*n* = 18,633, window size = 0.5 Mb, background correlation ≥ 3), and PHM (*n* = 5299, window size = 0.5 Mb, posterior probability ≥ 0.8) (Additional file 1: Fig. S1.5a, b; Additional file 2: Table S1; Additional file 3: Table S2). The methods showed high concordance with respect to previously reported CM characteristics [17–21], such as CM length (median 8.6–31.7 kb) and size (median 2–4 peaks) (Additional file 1: Fig. S1.5a), localization (59.4–63.9% CMs fully contained in A (active), compared to 30.5–33.3% for bootstrapped CMs; 25.7–31.9% CMs fully contained in B (inactive) compartments, compared to 50.0–53.9% for bootstrapped CMs; 68.1–73.2% CMs fully contained within TADs, compared to 44.5–49.3% for bootstrapped CMs (Additional file 1: Fig. S1.3d–f, indicated with red rectangles)), enrichment in 3D interactions between CM-embedded CREs (as inferred using either a Hi-C 500 bp or Micro-C 500 bp dataset; Additional file 1: Fig. S1.5d, e), and active chromatin states (Additional file 1: Fig. S1.5f–h) (“Methods”).

Across all overlapping CMs in pairwise method comparisons, 42.4% were at least partially identified using all methods (UpSet plot, Fig. 1c; “Methods”), with the largest overlap between the correlation-based approaches Clomics and VCMtools (Additional file 1: Fig. S1.2b). A representative example of a highly reproducible CM comprises the *MB21D2* locus that was previously reported to display high levels of *cis*-regulatory coordination [21, 24], displaying high or very low ChIP-seq signal in the CM-embedded regions (Fig. 1c, left).

A large proportion of CMs was identified only using Clomics (29.2%). Peaks of Clomics-specific CMs had comparable median statistics of average peak heights to peaks from CMs captured with at least two methods. Yet, peaks of Clomics-specific CMs showed a broader range of average peak heights and their standard deviation across individuals compared to VCMtools- or PHM-specific CM peaks. Together, this suggests that Clomics-specific modules may have a slightly higher power to detect overall smaller covariable peaks compared to VCMtools or PHM (Additional file 1: Fig. S1.5i). A representative example of such a Clomics-specific CM is the *SPIC* locus. Although CM peaks in the *SPIC* locus showed lower average interindividual correlation (cor = 0.18) compared to the CM peaks around *MB21D2* (cor = 0.58), the normalized ChIP-seq signal clearly indicated co-presence or co-absence of CM-embedded peaks (Fig. 1c, right).

Clomics-specific CMs comprised the largest group of method-specific CMs (Fig. 1c, center; Additional file 1: Fig. S1.5c). To assess whether CMs, including method-specific CMs, can aid in advancing our understanding of gene regulation, we assessed the genes that were overlapped by CMs (20–50% among 26,362 protein-coding genes and lincRNAs; 75–80% of CMs overlapped a gene, Additional file 1: Fig. S1.5j, k). We observed that Clomics CMs overlapped in total more gene promoters and gene bodies, also when accounting for the relatively higher number of mapped CMs. Clomics as well captured a higher percentage of significant CM activity (captured as the first principal component of the PCA performed on the CM peak count matrix (aCM [18])) to gene expression correlations compared to VCMtools and PHM (Additional file 1: Fig. S1.6a), which also extends to the Clomics-specific CMs (Additional file 1: Fig. S1.6b). We noted that genes embedded within CMs showed higher coefficient of variation in gene expression with increasing CM size, higher standard deviation, average, and median expression as compared to genes that did not overlap with CMs (Additional file 1: Fig. S1.6c, d). Genes embedded within CMs as well as showed enrichment in B cell and immunity-related terms (Additional file 1: Fig. S1.6e), which was consistent across all approaches. Intersection of the gene categories embedded in CMs (shared or specific to a particular method) revealed that cell type-specific genes are mainly captured in the “shared between methods” groups, and that only Clomics-specific CMs are associated with additional GO terms, which may also be partly related to B cell biology, such as “chronic myeloid leukemia” (Additional file 1: Figs. S1.6e and S1.7a–c). Together, this indicates that Clomics has higher power in detecting more subtle variation in covariable chromatin activity that is associated with changes in gene expression as compared VCMtools and PHM. This is likely due to the adaptive background-aware thresholding used in Clomics that allows to account for subtle yet important differences in local correlation changes compared to the chromosomal background [19, 20], as opposed to universal *p* value thresholding implemented in VCMtools [18].

We have benchmarked a range of thresholds for all CM mapping methods and provide recommended parameter values and ranges (Additional file 3: Table S2). We observed that Clomics was more robust compared to VCMtools for the parameter changes tested. Clomics further stands out by its ease of use and higher sensitivity toward identifying subtle yet putatively relevant chromatin covariation related to changes in gene expression. Together with high reproducibility scores between VCMtools and Clomics, similarity in annotations of the captured genes, and enrichment of Clomics-specific genes in cell type relevant terms, we opt for Clomics over VCMtools among correlation-based approaches. PHM provides directional insights into CRE communication yet requires large sample cohorts and significant computational resources. Therefore, we suggest using PHM as an auxiliary approach for the interpretation of CMs mapped with Clomics, which we will hereafter use for all downstream analyses.

### Cell type specificity of interindividual variation

**Fig. 2:**
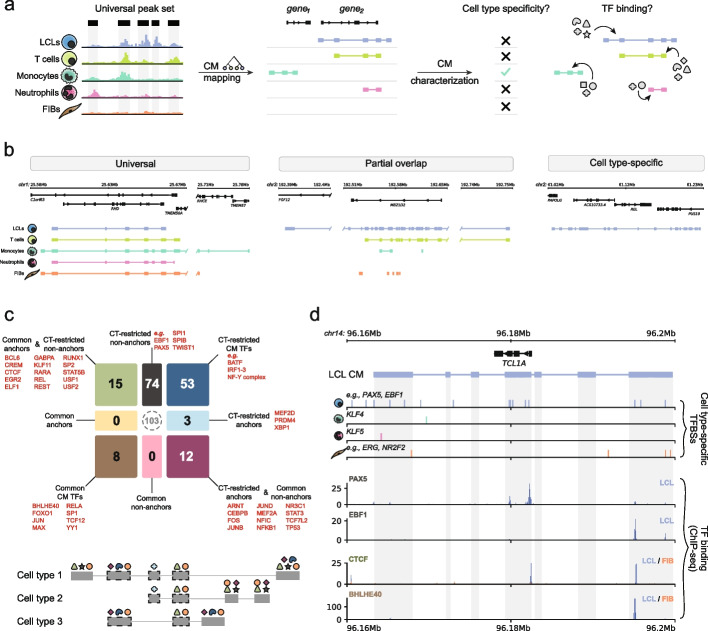
Cell type specificity of regulatory variation and TF binding in CMs. **a** Schematic representation of the pipeline. ChIP-seq and genotype data were collected for hundreds of individuals for five cell types, namely, LCLs, monocytes, neutrophils, T cells, and fibroblasts (FIBs) [19, 28]. The collected ChIP-seq data was processed in a standardized way to obtain a count matrix for a universal peak set (see “Methods” for details), which was used for CM mapping. The downstream analysis included evaluation of cell type specificity of regulatory variation captured in the form of CMs and quantification of TFBS enrichment in specific CM peak types. **b** From left to right: 1. Example of a “universal” CM, here in the *RHD* gene locus where covariable peaks are present in all cell types. 2. Example of a lineage-specific CM in LCLs (blue) and T cells (light green), spanning the *MB21D2* gene. 3. Example of an LCL-specific CM in the *REL* locus. Tracks depicting the ChIP-seq signal at these loci can be found in Additional file 1: Fig. S2.2. **c** TF classification according to TFBS enrichments in distinct categories stratified into anchor versus non-anchor CM CREs and cell type-restricted (CT-restricted) versus common CM CREs based on all pair-wise comparisons together (see “Methods” for details). Bottom: schematic representation of TFBS for different categories of TFs across partially overlapping CMs. **d** Example of an LCL-specific CM (in blue) in the *TCL1A* locus. Tracks below indicate TFBSs (vertical lines) for cell type-enriched TFs per cell type (from top to bottom: LCL: ATF2 (*n* = 1), EBF1 (*n* = 11), KLF1 (*n* = 1), PAX5 (*n* = 5), monocytes: KLF4 (*n* = 1), neutrophils: KLF5 (*n* = 2), FIB: ERG (*n* = 2), NR2F2 (*n* = 1), TFEB (*n* = 1)). No T cell-specific TFBS were found in the locus. The bottom tracks show ENCODE ChIP-seq profiles for PAX5 and EBF1 binding in LCLs, CTCF and BHLHE40 tracks for LCLs (blue) and FIB (orange)

Understanding regulatory variation among individuals in different cell types and states is crucial both from a fundamental and translational perspective [25–27]. To explore cell type specificity of chromatin activity variation among individuals, we extended the LCL dataset with downloaded ChIP-seq (H3K27ac, H3K4me1), genotype (derived from SNP arrays or whole genome sequencing; “Methods”), and RNA-seq data for hundreds of individuals in five cell types: LCLs (*n* = 317), fibroblasts (FIB, *n* = 78), monocytes (*n* = 172), neutrophils (*n* = 164), and T cells (*n* = 93) [19, 28]. To facilitate downstream interpretation and ensure consistency in our analyses, we remapped ChIP-seq peaks to obtain a universal set of peaks for H3K27ac and H3K4me1 in all cell types (Fig. 2a, Additional file 1: Fig. S2.1a; Additional file 4: Table S3; “Methods”) and used this peak set to map CMs with all methods for the data completeness and availability (Additional file 1: Fig. S2.1b; see Availability of data and materials). Based on the preceding analyses, in this and following sections, we specifically focus on CMs mapped with Clomics in LCL (*n* = 18,633), FIB (*n* = 10,158), monocytes (*n* = 9002), neutrophils (*n* = 6604), and T cells (*n* = 4841) (Additional file 1: Fig. S2.1c; Additional file 5: Table S4).

To assess if read count differences were contributing to the differences in total number of identified CMs across cell types, we mapped CMs with the downsampled data of 10, 20, and 25 million reads. We observed that more CMs were mapped in LCLs and FIB than in other cell types, even when we used downsampled BAM files with 10 million reads per sample (Additional file 1: Fig. S2.1d). This might be related to the fact that LCLs and FIB are culture-adapted and more homogeneous, while the primary blood cells (monocytes, neutrophils, and T cells) were taken directly from human donors and may thus be subject to more variability. With the complete set of reads, the highest average similarity was observed for CMs mapped in monocytes and neutrophils, while fibroblasts tended to be the most distant cell type from the other ones, as expected based on its cell lineage (Additional file 1: Fig. S2.1e). We used pairwise cell type comparisons to coarsely categorize CMs into (1) universal CMs (similarity score > 0.7), (2) partially overlapping CMs (0 < similarity score ≤ 0.7), and (3) cell type-specific CMs (similarity score = 0). This showed that the majority of CMs are cell type-specific while some cell types, such as monocytes and neutrophils, have a relatively higher proportion of overlapping CMs (Additional file 1: Fig. S2.1f–j). Together, this demonstrates the prevalent lineage and cell type specificity of the regulatory landscapes captured in form of CMs. Representative loci for each category are shown in Fig. 2b and Additional file 1: Fig. S2.2a, where cell type specificity is also reflected in the association between CM activity and gene expression (Additional file 1: Fig. S2.3a–c).

### CM formation appears to be driven by functionally distinct groups of TFs

To identify candidate TFs associated with CREs embedded within CMs, we first performed differential peak-based TF binding site (TFBSs, which represent the genomic locations of TF motifs matching TF binding sites as determined using ChIP-seq [29]) enrichment analysis between CREs embedded within CMs and non-CM CREs for each cell type (Additional file 1: Fig. S2.3d). We created a set of “simulated” CMs from the non-CM CREs that were size-, distance-, ChIP signal strength-, and GC-matched to the mapped CMs (“Methods”). The TFBS analyses revealed that CREs in CMs are significantly enriched for TFBSs of known cell type-specific TFs, e.g., EBF1 in LCLs [30] and CEBPA/B in monocytes and neutrophils [31] (Additional file 1: Fig. S2.3e–i; “Methods”), with larger similarity between myeloid (monocytes and neutrophils) or lymphoid (LCLs and T cells) cell types (Additional file 1: Fig. S2.3j). Since CMs were mapped based on a universal peak set across cell types, this indicates that CM CREs are enriched for cell type-specific TFBSs.

To further characterize the cell type specificity of the CM-embedded CREs, we assessed the episomal regulatory activity of the CREs using available STARR-seq data from the GM12878 LCL cell line [32]. We performed pairwise comparisons between LCLs and every other cell type to stratify the CM-embedded CREs into those shared between the compared pair of cell types (i.e., CREs that we will refer to as “anchors”) and those specific to one of the cell types (i.e., CREs that we will refer to as “non-anchors”) (Additional file 1: Fig. S2.4a). For all pairwise comparisons of LCLs to other cell types, we observed significantly higher activity at anchor CREs, with the lowest average activity at non-anchors of non-LCL cell types (Additional file 1: Fig. S2.4b). This could indicate that CM CREs that are shared between cell types (anchor CREs) may have higher regulatory potential than non-anchor CREs, which themselves may have more secondary support functions as driven by cell type-specific TFs [33–35]. To test this hypothesis, we first assessed which TFBSs are enriched in non-anchor CREs in a cell type-dependent manner, which revealed the enrichment of TFs with well-known functions in the respective cell types, such as KLF4 in monocytes [36], KLF5 in neutrophils [37], the pioneer B-cell TF EBF1 in LCLs [30], and TWIST1 in FIBs [38] (Additional file 1: Fig. S2.4c–d). These findings suggest that the TFBS enrichment that we observed when comparing the set of reference versus simulated CMs (Additional file 1: Fig. S2.3e–i; “Methods”) is driven by cell type-specific TFs that bind to non-anchor elements.

As a next step, we used all cell types in our pairwise TFBS enrichment analyses to define if non-anchor CRE-enriched TFBSs can also be detected in anchor CREs and if this depends on the assayed cell types. To do so, we specifically focused on the anchor CM CREs and contrasted these with a collection of non-anchor CREs between each cell type pair. Together with the comparison of non-anchor CREs, this allowed us to broadly categorize TFs into several groups based on (1) whether enrichment of their respective binding sites in CMs is specific to either anchors, non-anchors, or whether their binding sites can be found in both (with the latter representing “general” CM TFs), and (2) if this TFBS enrichment is specific to one or few cell types (i.e., “cell type-restricted” (CT-restricted)), or if this enrichment is present in all pairwise cell type comparisons (“common”) (Fig. 2c, Additional file 1: Fig. S2.4e). We identified a total of 165 TFs that are enriched in anchor and/or non-anchor CREs (Fig. 2c; Additional file 6: Table S5). The TFBSs for a total of 23 TFs were always enriched at anchors (indicated with green and brown colors). These TFs include more universally expressed regulators such as USF1/2 and SP1/2, as well as proteins involved in regulating 3D chromatin organization such as CTCF and YY1 [3, 39, 40]. However, we found that the large majority of these 165 TFs (*n* = 130, dark gray, blue, and light blue colors) are enriched in a cell type-restricted fashion in non-anchor or all CM CREs.

Altogether, these analyses suggest that CMs consist of both common regions (anchors) with higher regulatory activity that are preferentially bound by universally expressed TFs as well as CREs that are more cell type-specific and that are bound by TFs relevant to the respective cell type(s) (Additional file 1: Fig. S2.5a, b). A notable example that illustrates how CM formation may be driven by distinct classes of TFs is the B-cell-relevant *TCL1A* locus [41] where the respective gene is specifically expressed in B cells (Additional file 1: Fig. S2.5c). The *TCL1A* gene locus is enriched for binding sites of LCL-enriched TFs in the CM body, especially at the CM peaks, as compared to other cell types. This is exemplified by binding of cell type-specific TFs such as EBF1 and PAX5, as well as LCL-specific binding of CTCF and BHLHE40, at the locus (Fig. 2d, Additional file 1: Fig. S2.5d). Together, our analyses indicate putatively divergent functional roles of TFs in the context of CM formation.

### Chromatin modules capture CREs associated with gene expression

**Fig. 3:**
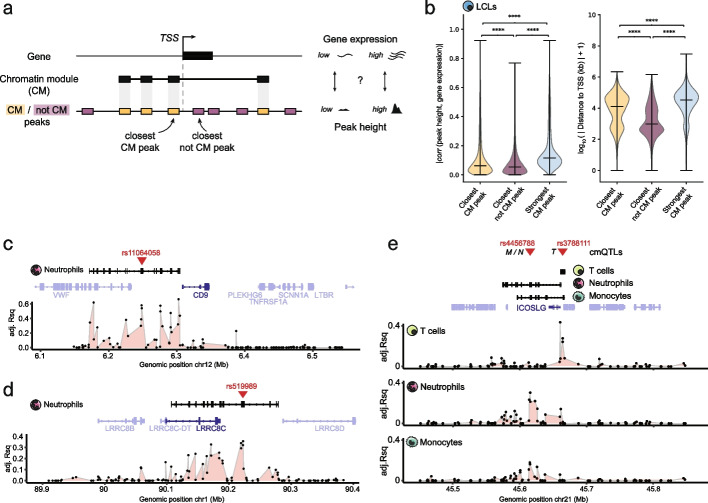
Chromatin modules capture CREs associated with gene expression. **a** Schematic representation of how we identified CM and non-CM peaks closest to the gene transcription start site (TSS) to test the strength of association of either peak height or aCM score with closest gene expression. For each gene, the correlations were computed between gene expression and heights of the closest not CM peaks or closest CM peaks or aCM scores. **b** Violin plots indicating the correlation between each gene expression (for *n* = 26,362 genes) and the closest CM/not CM peak heights (left panel). Median absolute correlation values per category are closest CM peak, 0.063; closest not CM peak, 0.054; and strongest CM peak, 0.115. Distance to the closest peak center from gene transcription start site (TSS) (right panel). For each CM that had a peak closest to the gene, we also found the best-correlating CM peak, which is indicated as the “strongest CM peak” in light blue. **c**–**e** Examples of CMs spanning genes in various cell types having a cmQTL (red triangles). The tracks below CMs and genes show the association strength (adjusted *R*^2^ (Rsq) of the linear regression) between every peak in the locus and expression of the gene highlighted in dark blue

We observed that Clomics allows detecting many significant associations of aCM score with gene expression (Additional file 1: Fig. S1.6a). Together with identified TFs that are enriched within CMs and that as such may contribute to their establishment, we next aimed to conceptually assess how CMs can aid in understanding gene regulation. It is conceivable that CMs offer several advantages compared to single CREs, since (i) they group CREs into collaborating hubs, (ii) the activity state of the CM (aCM) can be derived which reflects the compound activity of all CM-embedded CREs and thus that of the locus, and (iii) CREs are linked to genes based on co-activity profiles and thus do not require 3D information for this. We first aimed to assess to what extent the aCM score is associated to gene expression relative to that of individual CREs (Fig. 3a). Specifically, for each gene, we correlated its expression with (1) the height of the peak closest to the TSS but not part of the respective CM, (2) the height of the CM-embedded peak closest to the TSS, and (3) the activity score of the CM closest to the TSS (“Methods”). This revealed that CM CREs show higher (albeit still modest) correlations with expression of the closest gene at larger genomic distances compared to non-CM-embedded CREs (Fig. 3b, Additional file 1: Fig. S3.1a–d). This difference is more pronounced in the gain of significant associations to gene expression when using aCM scores (Additional file 1: Fig. S3.1e–i), which, we found, is unrelated to the total sequencing depth (Additional file 1: Fig. S3.1j) with overall associations being significantly higher when the promoter of the tested gene is embedded in the CM compared to the gene body or no overlap (Additional file 1: Fig. S3.1k). In addition, absolute correlation values between aCM scores and expression of the closest genes were on average higher than absolute correlation values between the heights of individual CM peaks and expression of the closest genes, largely independent of which CM peak was assessed (Additional file 1: Fig. S3.1l–p). Together, by using multiple covarying CREs aggregated into this aCM score, the set of candidate genes for which their respective expression correlates with chromatin state activity can be expanded by at least twofold (Fig. S3.2a–e).

CMs are detected by leveraging interindividual variation, so it is conceivable that genetic variation contributes to the activity of at least a portion of CMs [18, 19] and through the CM, affects gene expression. We therefore mapped not only histone mark (H3K4me1 and H3K27ac) QTLs (hQTLs; Additional file 7: Table S6), but also variants that affect the activity of CMs (cmQTLs; Additional file 8: Table S7). While the total number of hQTLs is higher, QTLs could be mapped for a higher proportion of CMs compared to histone marks (~10% compared to ~40–50% for CMs; Additional file 1: Fig. S4.1a, b). We observed that ~60–70% of cmQTLs are also hQTLs or in LD (*R*^2^ > 0.8) with hQTLs, indicating that in a large number of cases, variants disrupt a CM by disrupting histone mark enrichment at one or multiple CREs (Additional file 1: Fig. S4.1c), and they do so in a largely cell type-specific manner (Additional file 1: Fig. S4.1d). While it is conceivable that variants in LD with cmQTLs affect other peaks, the majority of them are also located in peaks of the same CM and within a small genomic window of these focal cmQTLs (~600 bp for *R*^2^ = 1). This suggests that the observed variation of nearby peaks is not directly mediated by variants in LD with cmQTLs (Additional file 1: Fig. S4.1e), consistent with earlier observations [22]. Binning of the interindividual peak signal variability further showed that CREs with higher interindividual variation in peak signal are more likely to be associated to a QTL and/or embedded in a CM, and that CM mapping allows to capture almost twice as many variable peaks compared to conventional hQTLs (Additional file 1: Fig. S4.1f). This suggests that variable peaks that are less strongly associated with a genetic variant can still be captured in CMs through their dependency on the activity of another peak/CRE within the same CM. This is consistent with observations based on in silico approaches which suggest that frequently one or two CREs may act as the “dominant” or “lead” element(s) resulting in increased chromatin activity at the lead element itself as well as at nearby CREs [21]. Given that approximately half of the CMs can be linked to a candidate causal variant (at least with the significance cut-off used here, Additional file 1: Fig. S4.1a, g–h), it is conceivable that such QTLs disrupt the lead CRE in a locus leading to both local and distal changes in the chromatin landscape. As is evident from the examples highlighted in Fig. 3c–e and Additional file 1: Fig. S3.2f–j, there are generally two or three CREs at which the ChIP-signal is strongest associated with expression of the nearby gene, in line with previous observations using CRISPR interference [42, 43]. The associated cmQTLs localize in one of the peaks with the strongest association (Adj. *R*^2^ of linear regression between peak height and gene expression; Fig. 3c–e; Additional file 1: Fig. S3.2f–j, red triangles), which therefore represents a putative “lead” regulatory element in a genomic region. For example, in the *CD9* locus in neutrophils, we observed that six principal regions correlate with *CD9* expression (Adj. *R*^2^ > 0.4 and FDR < 0.05 in linear regression) (Fig. 3c). All these elements are embedded into a CM, and the genetic variant with the strongest association to CM activity localizes in the peak ~50 kb upstream of the *CD9* promoter, indicating that this is the putative lead CRE for the CM. Another example comprises the *LRRC8C* locus, where we identified a cmQTL (rs519989) for the CM in neutrophils localized in an intergenic enhancer ~25 kb upstream of *LRRC8C* gene (Fig. 3d). The rs519989 variant was recently shown to impact PU.1 binding to this enhancer, leading to reduced enhancer-promoter connectivity and lower expression of *LRRC8C* [44]. These examples illustrate how CM mapping can aid in identifying causal variants, linking them to genes in the respective locus without any 3D chromosome conformation information and obtaining a fine-grained understanding of the regulatory mechanisms underlying (cell type-dependent) gene expression.

### cmQTLs are associated with binding of cell type-specific TFs

Next, we assessed the cell type specificity of cmQTLs and observed that hQTLs were more frequently hQTLs in another cell type (or in LD (*R*^2^ > 0.8) with hQTLs) compared to cmQTLs. For example, 31.6% of H3K27ac hQTLs vs 19.4% of cmQTLs were shared between monocytes and neutrophils, indicating that cmQTLs capture more cell type-specific activity, as also revealed based on the enriched TFBSs (Fig. 2; Additional file 1: Fig. S4.1d). As both hQTLs and cmQTLs can provide regulatory cues by disrupting or creating TFBSs, we compared histone QTLs that are also cmQTLs (which we will refer to as “hcmQTLs” for simplicity) to QTLs that are only associated with a histone modification peak (hQTLs). Both QTL types displayed a similar genomic distribution (Additional file 1: Fig. S4.1i) with hcmQTLs having on average a stronger association with the peak height (i.e., higher beta values in a linear regression of genotype and normalized peak counts; Additional file 1: Fig. S4.1j). Both QTL types were enriched in cell type-specific open chromatin regions, with slightly higher specificity for hcmQTLs (Additional file 1: Fig. S4.1k), and hcmQTLs displayed stronger overlap (or in LD (*R*^2^ > 0.8) with cell type-specific expression QTLs (eQTLs; Additional file 1: Fig. S4.1l)). Given that CM CREs enriched for TFBSs associated with cell type-specific TFs, we assessed if hcmQTLs would also more often disrupt TFBSs for cell type-specific TFs compared to hQTLs. Comparison of the TF binding events that are disrupted by the QTLs using allele-specific binding (ASB) analysis [45] (“Methods”) revealed that a higher proportion of hcmQTLs is associated with ASB in any cell type compared to hQTLs with associations of QTLs with ASBs being partially cell type-specific (Additional file 1: Fig. S4.2a). Notable examples include enrichments in ASB for the myeloid master regulator CEBPA/B in monocytes and neutrophils [31], for the mesoderm TF TCF21 in fibroblasts [46], and the T(h1) and lymphoid factor TBX21 in T cells [47] (Additional file 1: Fig. S4.2b). In addition, many of the ASB events were more frequent in the hcmQTL group (Additional file 1: Fig. S4.2b). We complemented these analyses by assessing which TFs are more often binding (based on ChIP-seq data in any cell type) in a 200 bp window around the hcmQTL compared to hQTLs [48]. This validated the ASB observations in terms of binding of cell type-enriched TFs at hcmQTLs, such as IRF4 and BCL6 TFs in LCL, JUN/FOS in fibroblasts, and C/EBP in monocytes and neutrophils (Additional file 1: Fig. S4.2c). Together, these observations further indicate that CMs tend to arise in a cell type-specific manner driven by the binding of cell type-related TFs.

### Deconstructing regulatory hierarchies at autoimmune disease GWAS loci using CMs

**Fig. 4:**
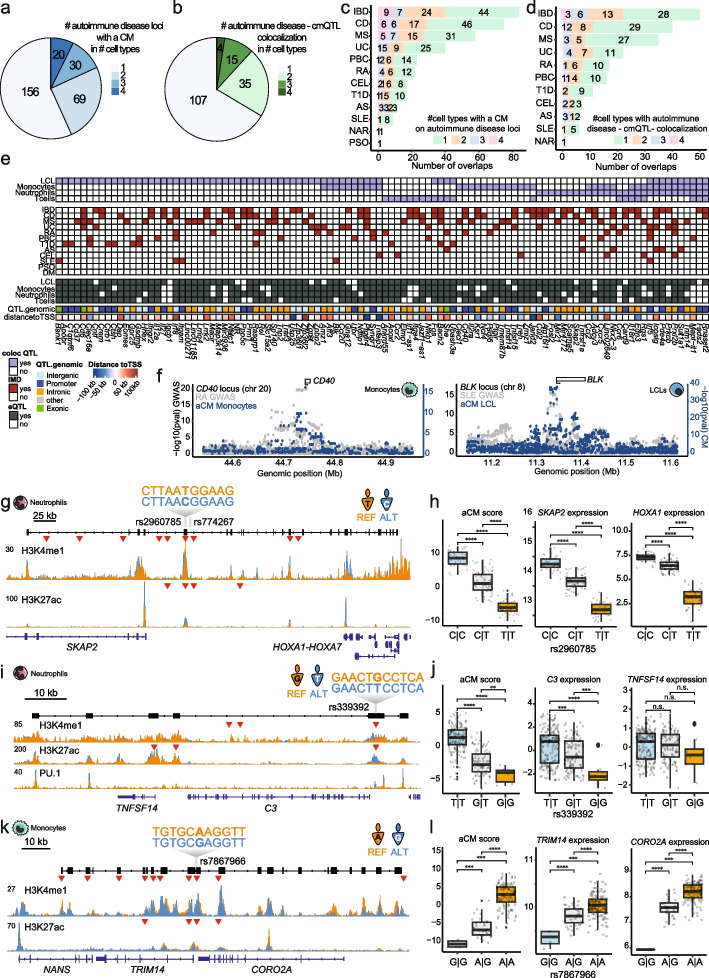
Mapping the regulatory logic at autoimmune disease risk loci using CMs. **a** The number of autoimmune disease-associated GWAS loci where a CM was mapped in any of the four immune cell types. **b** The number of loci where the cmQTL colocalized with a GWAS QTL for least one autoimmune disease (posterior probability (PP) > 0.8 and at least one variant that has a *p* value of 1e-5 for both GWAS and variant-aCM association). **c** The number of loci where a CM was mapped categorized per autoimmune disease. **d** The number of loci with a cmQTL-GWAS colocalization categorized per autoimmune disease. **e** Summary overview of autoimmune disease risk loci that harbor a CM with the associating cmQTL colocalizing with the GWAS variant for at least one disease. The color legend can be found at the lower left of the panel. Abbreviations are spondylitis (AS), celiac disease (CEL), Crohn’s disease (CD), juvenile dermatomyositis (DM), inflammatory bowel disease (IBD), multiple sclerosis (MS), primary biliary cirrhosis (PBC), psoriasis (PSO), rheumatoid arthritis (RA), systemic lupus erythematosus (SLE), type 1 diabetes (T1D), and ulcerative colitis (UC). **f** Left: example of colocalization of rheumatoid arthritis (RA) GWAS signal and the cmQTLs at the *CD40* locus in monocytes. Right: example of colocalization of systemic lupus erythematosus (SLE) GWAS signal and cmQTLs at the *BLK* locus in LCLs. **g** Example depicting the *SKAP2* locus in neutrophils, one example individual per genotype. **h** CM activity, *SKAP2* and *HOXA1* expression stratified by genotype of the highest-ranked candidate-associated variant rs2960785. **i** Example depicting the *C3* locus in neutrophils, one example individual per genotype. **j** CM activity, *C3* and *TNFSF14* expression stratified by genotype of the highest-ranked candidate-associated variant rs339392. **k** Example depicting the *TRIM14* locus in monocytes, one example individual per genotype. **l** CM activity, *TRIM14* and *CORO2A* expression stratified by genotype of the highest-ranked candidate-associated variant rs7867966. In **g**, **i**, and **k**, the red triangles represent the localizations of histone QTLs. In the boxplots, each dot represents one individual and *p* values were calculated using a Wilcoxon test. *p* value indications are non-significant (ns) for *p* value > 0.05, * for 0.01 < *p* value ≤ 0.05, ** for 0.001 < *p* value ≤ 0.01, *** for 0.0001 < *p* value ≤ 0.001, and **** *p* value ≤ 0.0001

Compared to hQTLs, we found that hcmQTLs have a stronger association with peak height, disrupt peaks that are most strongly associated with gene expression, and are more likely to overlap cell type-specific eQTLs. Given these attributes, we reasoned that hcmQTLs could be used to improve our understanding of disease predisposition. We computed the overlap (shared variants or in LD with *R*^2^ > 0.8) of hcmQTLs and hQTLs with variants in the GWAS catalog [49] and compared the observed/expected ratio for the two types of QTLs relative to the GWAS catalog. Several associations occurred more frequently for hcmQTLs in the tested cell type, such as “monocyte counts” in monocytes, “lymphocyte counts” in T cells, and “neutrophil percentage of white cells” for neutrophils (Additional file 1: Fig. S4.3a), further underlining the cell type specificity of cmQTLs. To assess how cmQTLs can assist in understanding regulatory logic at disease loci, we focused on autoimmune disease as these have their origin in immune cell types and a large number of GWAS summary statistics is available. We used a list of 340 GWAS loci where co-localization of GWAS has been observed with at least an eQTL, hQTL, methylation QTL, or a splicing QTL [50]. We assessed co-localization of the cmQTLs on these loci with GWAS summary statistics for ankylosing spondylitis (AS), celiac disease (CEL), Crohn’s disease (CD), juvenile dermatomyositis (DM), inflammatory bowel disease (IBD), multiple sclerosis (MS), primary biliary cirrhosis (PBC), psoriasis (PSO), rheumatoid arthritis (RA), systemic lupus erythematosus (SLE), type 1 diabetes (T1D), and ulcerative colitis (UC) [51–61]. The majority of these loci (*n* = 275, 80.9%) contained a CM in at least one cell type (Fig. 4a). Bayesian co-localization analyses [62] revealed co-localization (posterior probability (PP) > 0.8 and *p* value 1e−5 for both cmQTL and GWAS association) of 59% (*n* = 161) of cmQTLs with GWAS signal for at least one autoimmune disease (Fig. 4b; Additional file 1: Fig. S4.3b), with more than half of the mapped CMs and cmQTL-GWAS co-localization being cell type-specific and the large majority (in LD with) an eQTL [28] in at least one tested cell type (Fig. 4c, d). We aggregated the co-localizations on a per-gene basis as one gene can be associated with multiple GWAS variants, resulting in 106 GWAS gene—cmQTL co-localizations (Fig. 4e, some representative examples of co-localizations in Fig. 4f). A notable example is the *SKAP2* locus with two candidate QTLs (rs2960785 and rs774267) associated with the aCM score and expression of the CM-embedded genes *SKAP2* and *HOXA1* in neutrophils (Fig. 4g, h). However, only rs2960785 localizes inside an intergenic enhancer region between *SKAP2* and *HOXA1*, indicating that rs2960785 is the likely cmQTL. In addition, the association of rs2960785 with gene expression is highly blood-enriched (Additional file 1: Fig. S4.3c); it creates a putative GABPA binding site (CGGAAG) [63] (which is a direct interactor of the myeloid TF CEBPA [64]) and is associated with ASB of CEBPA in AML [45]. These findings provide direct insights into the molecular mechanisms that likely underlie how this genetic variant may impact the locus in a cell type-specific manner. For the *C3* and *TNFSF14* locus, the cmQTL rs339392 localizes in the *C3* promoter where it is associated with chromatin activity at the locus, the expression of *C3* itself, and to some extent also the expression of *TNFSF14* in LCLs (Fig. 4i, j). The variant is also an eQTL for C3 in LCLs (Additional file 1: Fig. S4.3c) and is predicted to create a PU.1/ETS binding motif (Additional file 1: Fig. S4.3d). Rs339392 was recently confirmed as a PU.1 binding QTL in LCL [65], and this impact on PU.1 binding extends to the other peaks embedded within the CM (Fig. 4i). This shows how a DNA base change can impact both histone modifications and TF binding on the focal CRE as well as associated distal CM-embedded CREs. A final example is the *TRIM14* locus, where the variant rs7867966 in the *TRIM14* promoter is associated with the aCM score and the expression of *TRIM14* as well as the nearby gene *CORO2A* (Fig. 4k, l). Rs7867966 is an eQTL specific to blood cells (Additional file 1: Fig. S4.3c) and predicted to disrupt a C/EBP binding site (Additional file 1: Fig. S4.3e). Together, these example loci conceptually illustrate how mapping of CMs and associated QTLs can be used for the mechanistic interpretation of disease-associated non-coding variants. In addition, it may allow to narrow down the number of candidate variants within a locus, as frequently more than one hQTL per locus can be identified (Fig. 4g, i, k; red triangles).

### Chromatin modules reveal how genetic variants may impact expression of immune surface markers

Surface markers represent the molecules essential for immune cell interactions and consequently function, and several associations between non-coding variants and cell surface marker expression have already been reported [66]. Altered regulation of surface marker expression can affect how immune cells interact to combat infection and also impact the efficacy of treatment modalities that target these markers such as immunotherapy regimes. We aimed to use CMs to assess how QTLs may affect surface marker regulation and expression. We used a list of 185 surface markers consisting of all cluster of differentiation (CD) and tetraspanin molecules [67]. For 69 of these loci, we identified a CM in at least one cell type, and for 44 loci, a cmQTL was found in at least one cell type (Additional file 1: Fig. S4.4a). For example, for the CM spanning *CD207* in monocytes, the top associated cmQTL rs11126300 localizes in an intergenic enhancer region between *CD207* and *CLEC4F*, where it modulates the epigenome of a range of putative CREs as well as the expression of both *CLEC4F* and *CD207* (Additional file 1: Fig. S4.4b–d). Another relevant example is the *CD93* locus in neutrophils, where a CM spans several genes. The top ranked cmQTL rs844881 localizes in a peak at the 3′ UTR of the lncRNA *LINC00656* and has a strong association with the activity of the CM and the expression of *THBD*, *CD93*, and *LINC00656*. We did not observe an expression change for *NXT1* and *NAPB*, which are adjacent genes that are not part of the respective CM (Additional file 1: Fig. S4.4e–g). The variant rs844881 shows a strong association with the development of varicose veins in the FinnGen cohort (*p* = 5.5e−5) [68]. This could be related to the impacted expression of the *THBD* gene, as altered *THBD* expression in neutrophils has been implicated in the development of venous thrombosis [69] and thus together provides a putative mechanism of how rs844881 could impact the risk on varicose veins by impacting *THBD* expression in neutrophils. Finally, another example of the *CD9* locus shows that even though the same genetic variant impacts an intergenic enhancer in different cell types, it is only when adjacent CREs are included in the CM that this results in an impact on gene expression (Fig. 3c, Additional file 1: Fig. S4.4h, i).

Altogether, these analyses reveal how genetic variants could impact CMs around surface markers and potentially result in functional variation in immune cell function and disease susceptibility.

### Context-dependent formation of CMs in chronic lymphocytic leukemia

**Fig. 5:**
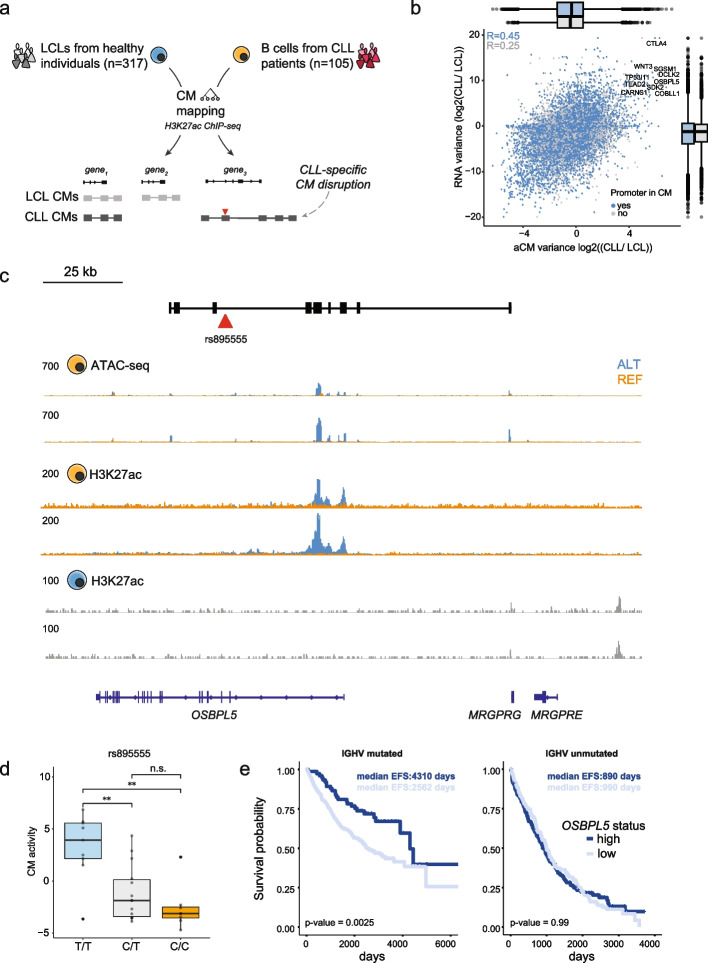
Activation of genomic loci in a subset of CLL patients. **a** Strategy for mapping CMs using available ChIP-seq for H3K27ac in LCL (*n* = 317) and CLL (*n* = 105) samples. **b** Log2 ratio of interindividual variance of aCM activity (x-axis) and interindividual variance in expression of the gene(s) embedded within the CM. Colors represent whether the promoter of the gene is part of the CM. Correlation values are Pearson correlation. Boxplots represent the distribution of the interindividual variance. **c** Example depicting the *OSBPL5* locus whose embedded gene’s expression is induced in a subset of CLL patients. **d** CM activity stratified by genotype of the candidate-associated variant rs895555. **e** Event-free survival of CLL patients stratified on *OSBPL5* expression for both IGHV-mutated and -unmutated CLL status. *p* values were obtained using a log-rank test. *p* value indications are non-significant (ns) for *p* value > 0.05, * for 0.01 < *p* value ≤ 0.05, and ** for 0.001 < *p* value ≤ 0.01

Recent studies revealed that non-coding genetic variants can impact the prognosis of chronic (CLL) and acute (ALL) lymphocytic or myeloid (AML) leukemia [70–72]. A variant in the *AXIN2* locus was shown to activate multiple enhancers (within a CM), the expression of *AXIN2*, and was linked to better survival rates of CLL patients [22]. A variant in a *GATA3* enhancer was shown to impact multiple regions around GATA3, resulting in higher *GATA3* expression in *cis* and downstream 3D genome rearrangements [72]. We thus assessed if CMs capture CLL-specific-induced epigenome configurations. We uniformly analyzed epigenome data from LCLs (immortalized B cells in a non-disease context, *n* = 317 individuals) and CLL (immortalized B cells in the context of cancer, *n* = 105 individuals) (Fig. 5a). As for the five initial cell types (Fig. 2a), we defined a common set of peaks based on H3K27ac and used these for CM mapping. We then calculated the variance of each CM in both LCL and CLL in combination with the interindividual variability in gene expression to identify genomic loci in which the local chromatin environment and expression of the embedded genes are distinct between LCL and CLL. This analysis, in combination with stringent filtering, allowed identification of 16 prioritized candidate loci in CLL (Fig. 5b; Additional file 1: Fig. S5.1a, b), which include previously implicated genes such as *CTLA4* and *WNT3* [73, 74] (Fig. 5b). We found that the locus-gene combination most specific to CLL is the *OSBPL5* locus (Fig. 5b), where the *OSBPL5* promoter and several flanking enhancers are activated in a subset of CLL patients (Fig. 5c). Association to the underlying genotype (*n* = 35 individuals with both H3K27ac ChIP-seq and genotype data available [22]) identified a candidate non-coding germline variant (rs895555, MAF = 45%) in an *OSBPL5* intron that is strongly associated with the activation of the locus (Fig. 5d, Additional file 1: Fig. S5.1c). The T allele of rs895555 creates the core of a putative Forkhead (FOX) TF binding site ((T/C)GTTT) (Additional file 1: Fig. S5.1d), which is of interest given the implication of this TF family in the development and progression of B cell malignancies [75]. *OSBPL5* expression is a prognostic marker for overall survival in the context of mutated immunoglobulin heavy variable (IGHV) gene status (Fig. 5e) and was recently identified as the strongest predictive gene expression marker for time to progression after CLL treatment [76]. The second-highest variable gene-locus combination was the *COBLL1* locus (Additional file 1: Fig. S5.2a–c), where the strongest associated variant is a common (MAF = 17%) intronic TATA duplication. *COBLL1* expression is associated with survival in chronic lymphocytic leukemia (Additional file 1: Fig. S5.2d) and has also been linked to survival probability in several other cancer types including chronic myeloid leukemia [77]. Finally, we analyzed TF binding profiles in the CREs included in CLL or LCL CMs, compared to simulated reference CMs. We observed enrichment of many similar types of TFs, related to B cell function such as IRF, RUNX, and EBF1 (Additional file 1: Fig. S5.2e, f), but also specific enrichment of factors such as FOXO1 (as also seen for rs895555) in CLL which have been implicated in CLL and harbor driver mutations [75]. Despite the limited number of genotypes available for CLL, we show that CMs can be used to identify candidate variants associated with CLL. Since CMs capture covariable CREs, such an approach provides a conceptual framework as to how genetic variants may molecularly impact gene expression and consequently cellular state.

## Discussion

In this work, we established computational guidelines to map CMs and harmonize the output formats across the available methods VCMtools, Clomics, and PHM, which makes CM mapping accessible to the community (see Availability of data and materials). For each method, we provide recommendations regarding parameter ranges and optimal values (Additional file 3: Table S2). Based on the analyses presented here, we recommend to use Clomics as compared to VCMtools and PHM since it (1) has higher power also in the context of lower sample sizes and more subtle peak-peak associations; (2) identified CMs that show no major dependency on parameter tuning in terms of total number, size, or overlap with TADs and A/B compartments; (3) captures a higher percentage of aCM-gene expression correlations; and (4) is easier and faster to run. VCMtools and PHM may still be used as confirmatory analyses to further define the most reproducible CMs, as the only limitation of Clomics is that the output CMs may show limited changes depending on the cohort of individuals included as evident in the sub-sampling analyses.

We thus used Clomics to comprehensively assess regulatory coordination in six cell types and hundreds of individuals in a genome-wide manner, which revealed extensive cell type specificity of regulatory variation. We provide mapped CMs in an accessible format (see Availability of data and materials) and show how these can be used to disentangle the different (epi)genomic modalities, starting from genetic variants to TFBSs, histone modifications on local and distal CREs and finally gene expression. Our results substantiate the hypothesis of cooperation between local and distal CREs within TADs, which we observed using both epigenome data and enrichment in 3D interactions. At the sub-TAD scale, we argue that mapping of CMs thus provides an approach complementary to 3C-based applications for determining CRE interactions within TADs in a genome-wide manner [17–19, 21]. An advantage of mapping CMs is that CREs are grouped into functional units, and that CMs therefore allow to provide mechanistic rationales regarding how non-coding variants can impact the epigenome of local and distal CREs as well as the expression of genes embedded within the respective CM. We provide examples of such analyses in the case of GWAS loci for autoimmune disease, surface marker expression variation on immune cells, and identification of candidate variants driving prognostic marker expression in CLL patients.

Ultra-resolution 3C-based assays or microscopy strategies have revealed in specific loci that the removal of the canonical proteins involved in regulating 3D genome architecture, such as CTCF and cohesin, has limited impact on the establishment of interactions between CREs [78–83]. Moreover, structural proteins such as CTCF are ubiquitously expressed and tend thus on themselves to be unable to define the cell type-specific CRE cooperativity within CMs. Together, this raises questions of how regulatory interactions within a TAD environment are established in a cell type-specific context. An attractive hypothesis is that this is mediated by cooperating TFs, which often are expressed in a cell type- or lineage-specific manner [84], followed by TF-mediated recruitment of general co-activators and chromatin modifiers [17]. The observations on the cell types assayed here suggest that CMs are established by classes of more ubiquitously expressed TFs that bind CM-embedded CREs that are shared across multiple cell types (here defined as anchors) in combination with cell type-specific TFs that bind CM-embedded CREs whose activity is restricted to one of just a few cell types. Such cooperative binding of cell type-/lineage-specific and core CM TFs [35] that reflect a specific cell state may thus mediate CRE interactions within and beyond the 3D genome organization established by CTCF and Cohesin. This observation is also consistent with the notion that cmQTLs frequently disrupt the binding sites of cell-type specific TFs, which then could affect histone modification deposition and cooperation between CREs within a CM in a cell type-specific manner. This finding complements recent observations showing that genetic variants impact CRE interactions relevant for establishing gene expression through direct perturbation of TF binding to enhancers [85] and that TFs are the main drivers of gene expression cooperativity [86].

## Conclusions

Altogether, we demonstrate that CMs provide a means to shed light on the mechanisms underlying gene expression and gene regulatory variation with high-throughput epigenome data in a range of genetic backgrounds, provided the availability of a sufficient number of assayed samples. Future applications of CM mapping could include defining CM plasticity within closely related cell types during cellular differentiation, or rather in different species to assess how conserved local genome organization is. With the constantly decreasing cost of high-throughput sequencing and easy-to-implement epigenome tools, we advocate for the value of CM mapping in future studies where epigenome data is being generated in large sample cohorts as part of the overarching goal to understanding how regulatory variation contributes to complex traits and disease [18, 19, 28, 66, 87]. We believe that the guidelines, executable code, and interpretable output provided here will highly facilitate this process.

## Methods

### ChIP-seq, ATAC-seq, and RNA-seq data processing

Unpaired reads were removed using samtools [88] and duplicates were marked and removed using picard v2.17.8 [89]. To create a common set of peaks per cell type, we followed a commonly used strategy [19] and created a “meta” BAM file per epigenome assay (H3K4me1, H3K27ac, or ATAC) by merging (samtools *merge*) downsampled (15 million reads per sample) bam files (for details on downsampling strategy, see next section) for each individual. The resulting meta bam files of the different cell types were then merged per epigenome assay resulting in the creation of the final merged bam files. Peak calling was performed on these files using MACS2 [90] using parameters “--broad” “-f BAMPE” and a *q* value cutoff of 0.01. The obtained universal peak set was used to count the number of reads for the respective epigenome assays per individual per cell type using FastReadCounter (https://github.com/DeplanckeLab/FastReadCounter). The resulting non-normalized count matrices were RPKM normalized using the *rpkm* function from edgeR v3.36.0 [91]. For data standardization, we used linear regression to remove the known covariates sex and age. As the LCL cohort consists of a merge of cohorts from individuals from different ancestries, the first 3 principal components of the genotypes (obtained using QTLtools pca [92]) were removed as well. To account for effects of unknown covariates, principal component analyses were performed on the RPKM-normalized count matrices and the first 10 PCs (in case of monocytes, neutrophils, T cells, and CLL) or the first 20 PCs (in case of LCL and FIB) were also removed during the regression [19]. The residuals from the linear regression were kept and normalized using quantile normalization with the qqnorm function from the stats base R package. Throughout the manuscript, we considered all the peaks for H3K27ac and H3K4me1 that were determined using the strategy described above as CREs, as these chromatin marks in essence denote active regulatory regions. The common set of peaks we created is identical for all cell types. Hence, CREs (peaks) have the same coordinates across cell types, yet vary in their chromatin mark enrichment, which, as hypothesized, is driven by cell type-specific gene regulatory programs. The resulting matrices were used as input for mapping of QTLs and CMs.

RNA-seq.fastq files for LCL and FIB [19] were processed in a same way as the epigenome data, with variance stabilization using the DESeq2 R package (v 1.40.1) [93] instead of RPKM normalization. The normalized RNA-seq data for monocytes, neutrophils, and T cells was obtained from the original study (see below Availability of data and materials) [28].

To minimize inconsistencies in TF expression measurements in RNA-seq data from different studies and cell types, we used the Human Protein Atlas (HPA) data (see below Availability of data and materials) [84]. We used RNA Monaco immune cell gene data to extract TF expression values for the immune cell types as protein-transcripts per million (pTPM). Namely, for “naive CD4 T-cell” for T cells, “naive B-cell” for LCLs, “neutrophil” for neutrophils, “classical monocyte” for monocytes, and RNA GTEx tissue gene data to extract pTPM values for “skin” tissue, which we used as a proxy to FIBs.

### ChIP-seq downsampling and read statistics

Total read counts for BAM files were extracted using samtools *idxstats* [88], which was used to calculate the ratio between the target number of reads (10, 20, or 25 million) and the total read number (defined in Additional file 4: Table S3). If the BAM file contained less reads than the target read number, no downsampling was performed and BAM files were used as a whole. When more reads were present, the BAM files were downsampled with samtools *view* using *-s* parameter with the ratio of the total to the target number of reads. The universal peak set was then used to generate the count matrix for the respective downsampled BAM files per individual per cell type using FastReadCounter (https://github.com/DeplanckeLab/FastReadCounter). The resulting non-normalized count matrices were processed in the same way as the count matrices constructed for the original BAM files without downsampling (see above).

### CM mapping

The methods used in this work required adjustments (or full implementation, as in the case of VCMtools).

- The CM mapping approach described in Waszak et al. [18] was implemented in python (hereafter referred to as VCMtools) and adapted to account for the cell type-specific background by using empirical *p* value correction followed by FDR thresholding. Namely, for each chromosome, for two peaks, whose centers are located at the max distances of 500 kb from each other, we calculated and stored correlation values of the respective peak height profiles across individuals. Then, we calculated the average background correlation (${\mu }_{bg}$) and its standard deviation (${\sigma }_{bg}$) for all peak pairs across all chromosomes. This allowed us to calculate the empirical *p* value as 1—scipy.stats.norm.cdf (correlation, loc = ${\mu }_{bg}$, scale = ${\sigma }_{bg}$), which we further corrected with the Benjamini-Hochberg procedure. CMs were identified by selecting isolated components after filtering peak-to-peak correlations based on the corrected *p* value threshold (*p* value ≤ 0.001).
- To map CMs with Clomics, we used the default window size of 200 peaks for calculating peak correlations and the default background correlation cut-off of 3. Clomics did not require major changes except for the conversion of the output tree file into a BED file format.
- We used the default window size for PHM (1 Mb centered at the focal peak) and by following the described methods in the supplementary section of Kumasaka et al. [21]. We implemented some of the key methods not included in the PHM package (https://github.com/natsuhiko/PHM/), including the DAG construction for inference of hierarchies between peaks categorized into the causality hypothesis. In the current study, for the reconstruction of the hierarchies we considered peak pairs with posterior probability of causal interaction ≥ 0.8.

Data preprocessing and CM mapping scripts are available via GitHub (https://github.com/DeplanckeLab/Chromatin_modules).

### Randomization strategy for investigating the number of samples required to map CMs

The robustness of the CM mapping methods was evaluated with respect to the number of individuals in every LCL on the chromosome chr22. We chose the smallest chromosome chr22 and ran each method on a subset of samples of size $s\in \left\{x\times 25 | x=1, 2, \dots , 12\right\}$, where each of the subsets was randomly sampled five times, resulting in 12 × 5 runs per method. For each execution run, we measured the number of CMs, elapsed time, memory consumption, median module length, coefficient of variation of CM length, and percentage of CMs with a cmQTL.

### Parameter selection for CM mapping

For each method, there were two critical parameters to test: the maximum distance between a peak pair (CREs) that is considered for association testing and the strength of such association.

- To select the window size, we started with the commonly used distance of 0.5 Mb as the reference point. We would like to note here that in Additional file 1: Figs. S1.3 and S1.4 the window size of 0.5 Mb refers to the distance from a focal peak to any another peak to its left or right, which is equivalent to using a 1 Mb window (e.g., in the PHM definition) centered at the focal peak. Therefore, the tested window sizes from 0.01 to 1 Mb to the left/right of the peak correspond to respectively 0.02–2 Mb windows centered at peaks. We selected the 0.25 Mb step to ensure a diverse range of window sizes while avoiding extensive search space due to its computational burden in the case of multiple parameter combinations. Clomics is somewhat different in concept in terms of how it is designed and it uses a defined number of peaks around a focal peak rather than a specific genomic window. To obtain a similar window search space to VCMtools and PHM, we picked the range from 25 to 400 peaks (with the default of 200 peaks approximating a 1 Mb window [19]).
- To choose the peak association threshold per method, we also aimed to test a range of options from the least to the more stringent ones. In case of VCMtools, we started with the 0.05 *p* value threshold and chose a few more stringent options up to 1e−6. One could possibly test intermediate thresholds, such as 0.005 and 0.0005, yet we decided to exclude those from consideration to speed up computations for all pairwise combinations of tested parameters. Clomics background correlation thresholds, as well as the PHM ones, were also selected in such a way to ensure a broad range of tested values and respective outcomes. It is thereby important to note that in the original PHM study [21], the authors used a 0.5 posterior probability threshold to map causal peak interactions in ATAC-seq data for 100 LCL samples. To map such causal peak interactions in ChIP-seq data, however, we observed that such threshold is quite lenient, which motivated us to implement more stringent values and to select 0.8 as our “default” option.

The current parameter search space allowed us to reach the low and high extremes of the measured statistics (e.g., for the distribution of sizes and number of mapped CMs). We also defined parameter combinations per method, which can be found in Additional file 3: Table S2.

### Reproducibility analysis of mapped CMs across cell types and methods

To compare CMs, we followed the approach based on the harmonic mean score [94]. We started by calculating the Jaccard overlap between the peaks at the level of base pairs. Then, for each pair of overlapping CMs $$\left(C{M}_{i}, C{M}_{j}\right)$$, where $$C{M}_{i}=\{{p}_{i}^{1},{p}_{i}^{2},\dots ,{p}_{i}^{n}\} \,\text{and}\, C{M}_{j}=\{{p}_{j}^{1},{p}_{j}^{2},\dots ,{p}_{j}^{m}\}$$, we created a matrix of size $$n\times m$$, where $$n$$ is the number of peaks in $$C{M}_{i}$$ and $$m$$ is the number of peaks in $$C{M}_{j}$$. If there is an overlap between a $${p}_{i}^{k}$$ and $${p}_{j}^{l}$$, then the value in the $$k$$ th row and $$l$$ th column of the matrix $$J$$ is defined as a Jaccard index $${J}_{k,l}=\frac{{|p}_{i}^{k}\cap {p}_{j}^{l}|}{{|p}_{i}^{k}\cup {p}_{j}^{l}|}$$, where $${p}_{i}^{k}$$ and $${p}_{j}^{l}$$ are defined as sets of unique genomic positions. For a pair of CMs, we first calculated the average of maximum values along the rows ($$r)$$ and columns ($$c)$$ of matrix $$J$$, and then the final score (which we refer to as F1 score) as a harmonic mean $$\frac{2}{\frac{1}{r}+\frac{1}{c}}$$.

### CM overlap with A/B compartments, TADs, chromatin state annotations, and genes

TAD and A/B compartment (start, end) coordinates (for GM12878, GSE63525) were overlapped with CM coordinates (CM start and end) using bedtools [95] function *intersect* with the *–wo* to report the base pair overlap of CMs with the regions of interest. Prior to intersecting CMs and TADs, overlapping or nested TADs were merged into a single TAD region by taking the minimum and the maximum coordinates among the regions of interest. Next, based on the CM overlap size (with TADs or A/B compartments) with respect to the CM length, we split CMs into those that were fully located in TADs or A/B compartments, partially overlapping ones (part of a CM falls outside of the region yet overlaps it by at least one base pair) and the CMs completely falling outside the regions of interest. To see whether the statistics for obtained overlaps are different from the expected values, we followed the bootstrapping strategy. Namely, we shuffled the CMs 1000 times along the genome using the bedtools [95] *shuffle* function with varying *-seed* parameter set to the iteration number of the shuffling round. We also restricted the shuffling to individual chromosomes by using the *-chrom* parameter and prevented shuffled regions falling into blacklisted regions by using the *-excl* parameter followed by the list of regions (https://github.com/Boyle-Lab/Blacklist/blob/master/lists/hg19-blacklist.v2.bed.gz) [96]. The randomized CM coordinates were overlapped with TADs and A/B compartments following the previously described strategy. Based on the average number of shuffled CMs overlapping TADs, or A/B compartments respectively, we obtained 95% confidence intervals (CI) for each method. The CIs for CMs fully falling into TADs per method were Clomics CI = [0.48, 0.49], VCMtools CI = [0.48, 0.5], and PHM CI = [0.44, 0.47]. The CIs for CMs fully falling into A compartments per method were Clomics CI = [0.32, 0.33], VCMtools CI = [0.31, 0.33], and PHM CI = [0.3, 0.33]. The CIs for CMs fully falling into B compartments per method were Clomics CI = [0.52, 0.53], VCMtools CI = [0.52, 0.54], and PHM CI = [0.5, 0.53]. CRE annotations for LCLs (GM12878) were obtained from ChromHMM [10] and SCREEN (all human cCREs, hg38; coordinates were lifted to hg19) [97] databases. Annotated CRE coordinates were overlapped with CM and non-CM peaks with bedtools without restrictions on the overlap size. Then, per each CRE annotation category and per peak, we calculated the fraction of peak length overlapping with the respective annotated CRE. For each CRE category, we performed Fisher’s exact test on the contingency table constructed for CM peaks and non-CM peaks in a category of interest (non-significant (ns) for *p* value > 0.05, * for 0.01 < *p* value ≤ 0.05, ** for 0.001 < *p* value ≤ 0.01, *** for 0.0001 < *p* value ≤ 0.001, **** *p* value ≤ 0.0001).

Gene coordinates were downloaded from the GRCh37 (hg19) assembly. For every gene, we defined the promoter region as +−500 bp from the TSS, and gene body as gene coordinates without promoter region. Gene coordinates (promoter start, gene body end) were overlapped with CM peaks with bedtools [95] *intersect* with the *–wo* to report the base pair overlap of CMs with the gene coordinates, without restrictions on the overlap size (1 bp was considered sufficient). If CM peaks overlapped more than one gene, the statistics were calculated for every CM-gene pair. To calculate the coefficient of variation (CV) of CM-embedded gene expression, we used counts per million ((CPM)-normalized counts). To explore the differences between standard deviation, mean and median gene expression of genes embedded within CMs or genes outside of CMs, and all other aCM-gene correlation analyses, we used regressed out, variance stabilized and DESeq2 normalized RNA-seq data (see “ChIP-seq, ATAC-seq, and RNA-seq data processing”). For the aCM/peak height to gene correlation analyses, we found the closest gene TSS to the entry of interest with bedtools [95] *closest*, and then proceeded with correlating either the aCM score or peak height with the closest gene expression. If several genes were equidistant, we considered all of them in the analyses. We calculated the coefficient of correlation together with the associated *p* value (using *scipy.stats.pearsonr*), which were further corrected with the Benjamini-Hochberg procedure. Associations with the corrected *p* value ≤ 0.05 were considered significant.

### Calculation of the CM activity score (aCM)

For each CM, the embedded peaks and associated counts per individual were extracted to create a separate peak by individual matrix, followed by principal component analysis. Since the embedded peaks are correlated by definition, most variation in signal between the individuals is captured in the first principal component, and thus the values for this component were extracted and used as the aCM score.

### Quantification of peak interactions in Hi-C and Micro-C data

Micro-C data was aligned using BWA v0.7.17 [98]. Ligation events were determined using pairtools parse with parameters *--min-mapq 40 --walks-policy 5unique --max-inter-align-gap 30* [99]. PCR duplicates were removed using pairtools *dedup* followed by the generation of BAM and pairs files. The.pairs files were used to generate cooler files using *cooler cload pairix* with default parameters [100]. For the constructed set of paired mapped and simulated CMs, interaction frequencies for peaks in CMs were quantified in Hi-C and Micro-C data at 500 bp, 1 kb, and 5 kb resolutions by first fetching the bins overlapping individual peaks and then averaging the signal within the bin overlap area for all possible peak pairs in a CM.

### Simulation strategy for CMs

To move away from a single or paired element-defined background, we devised a simulation strategy to construct a background set of CMs resembling mapped regions of covariable CREs in terms of their intrinsic properties, which include:

1. Number of regulatory elements in a CM
2. Length of a CM
3. Total base pair length of elements in a CM
4. Overall peak signal variability in a CM
5. H3K27ac peak fraction
6. CM location in A or B compartments and TADs (optional)

Prior to simulating the background set of CMs, we define the reference set of CMs as the set of all CMs if A/B compartment and TAD data (pt. 6) is not available. For the consistency of the analysis described in the paper, we did not use the A/B compartment and TAD constraint since the data was not available for all cell types. In the case when this data is available, the reference set of CMs is defined by overlapping the mapped CMs with TADs and stratifying the CMs based on their localization in A/B compartments. This allows to narrow down the set of CMs to putative functional regions by conditioning on TADs and account for potential structural differences of CMs located in A or B compartments.

Every CM in the reference set is characterized by a vector of features described above in points 1–6. Then, for every CM from the reference set of size *n*, we randomly sampled a peak from the non-CM peak set, and *n*−1 peaks in the peak neighborhood. In the case of simulation of CMs for the PHM output, we started by randomly sampling a peak classified into one of the single QTL hypotheses. The number of peaks per histone mark in the set of sampled non-CM peaks was perfectly matched with the respective reference CM histone mark peak frequencies. We repeated the procedure 10 times per CM, resulting in 10 sets of sampled peaks per CM, where each set represents a candidate simulated CM. These candidates for simulated CMs were characterized with the same feature set as for CMs. For every CM in the reference set, we searched for the five most similar simulated CMs among all generated candidates of the same size (same number of peaks), with respect to the feature vector, by applying Approximate Nearest Neighbors search (Annoy package https://github.com/spotify/annoy). After matching CMs with simulated CMs, we filtered out simulated CMs with more than 10% variation in GC content, CM length, and total base pair length of peaks as compared to the reference CM. This allowed us to obtain the final set of paired mapped and simulated CMs.

### Differential TFBS enrichment and TF expression

We used command line UniBind TFBS differential enrichment tool (*UniBind_enrich.sh* script with *twoSets* parameter and *hg38_robust_UniBind_LOLA.RDS* motif set) [101]. Prior to running the script, peak coordinates were lifted from *hg19* to *hg38*. Depending on the task, the background set was changed, e.g., for differential TFBS enrichment in actual CM peaks vs simulated CM peaks, we used simulated CM peaks as a background set.

For TFBS enrichment at CM peaks in cell type A vs cell type B (Additional file 1: Fig. S2.4), and classification of TFs into cell type-restricted/common anchors/non-anchors, we considered all pairwise combinations of cell types. We started with splitting the peaks into CM and not CM peaks per cell type, and merged the overlapping peaks (e.g., coming from different histone modifications) into a single peak to prevent the same region being considered for TFBS enrichment several times. Based on the CM and not CM peaks in cell type A, and CM and not CM peaks in cell type B, we defined anchor and non-anchor peaks for a cell type pair of interest. Namely, if CMs peaks from two cell types were overlapping by at least 1 bp, we defined them as anchors, otherwise non-overlapping CM peaks between two cell types were defined as non-anchors. Prior to running the differential TFBS enrichment analysis, we lifted peak coordinates from *hg19* to *hg38*. Next, we performed differential TFBS enrichment analysis with the UniBind TFBS differential enrichment tool (*UniBind_enrich.sh* script with *twoSets* parameter and *hg38_robust_UniBind_LOLA.RDS* motif set) for (1) anchor vs non-anchor (union of cell type-specific) CREs, (2) non-anchor CREs in cell type A vs non-anchor CREs in cell type B, and (3) non-anchor CREs in cell type B vs non-anchor CREs in cell type A. A TFBS is considered significantly enriched by UniBind if the observed overlap in tested regions is greater than expected by chance, as compared to the background, defined as a merge of input sets. It is important to note that UniBind uses the same background set for differential enrichment analysis in both directions: cell type A vs cell type B and cell type B vs cell type A. Together with the intrinsic input dataset properties (cell types from close lineages), this methodological choice can lead to some TFs appearing significantly enriched in both comparisons. For each comparison group, we obtained a table with a TF and respective *q* value for enrichment in a given set vs defined background. Significantly enriched TFBSs (*q* value ≤ 1e−06) obtained through this analysis were additionally filtered based on TF expression (protein-transcripts per million (pTPM) > 2 in at least one cell type [84]). Finally, we overlapped the groups of TFs across all pairwise cell type comparisons to define the final TF categories (Fig. 2c).

### Mapping of quantitative trait loci (QTLs)

VCF files with genotype information for LCL and FIB samples were created based on SNP arrays (8,245,940 SNPs [19]), and those for monocytes, neutrophils, and T cells based on whole genome sequencing (6,914,843 SNPs [28]). Mapping of QTLs was done using QTLtools [92] in the QTLtools *cis* mode with the options --permute 1000 and --normal. For each peak or CM, all variants within a 1 Mb around the tested phenotype were considered. In practice, this means that several thousand genetic variants were tested for each phenotype. To account for the fact that genotype-phenotype associations are assayed for a large number of loci, the resulting FDR-adjusted *p* values were further corrected using *q* value correction using the qvalue R package (v2.24.0, https://github.com/StoreyLab/qvalue). Variants with an FDR-*q* value corrected *p* value under 0.05 were considered significant.

### Intersection of QTLs with GWAS

To allow for a complete overlap, the variants in linkage disequilibrium (LD) with the QTLs were determined using plink2 [102] based on the 1000G genotype file for individuals with a European ancestry. All variants within a 1 Mb window were considered and those with *R*^2^ > 0.8 retained as LD variants. The GWAS catalog for hg19 with 392,271 associations (release 2022-05-11) was downloaded from the UCSC table browser and only variants that were present in the genotype file and the GWAS catalog were considered for downstream analyses. To compare histone QTLs that were also cmQTLs with hQTLs, the hQTL files were split into variants that were also cmQTLs themselves or in LD (*R*^2^ > 0.8) with cmQTLs (from then on referred to as hcmQTLs) and only hQTLs. Then, variants were overlapped with the GWAS variant file and considered overlapping based on a direct match or LD (*R*^2^ > 0.8) with a GWAS variant. This yields the quantification of how many overlaps there are of the tested QTL set with each GWAS variant. By comparing this with the QTL set size and the total occurrence in the GWAS file, this allows to obtain the observed/expected ratio for each QTL set individually. A Fisher’s exact test was used to compare this ratio between hcmQTL and hQTL sets to identify the enriched terms. For colocalization of cmQTLs with GWAS variants, each variant in a 500 kb window around the CM was tested for the association with the aCM score. The resulting variants were matched with those present in the summary statistics for each of the tested autoimmune disease-associated variants. Colocalization was performed using Coloc v5.2.2 [62]. Variants with a posterior probability (PP4) > 0.8 and a *p* value under 1e−5 for both the cmQTL and GWAS associations were considered as colocalizing.

### Intersection of QTLs with TF binding

QTLs were divided in hcmQTLs and hQTLs and 200 bp windows were created around each variant. The R package ReMapEnrich v0.99.0 [48] was used to identify TFs that were found to bind in these windows using ChIP-seq in any human cell type. Enrichment was assessed using the hcmQTL set as input with the hQTL-only set as reference, thus serving as reference regions. Allele specific binding (ASB) events for 1073 TFs were downloaded from the Adastra database (release June 2022) [45]. For each TF, only ASB events with a reported FDR < 0.05 were retained. Identified hQTLs were stratified in hcmQTLs and hQTLs and intersected with the ASB events to identify the total number of ASB events for each TF for the two QTL groups. The number of ASBs for each TF was compared between the QTL groups using a Fisher’s exact test. Prediction of TF motif disruption by QTLs was done using position weight matrices (PWMs) [103].

### Enrichment of QTLs in open chromatin regions

QTLs were divided in hcmQTLs and hQTLs and analyzed using Forge2 [104] via the associated web server (https://forge2.altiusinstitute.org/) using default parameters. Consolidated Roadmap Epigenome DNAseI hypersensitive sites were used for the enrichment analysis.

### Supplementary Information

Additional file 1. Supplementary figures (Figs. S1.1–S5.2).

Additional file 2: Table S1. Chromatin modules mapped in LCLs using VCMtools, Clomics, and PHM.

Additional file 3: Table S2. Evaluation of parameters for VCMtools, Clomics, and PHM.

Additional file 4: Table S3. Read statistics per cell type.

Additional file 5: Table S4. Chromatin modules mapped in five cell types using Clomics.

Additional file 6: Table S5. Categorization of TFBSs in CM CREs.

Additional file 7: Table S6. hQTLs in five cell types.

Additional file 8: Table S7. cmQTLs for chromatin modules in five cell types.

Additional file 9. Peer review history.

## Data Availability

Raw ChIP-seq and RNA-seq.fastq files for LCL and fibroblasts were downloaded from the ArrayExpress Archive at EMBL-EBI (www.ebi.ac.uk/arrayexpress) (E-MTAB-3656 and E-MTAB-3657) and the European Genome-phenome Archive (European Genome-phenome Archive; https://ega-archive.org/datasets/) (EGAD00001000428, EGAD00001004790, EGAD00001004871, EGAD00001004872) [18, 19]. Monocytes, neutrophils, and T cell ChIP-seq.fastq (EGAD00001002670, EGAD00001002672, EGAD00001002673) and genotype data (EGAD00001002663) were obtained from the European Genome Archive [28]. ChIP-seq and ATAC-seq data for CLL were downloaded from the European Genome Archive (EGAD00001004046) [87]. Processed RNA-seq files for monocytes, neutrophils, and T cells were downloaded from ftp://ftp.ebi.ac.uk/pub/databases/blueprint/blueprint_Epivar/ [28]. Processed RNA-seq files for CLL were downloaded from http://resources.idibaps.org/paper/the-reference-epigenome-and-regulatory-chromatin-landscape-of-chronic-lymphocytic-leukemia [87]. Genotypes from the 1000 Genomes were obtained from https://www.internationalgenome.org/data, namely, via https://ftp.1000genomes.ebi.ac.uk/vol1/ftp/release/20130502/ [105]. All autoimmune disease summary statistics were obtained from the GWAS catalog (https://www.ebi.ac.uk/gwas/) [49]. Hi-C contact matrices, A/B compartments, and TADs for GM12878 were downloaded from GEO (GSE63525) [3]. Processed ultra-high resolution Hi-C data for GM12878 at 500 bp (ENCSR410MDC) was obtained from ENCODE [106]. Micro-C data for GM12878 Micro-C 800 M was obtained from Dovetail Genomics (https://micro-c.readthedocs.io/en/latest/data_sets.html) (https://cantatabio.com/dovetail-genomics/). CRE annotations for LCLs (GM12878) were obtained from EpiMap (https://personal.broadinstitute.org/cboix/epimap/ChromHMM/observed_aux_18_hg19/CALLS/) [107] for ChromHMM and SCREEN database (all human cCREs, hg38; coordinates were lifted to hg19) [97]. Blacklisted regions for hg19 were downloaded from https://github.com/Boyle-Lab/Blacklist/blob/master/lists/hg19-blacklist.v2.bed.gz [96]. TF expression data was obtained from the Human Protein Atlas, namely, “RNA Monaco immune cell gene data” and “RNA GTEx tissue gene data” [84]. STARR-seq data from the GM12878 LCL cell line was obtained from GEO (GSE104001), namely, the GSE104001_HiDRA_counts_per_fragmentgroup.txt.gz file [32]. ChIP-seq tracks for TFs in the bigwig format for signal *p* value of merged replicates were downloaded from ENCODE portal (ChIP-seq -> Transcription Factor) [108] for GM12878 and IMR-90 for the following TFs: EBF1 (ENCFF248XJC for GM12878), PAX5 (ENCFF759XQV for GM12878), BHLHE40 (ENCFF429WGS for IMR-90, ENCFF816QSI for GM12878), CTCF (ENCFF583IZF for IMR-90, ENCFF749HDD for GM12878). All scripts for mapping CMs using VCMtools, Clomics, or PHM are publicly available at GitHub (https://github.com/DeplanckeLab/Chromatin_modules) [109] and Zenodo (https://zenodo.org/records/12600812) [110]. All mapped CMs and associated QTLs can be browsed using the webserver (reachable at chromo.epfl.ch) associated to this manuscript.

## References

[1] Lieberman-Aiden E, van Berkum NL, Williams L, Imakaev M, Ragoczy T, Telling A (2009). Comprehensive mapping of long-range interactions reveals folding principles of the human genome. Science.

[2] Dixon JR, Selvaraj S, Yue F, Kim A, Li Y, Shen Y (2012). Topological domains in mammalian genomes identified by analysis of chromatin interactions. Nature.

[3] Rao SSP, Huntley MH, Durand NC, Stamenova EK, Bochkov ID, Robinson JT, et al. A 3D map of the human genome at kilobase resolution reveals principles of chromatin looping. Cell. 2014. GSE63525. https://www.ncbi.nlm.nih.gov/geo/. 10.1016/j.cell.2014.11.021 PMC5635824 25497547

[4] Zuin J, Roth G, Zhan Y, Cramard J, Redolfi J, Piskadlo E (2022). Nonlinear control of transcription through enhancer-promoter interactions. Nature.

[5] Javierre BM, Burren OS, Wilder SP, Kreuzhuber R, Hill SM, Sewitz S (2016). Lineage-specific genome architecture links enhancers and non-coding disease variants to target gene promoters. Cell.

[6] Freire-Pritchett P, Schoenfelder S, Várnai C, Wingett SW, Cairns J, Collier AJ (2017). Global reorganisation of cis-regulatory units upon lineage commitment of human embryonic stem cells. Elife.

[7] Osterwalder M, Barozzi I, Tissières V, Fukuda-Yuzawa Y, Mannion BJ, Afzal SY (2018). Enhancer redundancy provides phenotypic robustness in mammalian development. Nature.

[8] Hay D, Hughes JR, Babbs C, Davies JOJ, Graham BJ, Hanssen L (2016). Genetic dissection of the α-globin super-enhancer in vivo. Nat Genet.

[9] Oudelaar AM, Davies JOJ, Hanssen LLP, Telenius JM, Schwessinger R, Liu Y (2018). Single-allele chromatin interactions identify regulatory hubs in dynamic compartmentalized domains. Nat Genet.

[10] Ernst J, Kellis M (2017). Chromatin-state discovery and genome annotation with ChromHMM. Nat Protoc.

[11] Degner JF, Pai AA, Pique-Regi R, Veyrieras J-B, Gaffney DJ, Pickrell JK (2012). DNase I sensitivity QTLs are a major determinant of human expression variation. Nature.

[12] McVicker G, van de Geijn B, Degner JF, Cain CE, Banovich NE, Raj A (1979). Identification of genetic variants that affect histone modifications in human cells. Science.

[13] Kilpinen H, Waszak SM, Gschwind AR, Raghav SK, Witwicki RM, Orioli A (1979). Coordinated effects of sequence variation on DNA binding, chromatin structure, and transcription. Science.

[14] Gaffney DJ, Veyrieras J-B, Degner JF, Pique-Regi R, Pai AA, Crawford GE (2012). Dissecting the regulatory architecture of gene expression QTLs. Genome Biol.

[15] Lappalainen T, Sammeth M, Friedländer MR, ‘tHoen PAC, Monlong J, Rivas MA (2013). Transcriptome and genome sequencing uncovers functional variation in humans. Nature.

[16] Grubert F, Zaugg JB, Kasowski M, Ursu O, Spacek DV, Martin AR (2015). Genetic control of chromatin states in humans involves local and distal chromosomal interactions. Cell.

[17] van Mierlo G, Pushkarev O, Kribelbauer JF, Deplancke B (2023). Chromatin modules and their implication in genomic organization and gene regulation. Trends Genet.

[18] Waszak SM, Delaneau O, Gschwind AR, Kilpinen H, Raghav SK, Witwicki RM, et al. Population variation and genetic control of modular chromatin architecture in humans. Cell. 2015. E-MTAB-3656, E-MTAB-3657. www.ebi.ac.uk/arrayexpress. 10.1016/j.cell.2015.08.001 26300124

[19] Delaneau O, Zazhytska M, Borel C, Giannuzzi G, Rey G, Howald C, et al. Chromatin three-dimensional interactions mediate genetic effects on gene expression. Science (1979). 2019. E-MTAB-3656, E-MTAB-3657. www.ebi.ac.uk/arrayexpress; EGAD00001000428, EGAD00001004790, EGAD00001004871, EGAD00001004872. https://ega-archive.org/datasets/, https://github.com/odelaneau/clomics. 10.1126/science.aat8266 31048460

[20] Avalos D, Rey G, Ribeiro DM, Ramisch A, Dermitzakis ET, Delaneau O (2023). Genetic variation in cis-regulatory domains suggests cell type-specific regulatory mechanisms in immunity. Commun Biol.

[21] Kumasaka N, Knights AJ, Gaffney DJ. High-resolution genetic mapping of putative causal interactions between regions of open chromatin. Nat Genet. 2019. https://github.com/natsuhiko/PHM. 10.1038/s41588-018-0278-6 PMC6330062 30478436

[22] Llimos G, Gardeux V, Koch U, Kribelbauer JF, Hafner A, Alpern D (2022). A leukemia-protective germline variant mediates chromatin module formation via transcription factor nucleation. Nat Commun.

[23] Landt SG, Marinov GK, Kundaje A, Kheradpour P, Pauli F, Batzoglou S (2012). ChIP-seq guidelines and practices of the ENCODE and modENCODE consortia. Genome Res.

[24] Kumasaka N, Knights AJ, Gaffney DJ (2016). Fine-mapping cellular QTLs with RASQUAL and ATAC-seq. Nat Genet.

[25] Nasser J, Bergman DT, Fulco CP, Guckelberger P, Doughty BR, Patwardhan TA (2021). Genome-wide enhancer maps link risk variants to disease genes. Nature.

[26] Jagadeesh KA, Dey KK, Montoro DT, Mohan R, Gazal S, Engreitz JM (2022). Identifying disease-critical cell types and cellular processes by integrating single-cell RNA-sequencing and human genetics. Nat Genet.

[27] Taylor CA, Watson RA, Tong O, Ye W, Nassiri I, Gilchrist JJ (2022). IL7 genetic variation and toxicity to immune checkpoint blockade in patients with melanoma. Nat Med.

[28] Chen L, Ge B, Casale FP, Vasquez L, Kwan T, Garrido-Martín D, et al. Genetic drivers of epigenetic and transcriptional variation in human immune cells. Cell. 2016. EGAD00001002670, EGAD00001002672, EGAD00001002673, EGAD00001002663. https://ega-archive.org/datasets/, ftp://ftp.ebi.ac.uk/pub/databases/blueprint/blueprint_Epivar/. 10.1016/j.cell.2016.10.026 PMC5119954 27863251

[29] Gheorghe M, Sandve GK, Khan A, Chèneby J, Ballester B, Mathelier A (2019). A map of direct TF-DNA interactions in the human genome. Nucleic Acids Res.

[30] Boller S, Li R, Grosschedl R (2018). Defining B cell chromatin: lessons from EBF1. Trends Genet.

[31] Pundhir S, Bratt Lauridsen FK, Schuster MB, Jakobsen JS, Ge Y, Schoof EM (2018). Enhancer and transcription factor dynamics during myeloid differentiation reveal an early differentiation block in cebpa null progenitors. Cell Rep.

[32] Wang X, He L, Goggin SM, Saadat A, Wang L, Sinnott-Armstrong N, et al. High-resolution genome-wide functional dissection of transcriptional regulatory regions and nucleotides in human. Nat Commun. 2018. GSE104001. https://www.ncbi.nlm.nih.gov/geo/. 10.1038/s41467-018-07746-1 PMC6300699 30568279

[33] Ibarra IL, Hollmann NM, Klaus B, Augsten S, Velten B, Hennig J (2020). Mechanistic insights into transcription factor cooperativity and its impact on protein-phenotype interactions. Nat Commun.

[34] Rao S, Ahmad K, Ramachandran S (2021). Cooperative binding between distant transcription factors is a hallmark of active enhancers. Mol Cell.

[35] Kribelbauer JF, Pushkarev O, Gardeux V, Russeil J, Mierlo G van, Deplancke B. Context transcription factors establish cooperative environments and mediate enhancer communication. bioRxiv. 2023:2023.05.05.539543. 10.1038/s41588-024-01892-7 PMC11525195 39363017

[36] Feinberg MW, Wara AK, Cao Z, Lebedeva MA, Rosenbauer F, Iwasaki H (2007). The Kruppel-like factor KLF4 is a critical regulator of monocyte differentiation. EMBO J.

[37] Shahrin NH, Diakiw S, Dent LA, Brown AL, D’Andrea RJ (2016). Conditional knockout mice demonstrate function of Klf5 as a myeloid transcription factor. Blood.

[38] García-Palmero I, Torres S, Bartolomé RA, Peláez-García A, Larriba MJ, Lopez-Lucendo M (2016). Twist1-induced activation of human fibroblasts promotes matrix stiffness by upregulating palladin and collagen α1(VI). Oncogene.

[39] Wang W, Qiao S, Li G, Yang C, Zhong C, Stovall DB, et al. A histidine cluster determines YY1-compartmentalized coactivators and chromatin elements in phase-separated super-enhancers. bioRxiv. 2021:2021.09.15.460559. 10.1093/nar/gkac233 PMC9122595 35390165

[40] O’Connor L, Gilmour J, Bonifer C (2016). The role of the ubiquitously expressed transcription factor Sp1 in tissue-specific transcriptional regulation and in disease. Yale J Biol Med.

[41] Herling M, Patel KA, Weit N, Lilienthal N, Hallek M, Keating MJ (2009). High TCL1 levels are a marker of B-cell receptor pathway responsiveness and adverse outcome in chronic lymphocytic leukemia. Blood.

[42] Li (2010). Membrane targeted horseradish peroxidase as a marker for correlative fluorescence and electron microscopy studies. Front Neural Circuits.

[43] Fulco CP, Munschauer M, Anyoha R, Munson G, Grossman SR, Perez EM (2016). Systematic mapping of functional enhancer-promoter connections with CRISPR interference. Science.

[44] Watt S, Vasquez L, Walter K, Mann AL, Kundu K, Chen L (2021). Genetic perturbation of PU.1 binding and chromatin looping at neutrophil enhancers associates with autoimmune disease. Nat Commun.

[45] Abramov S, Boytsov A, Bykova D, Penzar DD, Yevshin I, Kolmykov SK, et al. Landscape of allele-specific transcription factor binding in the human genome. Nat Commun. 2021. https://adastra.autosome.org/bill-cipher. 10.1038/s41467-021-23007-0 PMC8115691 33980847

[46] Hu H, Lin S, Wang S, Chen X (2020). The role of transcription factor 21 in epicardial cell differentiation and the development of coronary heart disease. Front Cell Dev Biol.

[47] Kallies A, Good-Jacobson KL (2017). Transcription factor T-bet orchestrates lineage development and function in the immune system. Trends Immunol.

[48] Hammal F, de Langen P, Bergon A, Lopez F, Ballester B. ReMap 2022: a database of human, mouse, Drosophila and Arabidopsis regulatory regions from an integrative analysis of DNA-binding sequencing experiments. Nucleic Acids Res. 2022. https://remap-cisreg.github.io/ReMapEnrich/. 10.1093/nar/gkab996 PMC8728178 34751401

[49] Sollis E, Mosaku A, Abid A, Buniello A, Cerezo M, Gil L, et al. The NHGRI-EBI GWAS catalog: knowledgebase and deposition resource. Nucleic Acids Res. 2023. https://www.ebi.ac.uk/gwas/downloads/summary-statistics. 10.1093/nar/gkac1010 PMC9825413 36350656

[50] Kundu K, Tardaguila M, Mann AL, Watt S, Ponstingl H, Vasquez L (2022). Genetic associations at regulatory phenotypes improve fine-mapping of causal variants for 12 immune-mediated diseases. Nat Genet.

[51] Ishigaki K, Sakaue S, Terao C, Luo Y, Sonehara K, Yamaguchi K (2022). Multi-ancestry genome-wide association analyses identify novel genetic mechanisms in rheumatoid arthritis. Nat Genet.

[52] de Lange KM, Moutsianas L, Lee JC, Lamb CA, Luo Y, Kennedy NA (2017). Genome-wide association study implicates immune activation of multiple integrin genes in inflammatory bowel disease. Nat Genet.

[53] Bentham J, Morris DL, Graham DSC, Pinder CL, Tombleson P, Behrens TW (2015). Genetic association analyses implicate aberrant regulation of innate and adaptive immunity genes in the pathogenesis of systemic lupus erythematosus. Nat Genet.

[54] Chiou J, Geusz RJ, Okino M-L, Han JY, Miller M, Melton R (2021). Interpreting type 1 diabetes risk with genetics and single-cell epigenomics. Nature.

[55] Cordell HJ, Han Y, Mells GF, Li Y, Hirschfield GM, Greene CS (2015). International genome-wide meta-analysis identifies new primary biliary cirrhosis risk loci and targetable pathogenic pathways. Nat Commun.

[56] International Multiple Sclerosis Genetics Consortium (2019). Multiple sclerosis genomic map implicates peripheral immune cells and microglia in susceptibility. Science.

[57] Trynka G, Hunt KA, Bockett NA, Romanos J, Mistry V, Szperl A (2011). Dense genotyping identifies and localizes multiple common and rare variant association signals in celiac disease. Nat Genet.

[58] Liu JZ, van Sommeren S, Huang H, Ng SC, Alberts R, Takahashi A (2015). Association analyses identify 38 susceptibility loci for inflammatory bowel disease and highlight shared genetic risk across populations. Nat Genet.

[59] Cortes A, Hadler J, Pointon JP, Robinson PC, Karaderi T, International Genetics of Ankylosing Spondylitis Consortium (IGAS) (2013). Identification of multiple risk variants for ankylosing spondylitis through high-density genotyping of immune-related loci. Nat Genet.

[60] Tsoi LC, Spain SL, Knight J, Ellinghaus E, Stuart PE, Capon F (2012). Identification of 15 new psoriasis susceptibility loci highlights the role of innate immunity. Nat Genet.

[61] Deakin CT, Bowes J, Rider LG, Miller FW, Pachman LM, Sanner H (2022). Association with HLA-DRβ1 position 37 distinguishes juvenile dermatomyositis from adult-onset myositis. Hum Mol Genet.

[62] Giambartolomei C, Vukcevic D, Schadt EE, Franke L, Hingorani AD, Wallace C, et al. Bayesian test for colocalisation between pairs of genetic association studies using summary statistics. PLoS Genet. 2014. https://github.com/chr1swallace/coloc/. 10.1371/journal.pgen.1004383 PMC4022491 24830394

[63] Perdomo-Sabogal A, Nowick K, Piccini I, Sudbrak R, Lehrach H, Yaspo M-L (2016). Human lineage-specific transcriptional regulation through GA-binding protein transcription factor alpha (GABPa). Mol Biol Evol.

[64] Shimokawa T, Ra C (2005). C/EBPalpha functionally and physically interacts with GABP to activate the human myeloid IgA Fc receptor (Fc alphaR, CD89) gene promoter. Blood.

[65] Jeong R, Bulyk ML. Blood cell traits’ GWAS loci colocalization with variation in PU.1 genomic occupancy prioritizes causal noncoding regulatory variants. Cell Genom. 2023;3(7):100327. 10.1016/j.xgen.2023.100327. 10.1016/j.xgen.2023.100327 PMC10363807 37492098

[66] Bossini-Castillo L, Glinos DA, Kunowska N, Golda G, Lamikanra AA, Spitzer M, et al. Immune disease variants modulate gene expression in regulatory CD4+ T cells. Cell Genom. 2022;2. 10.1016/j.xgen.2022.100117. 10.1016/j.xgen.2022.100117 PMC9010307 35591976

[67] Hu C, Li T, Xu Y, Zhang X, Li F, Bai J (2023). Cell Marker 2.0: an updated database of manually curated cell markers in human/mouse and web tools based on scRNA-seq data. Nucleic Acids Res.

[68] Kurki MI, Karjalainen J, Palta P, Sipilä TP, Kristiansson K, Donner KM (2023). Author correction: FinnGen provides genetic insights from a well-phenotyped isolated population. Nature.

[69] Martos L, Oto J, Fernández-Pardo Á, Plana E, Solmoirago MJ, Cana F (2020). Increase of neutrophil activation markers in venous thrombosis-contribution of circulating activated protein C. Int J Mol Sci.

[70] Robbe P, Ridout KE, Vavoulis DV, Dréau H, Kinnersley B, Denny N (2022). Whole-genome sequencing of chronic lymphocytic leukemia identifies subgroups with distinct biological and clinical features. Nat Genet.

[71] Song H, Liu Y, Tan Y, Zhang Y, Jin W, Chen L (2022). Recurrent noncoding somatic and germline WT1 variants converge to disrupt MYB binding in acute promyelocytic leukemia. Blood.

[72] Yang H, Zhang H, Luan Y, Liu T, Yang W, Roberts KG (2022). Noncoding genetic variation in GATA3 increases acute lymphoblastic leukemia risk through local and global changes in chromatin conformation. Nat Genet.

[73] Do P, Beckwith KA, Cheney C, Tran M, Beaver L, Griffin BG (2019). Leukemic B cell CTLA-4 suppresses costimulation of T cells. J Immunol.

[74] Janovská P, Bryja V (2017). Wnt signalling pathways in chronic lymphocytic leukaemia and B-cell lymphomas. Br J Pharmacol.

[75] Lees J, Hay J, Moles MW, Michie AM (2023). The discrete roles of individual FOXO transcription factor family members in B-cell malignancies. Front Immunol.

[76] Herling CD, Coombes KR, Benner A, Bloehdorn J, Barron LL, Abrams ZB (2019). Time-to-progression after front-line fludarabine, cyclophosphamide, and rituximab chemoimmunotherapy for chronic lymphocytic leukaemia: a retrospective, multicohort study. Lancet Oncol.

[77] Plesingerova H, Librova Z, Plevova K, Libra A, Tichy B, Skuhrova Francova H (2017). COBLL1, LPL and ZAP70 expression defines prognostic subgroups of chronic lymphocytic leukemia patients with high accuracy and correlates with IGHV mutational status. Leuk Lymphoma.

[78] Szabo Q, Donjon A, Jerković I, Papadopoulos GL, Cheutin T, Bonev B (2020). Regulation of single-cell genome organization into TADs and chromatin nanodomains. Nat Genet.

[79] Goel VY, Huseyin MK, Hansen AS (2023). Region Capture Micro-C reveals coalescence of enhancers and promoters into nested microcompartments. Nat Genet.

[80] Luppino JM, Park DS, Nguyen SC, Lan Y, Xu Z, Yunker R (2020). Cohesin promotes stochastic domain intermingling to ensure proper regulation of boundary-proximal genes. Nat Genet.

[81] Liu NQ, Magnitov M, Schijns M, van Schaik T, van der Weide RH, Teunissen H, et al. Rapid depletion of CTCF and cohesin proteins reveals dynamic features of chromosome architecture. bioRxiv. 2021:2021.08.27.457977.

[82] Hsieh T-HS, Cattoglio C, Slobodyanyuk E, Hansen AS, Darzacq X, Tjian R (2022). Enhancer-promoter interactions and transcription are largely maintained upon acute loss of CTCF, cohesin, WAPL or YY1. Nat Genet.

[83] Hafner A, Park M, Berger SE, Murphy SE, Nora EP, Boettiger AN (2023). Loop stacking organizes genome folding from TADs to chromosomes. Mol Cell.

[84] Uhlén M, Fagerberg L, Hallström BM, Lindskog C, Oksvold P, Mardinoglu A, et al. Tissue-based map of the human proteome. Science. 2015. https://www.proteinatlas.org/about/download. 10.1126/science.1260419 25613900

[85] Ray-Jones H, Song Z, Haglund A, Artemov P, Rosa M Della, Burden F, et al. Shared and distinct molecular effects of regulatory genetic variants provide insight into mechanisms of distal enhancer-promoter communication. bioRxiv. 2023:2023.08.04.551251.

[86] van Duin L, Krautz R, Rennie S, Andersson R (2023). Transcription factor expression is the main determinant of variability in gene co-activity. Mol Syst Biol.

[87] Beekman R, Chapaprieta V, Russiñol N, Vilarrasa-Blasi R, Verdaguer-Dot N, Martens JHA, et al. The reference epigenome and regulatory chromatin landscape of chronic lymphocytic leukemia. Nat Med. 2018. EGAD00001004046. https://ega-archive.org/datasets/, http://resources.idibaps.org/paper/the-reference-epigenome-and-regulatory-chromatin-landscape-of-chronic-lymphocytic-leukemia. 10.1038/s41591-018-0028-4 PMC6363101 29785028

[88] Bonfield JK, Marshall J, Danecek P, Li H, Ohan V, Whitwham A, et al. HTSlib: C library for reading/writing high-throughput sequencing data. Gigascience. 2021. https://www.htslib.org/. 10.1093/gigascience/giab007 PMC7931820 33594436

[89] Broad Institute. Picard toolkit. 2019. http://broadinstitute.github.io/picard/.

[90] Zhang Y, Liu T, Meyer CA, Eeckhoute J, Johnson DS, Bernstein BE, et al. Model-based Analysis of ChIP-Seq (MACS). Genome Biol. 2008. https://github.com/macs3-project/MACS. 10.1186/gb-2008-9-9-r137 PMC2592715 18798982

[91] Robinson MD, McCarthy DJ, Smyth GK. edgeR: a Bioconductor package for differential expression analysis of digital gene expression data. Bioinformatics. 2010. https://bioconductor.org/packages/release/bioc/html/edgeR.html. 10.1093/bioinformatics/btp616 PMC2796818 19910308

[92] Delaneau O, Ongen H, Brown AA, Fort A, Panousis NI, Dermitzakis ET. A complete tool set for molecular QTL discovery and analysis. Nat Commun. 2017. https://qtltools.github.io/qtltools/. 10.1038/ncomms15452 PMC5454369 28516912

[93] Love MI, Huber W, Anders S. Moderated estimation of fold change and dispersion for RNA-seq data with DESeq2. Genome Biol. 2014. https://bioconductor.org/packages/release/bioc/html/DESeq2.html. 10.1186/s13059-014-0550-8 PMC4302049 25516281

[94] Saelens W, Cannoodt R, Saeys Y (2018). A comprehensive evaluation of module detection methods for gene expression data. Nat Commun.

[95] Quinlan AR, Hall IM. BEDTools: a flexible suite of utilities for comparing genomic features. Bioinformatics. 2010. https://bedtools.readthedocs.io/en/latest/. 10.1093/bioinformatics/btq033 PMC2832824 20110278

[96] Amemiya HM, Kundaje A, Boyle AP. The ENCODE blacklist: identification of problematic regions of the genome. Sci Rep. 2019. https://github.com/Boyle-Lab/Blacklist/blob/master/lists/hg19-blacklist.v2.bed.gz. 10.1038/s41598-019-45839-z PMC6597582 31249361

[97] Abascal F, Acosta R, Addleman NJ, Adrian J, Afzal V, Ai R, et al. Expanded encyclopaedias of DNA elements in the human and mouse genomes. Nature. 2020. https://screen.encodeproject.org/. 10.1038/s41586-020-2493-4 PMC7410828 32728249

[98] Li H, Durbin R. Fast and accurate short read alignment with Burrows-Wheeler transform. Bioinformatics. 2009. https://github.com/lh3/bwa. 10.1093/bioinformatics/btp324 PMC2705234 19451168

[99] Abdennur N, Fudenberg G, Flyamer IM, Galitsyna AA, Goloborodko A, Imakaev M, et al. Pairtools: from sequencing data to chromosome contacts. PLoS Comput Biol. 2024. https://github.com/open2c/pairtools. 10.1371/journal.pcbi.1012164 PMC11164360 38809952

[100] Abdennur N, Mirny LA. Cooler: scalable storage for Hi-C data and other genomically labeled arrays. Bioinformatics. 2020. https://github.com/open2c/cooler. 10.1093/bioinformatics/btz540 PMC8205516 31290943

[101] Puig RR, Boddie P, Khan A, Castro-Mondragon JA, Mathelier A. UniBind: maps of high-confidence direct TF-DNA interactions across nine species. BMC Genomics. 2021. https://bitbucket.org/CBGR/unibind_enrichment/src/master/. 10.1186/s12864-021-07760-6 PMC8236138 34174819

[102] Purcell S, Neale B, Todd-Brown K, Thomas L, Ferreira MAR, Bender D, et al. PLINK: a tool set for whole-genome association and population-based linkage analyses. Am J Hum Genet. 2007. https://www.cog-genomics.org/plink/2.0/. 10.1086/519795 PMC1950838 17701901

[103] Steinhaus R, Robinson PN, Seelow D. FABIAN-variant: predicting the effects of DNA variants on transcription factor binding. Nucleic Acids Res. 2022. https://genecascade.org/fabian/. 10.1093/nar/gkac393 PMC9252790 35639768

[104] Breeze CE, Haugen E, Reynolds A, Teschendorff A, van Dongen J, Lan Q, et al. Integrative analysis of 3604 GWAS reveals multiple novel cell type-specific regulatory associations. Genome Biol. 2022. https://forge2.altiusinstitute.org/. 10.1186/s13059-021-02560-3 PMC8742386 34996498

[105] Fairley S, Lowy-Gallego E, Perry E, Flicek P. The International Genome Sample Resource (IGSR) collection of open human genomic variation resources. Nucleic Acids Res. 2020. https://ftp.1000genomes.ebi.ac.uk/vol1/ftp/release/20130502/. 10.1093/nar/gkz836 PMC6943028 31584097

[106] Harris HL, Gu H, Olshansky M, Wang A, Farabella I, Eliaz Y, et al. Chromatin alternates between A and B compartments at kilobase scale for subgenic organization. Nat Commun. 2023. ENCSR410MDC. https://www.encodeproject.org/. 10.1038/s41467-023-38429-1 PMC10244318 37280210

[107] Boix CA, James BT, Park YP, Meuleman W, Kellis M. Regulatory genomic circuitry of human disease loci by integrative epigenomics. Nature. 2021. https://personal.broadinstitute.org/cboix/epimap/ChromHMM/observed_aux_18_hg19/CALLS/. 10.1038/s41586-020-03145-z PMC7875769 33536621

[108] Sloan CA, Chan ET, Davidson JM, Malladi VS, Strattan JS, Hitz BC, et al. ENCODE data at the ENCODE portal. Nucleic Acids Res. 2016. ENCFF248XJC, ENCFF759XQV, ENCFF429WGS, ENCFF816QSI, ENCFF583IZF, ENCFF749HDD. https://www.encodeproject.org/.

[109] Pushkarev O, van Mierlo G, Kribelbauer JF, Saelens W, Gardeux V, Deplancke B. Chromatin modules. GitHub; 2023. https://github.com/DeplanckeLab/Chromatin_modules. 10.1016/j.tig.2022.11.003 36549923

[110] Pushkarev O, van Mierlo G, Kribelbauer JF, Saelens W, Gardeux V, Deplancke B. Chromatin modules. Zenodo.2023. 10.5281/zenodo.12600811.

