# Supplementary material for: Non-coding variants impact cis-regulatory coordination in a cell type-specific manner
Source: Genome Biol. 2024 Jul 18;25:190. doi: 10.1186/s13059-024-03333-4 (PMC11256678; doi:10.1186/s13059-024-03333-4)
Supplement: Supplementary file 1 — Additional file 1. Supplementary figures (Figs. S1.1–S5.2). [file 13059_2024_3333_MOESM1_ESM.pdf]

Figure S1.1

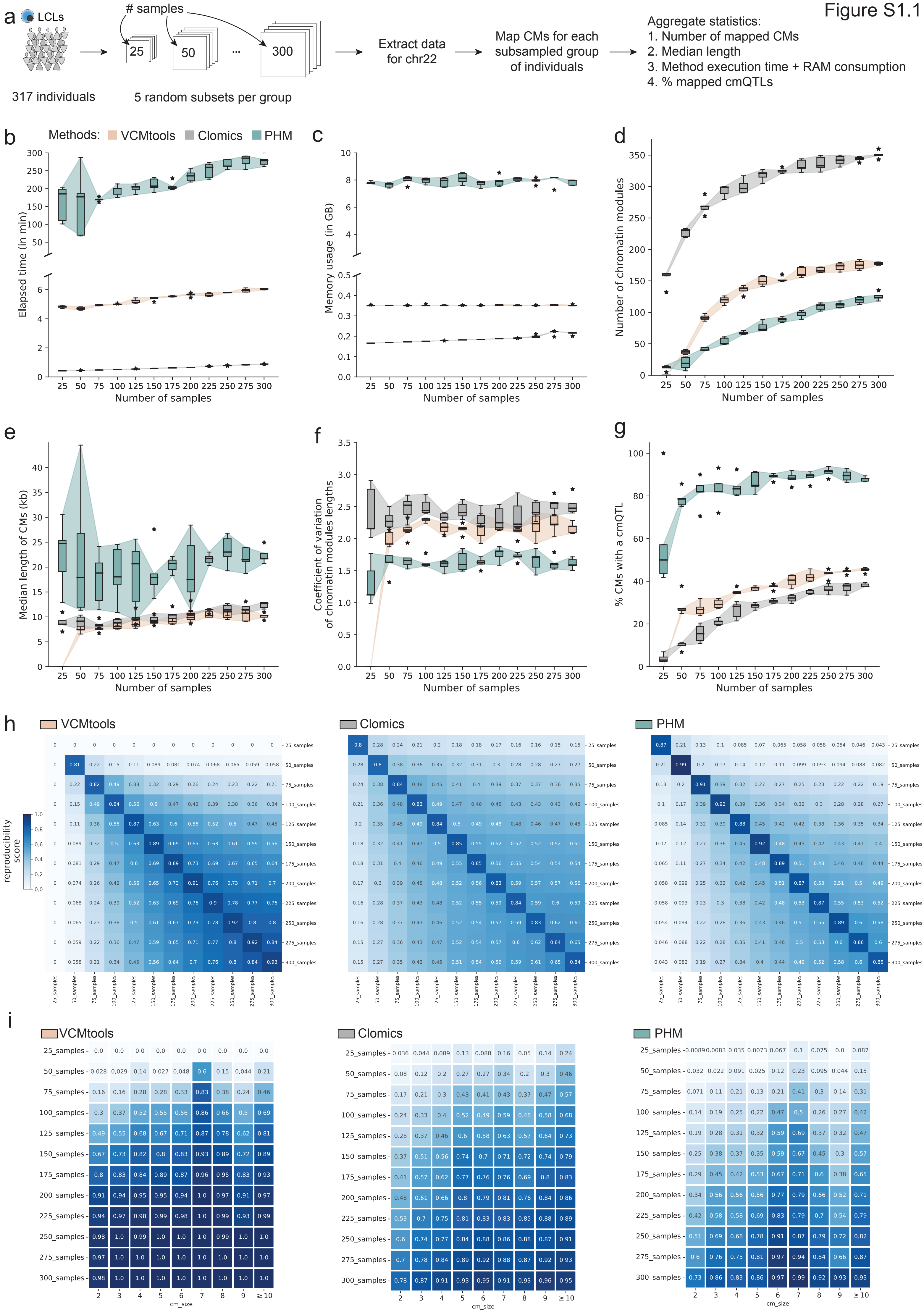

a
LCLs
317 individuals
samples
25
50
300
5 random subsets per group
Extract data for chr22
Map CMs for each subsampled group of individuals
Aggregate statistics:
1. Number of mapped CMs
2. Median length
3. Method execution time + RAM consumption
4. % mapped cmQTLs
b
Methods: VCMtools Clomics PHM
Elapsed time (in min)
Number of samples
c
Memory usage (in GB)
Number of samples
d
Number of chromatin modules
Number of samples
e
Median length of CMs (kb)
Number of samples
f
Coefficient of variation of chromatin modules lengths
Number of samples
g
% CMs with a cmQTL
Number of samples
h
VCMtools
Clomics
PHM
reproducibility score
i
VCMtools
Clomics
PHM
cm_size

Fig S1.1. Performance comparison of CM mapping strategies and general CM characterization. a. Schematic representation of the randomized sample subsampling strategy on chr22 for evaluating the effect of the number of samples on different parameters such as the **b.** elapsed time of CM mapping (excluding data preprocessing time; no parallelization), **c.** maximum RAM occupancy (in GB), **d.** number of mapped CMs, **e.** median CM length, **f.** coefficient of variation of CM length, **g.** percentage of CMs with chromatin module (cm)QTLs. **h.** Heatmaps of average reproducibility scores (F1-based), across five randomized groups per sample size batch, for CMs mapped with (***from left to right***) VCMtools, Clomics and PHM. The values on the diagonal of the heatmaps are not equal to one due to the stacked CMs consisting of the partially overlapping peaks from different histone modifications which results in a decrease of average reproducibility scores. **i**. Average CM peak composition scores across randomized sample size groups w.r.t number of peaks in CMs. The score per CM is defined as the average of CM peak occurrences in 5 randomized calls and is calculated as a number of times the exact CM peak appeared in randomized calls to the total number of calls.

Figure S1.2

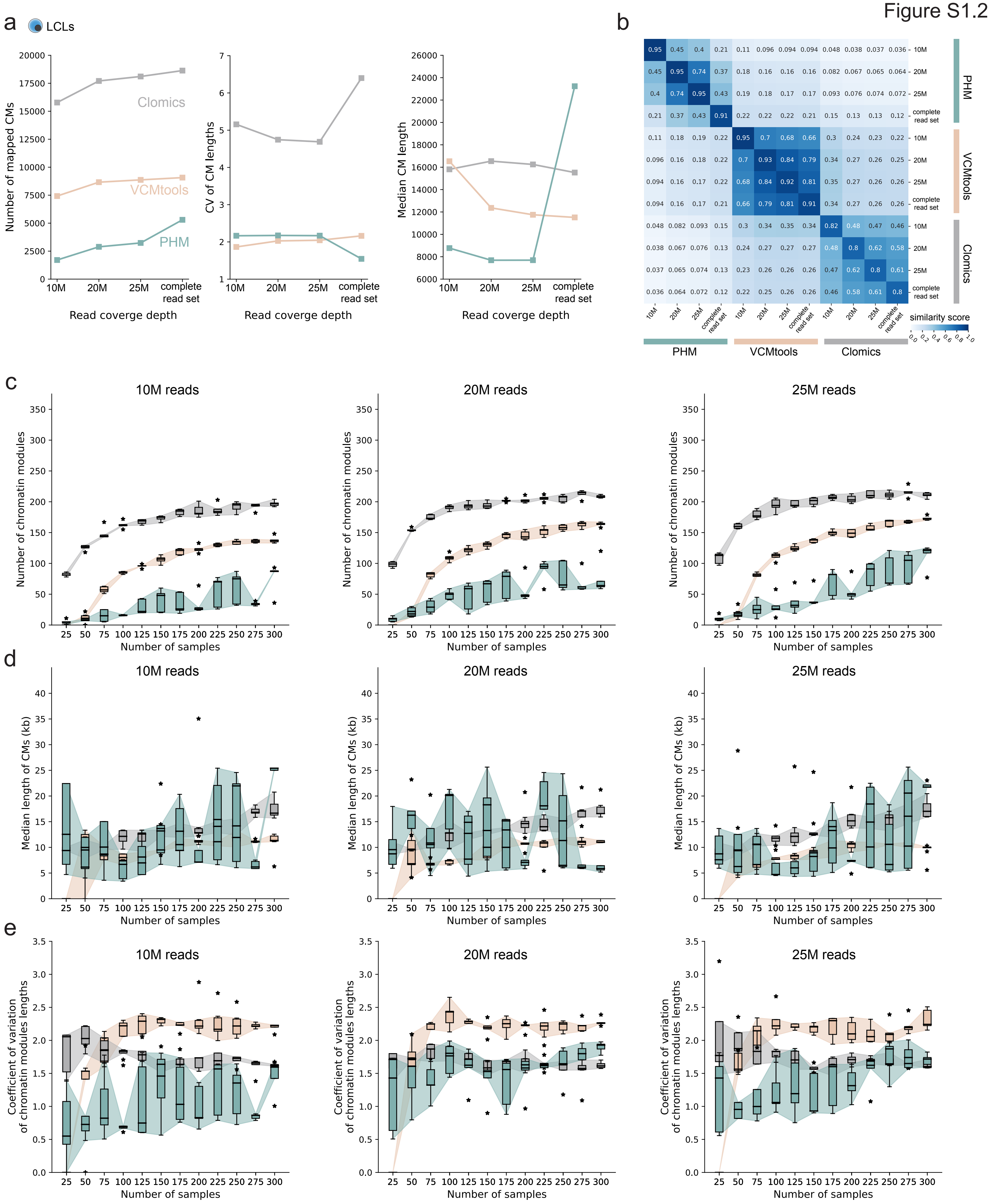

a
LCLs
Number of mapped CMs
Clomics
VCMtools
PHM
CV of CM lengths
Median CM length
10M
20M
25M
complete read set
Read coverge depth
b
similarity score
PHM
VCMtools
Clomics
c
10M reads
20M reads
25M reads
Number of chromatin modules
Number of samples
d
Median length of CMs (kb)
e
Coefficient of variation of chromatin modules lengths

Fig S1.2. The impact of sequencing depth on CM mapping in LCLs. BAM files were downsampled to 25, 20 and 10 million (M) reads. CMs mapped using the downsampled files were compared the set mapped using all reads in LCLs (see **Additional file 4: Table S3** for a summary of the read statistics). **a.** From left to right: Total number of identified CMs, Coefficient of variation (CV) of CM lengths and Median CM lengths. **b**. Heatmap of average reproducibility scores (F1-based) between CMs mapped using different methods and read depths. **c-e**. The effect of read downsampling in combination with sub-sampling on the number of individuals on the total number of identified CMs (**c**), the median length of CMs (**d**) and the coefficient of variation (**e**).

Figure S1.3

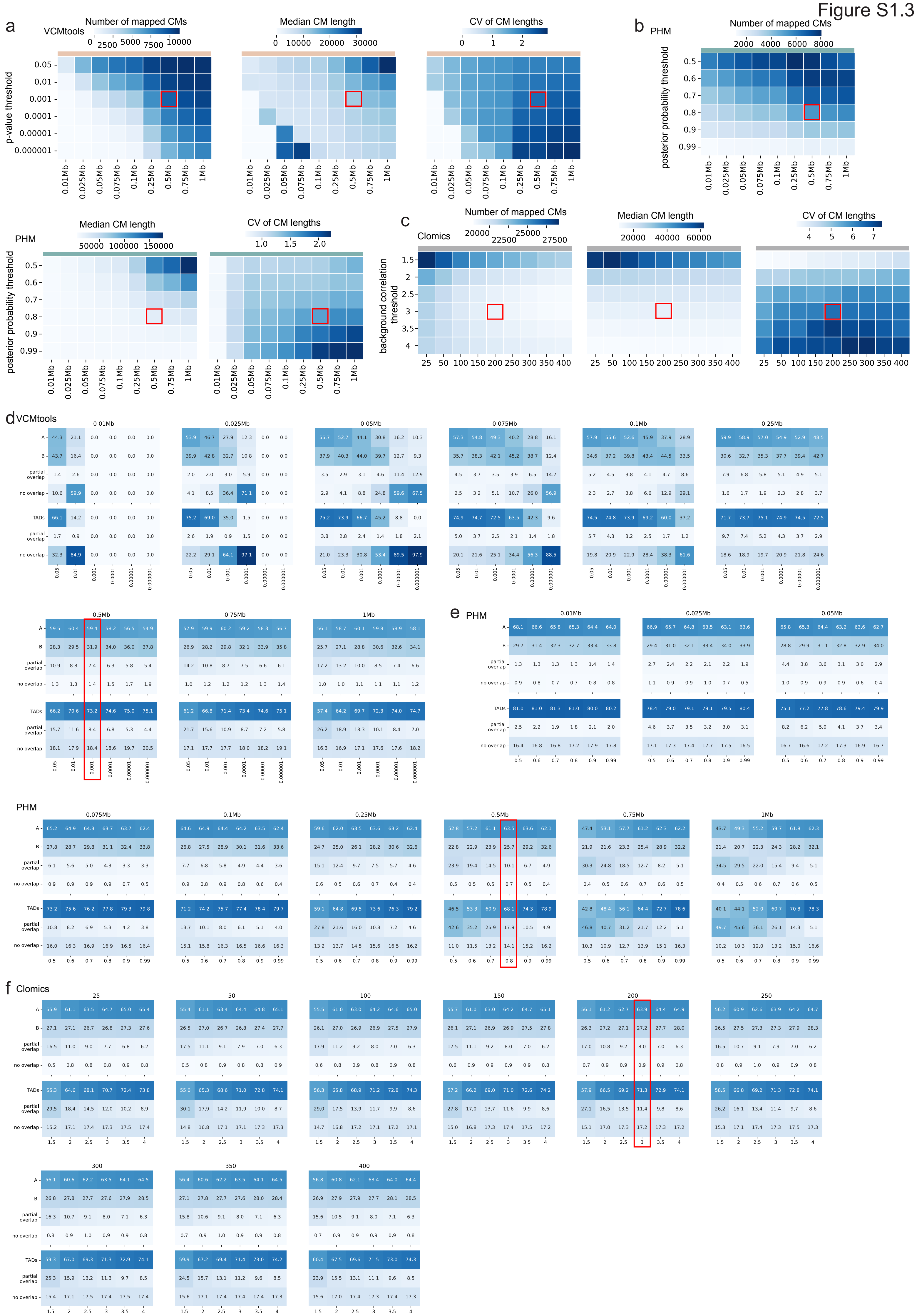
a
VCMtools
Number of mapped CMs
Median CM length
CV of CM lengths
p-value threshold
b
PHM
Number of mapped CMs
posterior probability threshold
PHM
Median CM length
CV of CM lengths
posterior probability threshold
c
Clomics
Number of mapped CMs
Median CM length
CV of CM lengths
background correlation threshold
d
VCMtools
e
PHM
PHM
f
Clomics

Fig S1.3. Impact of method parameters on CM mapping. a. CMs were mapped using VCMtools while varying the parameters for the window size (0.01 - 1Mb) and p-value cut-off (1e-7 – 1e-2, geometric progression with the common ratio=0.1; and a maximum value of 0.05). **b**. CMs were mapped using PHM while varying the parameters for the window size (0.01 - 1Mb) and the posterior probability cut-off (0.5 – 0.9, step=0.1; and a maximum value of 0.99). **c**. CMs were mapped using Clomics while varying the parameters for the window size (25; 50 – 400, step=50 peaks) and p-value cut-off (1.5 - 4, step=0.5). For all parameter combinations and per method we defined the number of mapped CMs, the median length of the CMs and the coefficient of variation of the CM length. For VCMtools and PHM, window sizes indicate the total distance that was considered both left and right for each tested peak. **d-f**. Impact of the parameters on CM overlap with A/B compartments and TADs for VCMtools (**d**), PHM (**e**) and Clomics (**f**). The A, B and TAD categories indicate overlaps with CMs that fully fall into respective genomic regions. In case part of a CM falls outside of the region yet overlaps it by at least one base pair, the respective category is "partial overlap". Otherwise, CM fully falling outside regions of interest are annotated as the "no overlap" group. Across all panels, red rectangles indicate the optimal parameter combination that represents a compromise between the number of CMs versus the distribution, size and overlap with TADs and A/B compartments of these CMs.

Figure S1.4

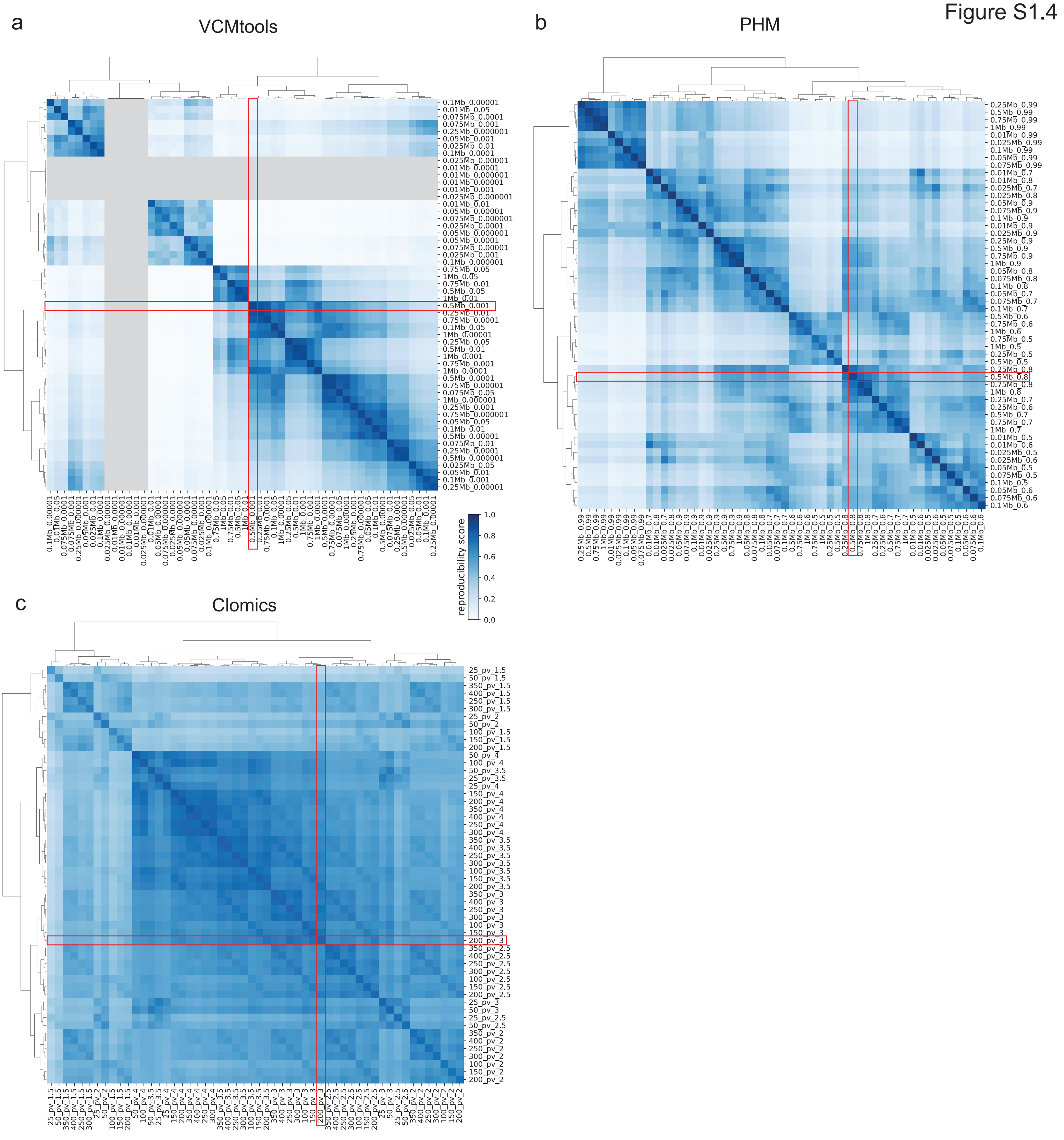
a
VCMtools
0.1Mb_0.00001
0.01Mb_0.05
0.075Mb_0.0001
0.075Mb_0.001
0.25Mb_0.000001
0.05Mb_0.001
0.025Mb_0.01
0.1Mb_0.0001
0.025Mb_0.00001
0.01Mb_0.0001
0.01Mb_0.000001
0.01Mb_0.00001
0.01Mb_0.001
0.025Mb_0.000001
0.01Mb_0.01
0.05Mb_0.0001
0.075Mb_0.000001
0.025Mb_0.0001
0.05Mb_0.000001
0.05Mb_0.0001
0.075Mb_0.00001
0.025Mb_0.001
0.1Mb_0.000001
0.75Mb_0.05
1Mb_0.05
0.75Mb_0.01
0.5Mb_0.05
1Mb_0.01
0.5Mb_0.001
0.25Mb_0.01
0.75Mb_0.0001
0.1Mb_0.05
1Mb_0.00001
0.25Mb_0.05
0.5Mb_0.01
1Mb_0.001
0.75Mb_0.001
1Mb_0.0001
0.5Mb_0.0001
0.75Mb_0.00001
0.075Mb_0.05
1Mb_0.000001
0.25Mb_0.001
0.75Mb_0.000001
0.05Mb_0.05
0.1Mb_0.01
0.5Mb_0.00001
0.075Mb_0.01
0.25Mb_0.0001
0.5Mb_0.000001
0.025Mb_0.05
0.05Mb_0.01
0.1Mb_0.001
0.25Mb_0.00001
reproducibility score
1.0
0.8
0.6
0.4
0.2
0.0
b
PHM
0.25Mb_0.99
0.5Mb_0.99
0.75Mb_0.99
1Mb_0.99
0.01Mb_0.99
0.025Mb_0.99
0.1Mb_0.99
0.05Mb_0.99
0.075Mb_0.99
0.01Mb_0.7
0.01Mb_0.8
0.025Mb_0.7
0.025Mb_0.8
0.05Mb_0.9
0.075Mb_0.9
0.1Mb_0.9
0.01Mb_0.9
0.025Mb_0.9
0.25Mb_0.9
0.5Mb_0.9
0.75Mb_0.9
1Mb_0.9
0.05Mb_0.8
0.075Mb_0.8
0.1Mb_0.8
0.05Mb_0.7
0.075Mb_0.7
0.1Mb_0.7
0.5Mb_0.6
0.75Mb_0.6
1Mb_0.6
0.75Mb_0.5
1Mb_0.5
0.25Mb_0.5
0.5Mb_0.5
0.25Mb_0.8
0.5Mb_0.8
0.75Mb_0.8
1Mb_0.8
0.25Mb_0.7
0.25Mb_0.6
0.5Mb_0.7
0.75Mb_0.7
1Mb_0.7
0.01Mb_0.5
0.01Mb_0.6
0.025Mb_0.5
0.025Mb_0.6
0.05Mb_0.5
0.075Mb_0.5
0.1Mb_0.5
0.05Mb_0.6
0.075Mb_0.6
0.1Mb_0.6
c
Clomics
25_pv_1.5
50_pv_1.5
350_pv_1.5
400_pv_1.5
250_pv_1.5
300_pv_1.5
25_pv_2
50_pv_2
100_pv_1.5
150_pv_1.5
200_pv_1.5
50_pv_4
100_pv_4
50_pv_3.5
25_pv_3.5
25_pv_4
150_pv_4
200_pv_4
350_pv_4
400_pv_4
250_pv_4
300_pv_4
350_pv_3.5
400_pv_3.5
250_pv_3.5
300_pv_3.5
100_pv_3.5
150_pv_3.5
200_pv_3.5
350_pv_3
400_pv_3
250_pv_3
300_pv_3
100_pv_3
150_pv_3
200_pv_3
350_pv_2.5
400_pv_2.5
250_pv_2.5
300_pv_2.5
100_pv_2.5
150_pv_2.5
200_pv_2.5
25_pv_3
50_pv_3
25_pv_2.5
50_pv_2.5
350_pv_2
400_pv_2
250_pv_2
300_pv_2
100_pv_2
150_pv_2
200_pv_2

Fig S1.4. Total overview of the impact of method parameters. The heatmaps represent average reproducibility scores (F1-based) between CMs mapped using VCMtools (**a**), PHM (**b**) and Clomics (**c**) with the different tested parameter combinations. The gray squares in VCMtools result from the fact that those tested parameter combinations didn't identify any CM. Red rectangles indicate the optimal parameter combination defined in Fig S1.3.

Figure S1.5

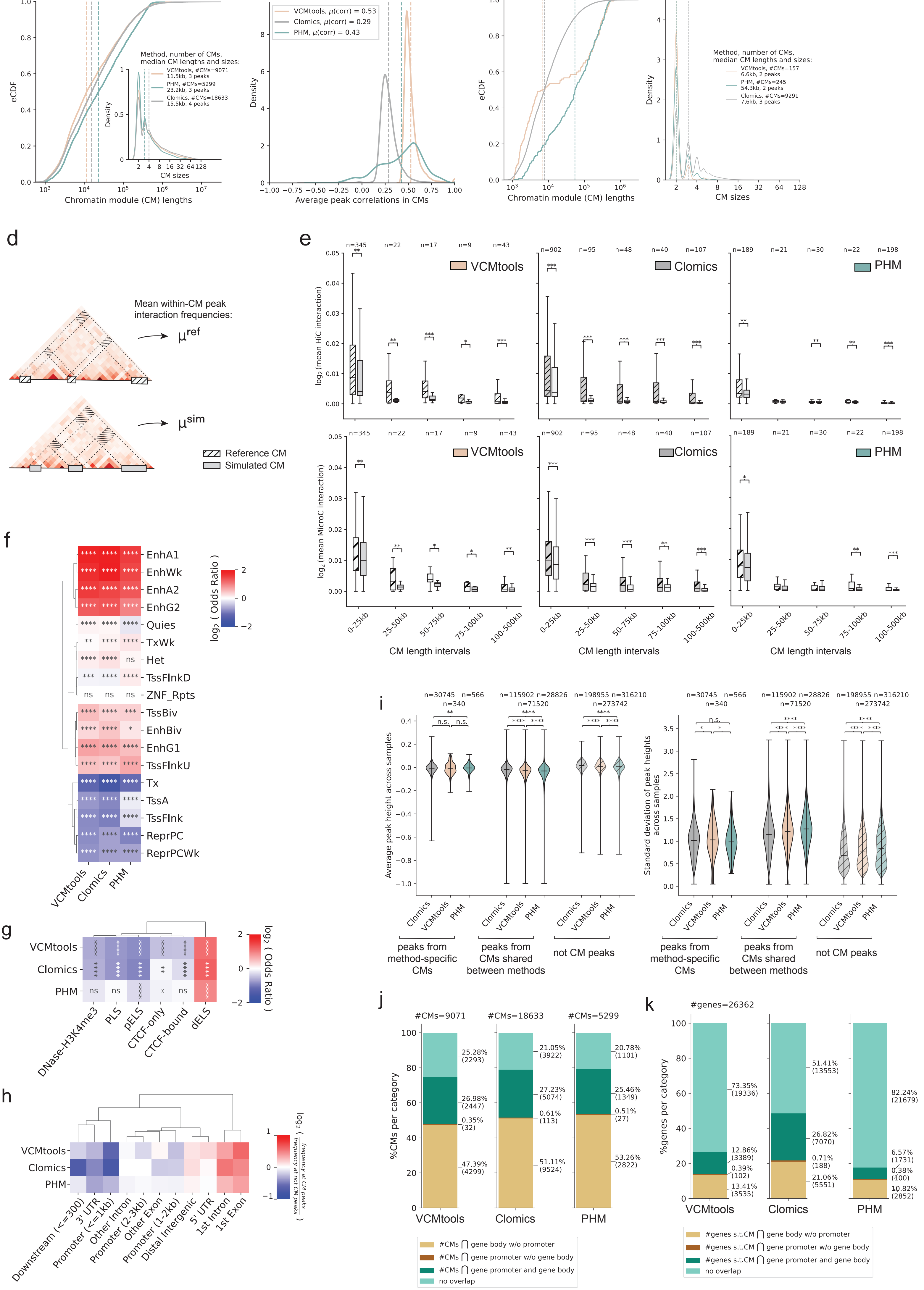

a
Chromatin module (CM) lengths
eCDF
Method, number of CMs, median CM lengths and sizes:
VCMtools, #CMs=9071 11.5kb, 3 peaks
PHM, #CMs=5299 23.2kb, 3 peaks
Clomics, #CMs=18633 15.5kb, 4 peaks
CM sizes
Density
b
VCMtools, μ(corr) = 0.53
Clomics, μ(corr) = 0.29
PHM, μ(corr) = 0.43
Average peak correlations in CMs
c
method-specific CMs
Method, number of CMs, median CM lengths and sizes:
VCMtools, #CMs=157 6.6kb, 2 peaks
PHM, #CMs=245 54.3kb, 2 peaks
Clomics, #CMs=9291 7.6kb, 3 peaks
d
Mean within-CM peak interaction frequencies:
μref
μsim
Reference CM
Simulated CM
e
log2 (mean HiC interaction)
log2 (mean MicroC interaction)
VCMtools
Clomics
PHM
CM length intervals
0-25kb
25-50kb
50-75kb
75-100kb
100-500kb
f
EnhA1
EnhWk
EnhA2
EnhG2
Quies
TxWk
Het
TssFlnkD
ZNF_Rpts
TssBiv
EnhBiv
EnhG1
TssFlnkU
Tx
TssA
TssFlnk
ReprPC
ReprPCWk
log2 (Odds Ratio)
g
DNase-H3K4me3
PLS
pELS
CTCF-only
CTCF-bound
dELS
h
Downstream (<=300)
3' UTR
Promoter (<=1kb)
Other Intron
Promoter (2-3kb)
Other Exon
Promoter (1-2kb)
Distal Intergenic
5' UTR
1st Intron
1st Exon
log2 (frequency at CM peaks / frequency at not CM peaks)
i
Average peak height across samples
Standard deviation of peak heights across samples
peaks from method-specific CMs
peaks from CMs shared between methods
not CM peaks
j
#CMs=9071
#CMs=18633
#CMs=5299
%CMs per category
#CMs ⋂ gene body w/o promoter
#CMs ⋂ gene promoter w/o gene body
#CMs ⋂ gene promoter and gene body
no overlap
k
#genes=26362
%genes per category
#genes s.t.CM ⋂ gene body w/o promoter
#genes s.t.CM ⋂ gene promoter w/o gene body
#genes s.t.CM ⋂ gene promoter and gene body
no overlap

Fig S1.5. CM mapping method comparison with respect to genomic CM localization, 3D interactions, CRE annotations and gene overlaps. a. Empirical Cumulative Density Function (eCDF) of CM lengths and sizes (***inset panels***) across methods. **b.** Distribution of average correlation of peaks within CMs across methods. **c**. eCDF of CM lengths and sizes across methods, only for method-specific CMs. **d.** Schematic illustration of within-CM peak interaction frequency quantification based on Hi-C or Micro-C data for the mapped CMs ("reference CMs") and simulated ones ("simulated CMs"). **e.** ***From left to right***: VCMtools, Clomics, PHM. Quantification of 3D interactions between CM peaks of mapped and simulated CMs with Hi-C (top) or Micro-C (bottom) data at 500bp resolution for CMs split by length. Stars indicate the respective adjusted p-value for the Wilcoxon test. The numbers indicate the number of reference and matched simulated CMs included in each category. **f.** Enrichment of CM peaks versus non-CM peaks in ChromHMM-annotated regions across methods. Color intensity corresponds to the log2 Odds Ratio, stars indicate the respective p-value strength for the Fisher exact test. **g.** Log2 frequency of CM peaks found in either of the genomic categories to the frequency of non-CM peaks found in the same category across methods. **h.** Enrichment of CM peaks versus non-CM peaks in SCREEN annotations of CREs across methods. **i.** Average and standard deviation of the normalized ChIP-seq peak heights across individuals per method and peak category. From left to right: 1) peaks from CMs that were identified exclusively by one method ("method-specific peaks"), 2) peaks from CMs identified by all methods ("peaks from shared CMs") and 3) peaks that were not identified as part of a CM ("not CM peaks"). **j.** Percentages of CMs that cover total, no, or parts of genes, separated per method. **k.** Percentages of genes overlapped by CMs (relative to all coding genes) falling into each category per method.

Figure S1.6

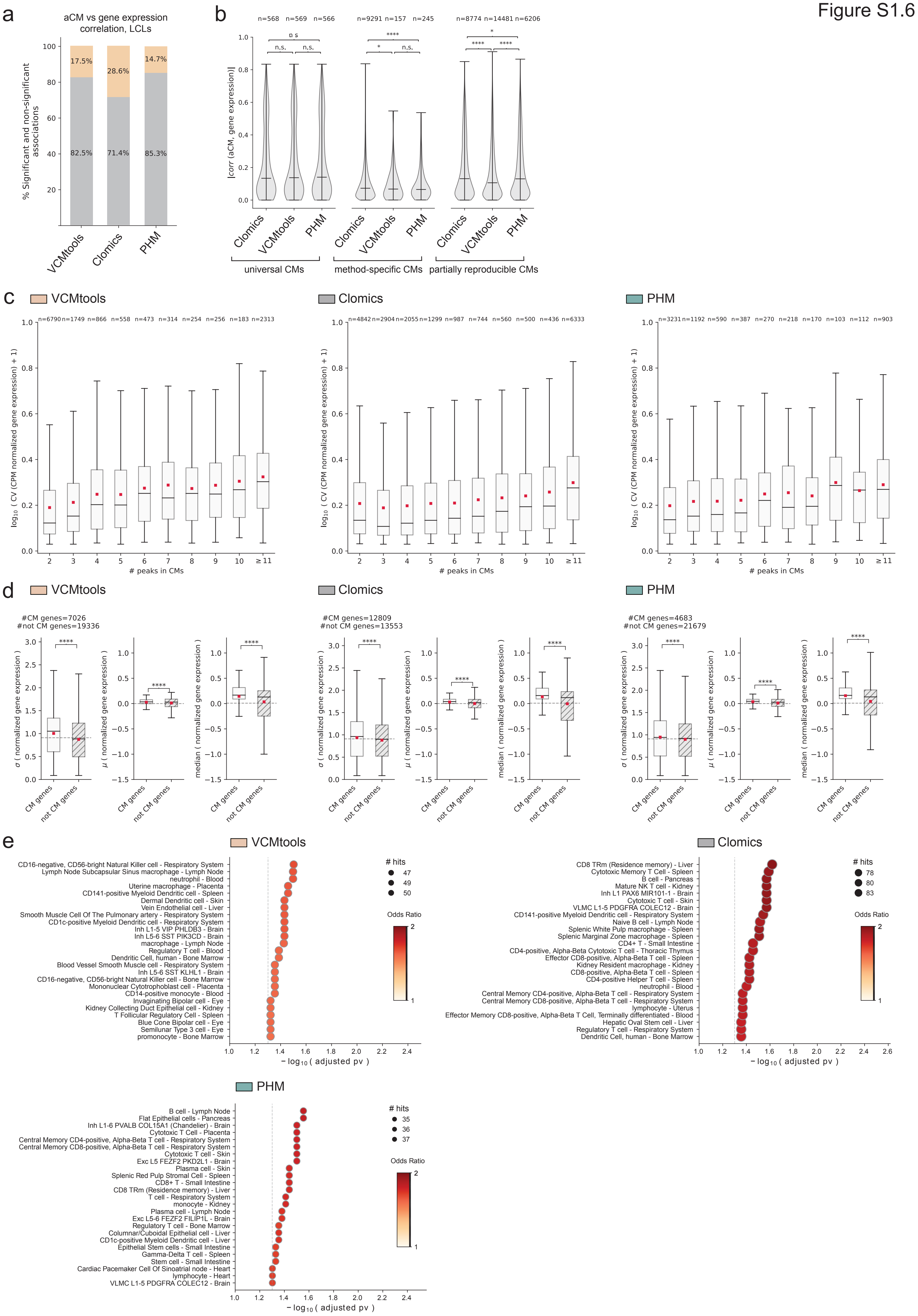
a
aCM vs gene expression correlation, LCLs
% Significant and non-significant associations
17.5%
28.6%
14.7%
82.5%
71.4%
85.3%
VCMtools
Clomics
PHM
b
n=568 n=569 n=566
n=9291 n=157 n=245
n=8774 n=14481 n=6206
|corr (aCM, gene expression)|
universal CMs
method-specific CMs
partially reproducible CMs
c
VCMtools
Clomics
PHM
n=6790 n=1749 n=866 n=558 n=473 n=314 n=254 n=256 n=183 n=2313
n=4842 n=2904 n=2055 n=1299 n=987 n=744 n=560 n=500 n=436 n=6333
n=3231 n=1192 n=590 n=387 n=270 n=218 n=170 n=103 n=112 n=903
log10 (CV (CPM normalized gene expression) + 1)
peaks in CMs
d
#CM genes=7026
#not CM genes=19336
#CM genes=12809
#not CM genes=13553
#CM genes=4683
#not CM genes=21679
σ (normalized gene expression)
μ (normalized gene expression)
median (normalized gene expression)
CM genes
not CM genes
e
VCMtools
CD16-negative, CD56-bright Natural Killer cell - Respiratory System
Lymph Node Subcapsular Sinus macrophage - Lymph Node
neutrophil - Blood
Uterine macrophage - Placenta
CD141-positive Myeloid Dendritic cell - Spleen
Dermal Dendritic cell - Skin
Vein Endothelial cell - Liver
Smooth Muscle Cell Of The Pulmonary artery - Respiratory System
CD1c-positive Myeloid Dendritic cell - Respiratory System
Inh L1-5 VIP PHLDB3 - Brain
Inh L5-6 SST PIK3CD - Brain
macrophage - Lymph Node
Regulatory T cell - Blood
Dendritic Cell, human - Bone Marrow
Blood Vessel Smooth Muscle cell - Respiratory System
Inh L5-6 SST KLHL1 - Brain
CD16-negative, CD56-bright Natural Killer cell - Bone Marrow
Mononuclear Cytotrophoblast cell - Placenta
CD14-positive monocyte - Blood
Invaginating Bipolar cell - Eye
Kidney Collecting Duct Epithelial cell - Kidney
T Follicular Regulatory Cell - Spleen
Blue Cone Bipolar cell - Eye
Semilunar Type 3 cell - Eye
promonocyte - Bone Marrow
hits
47
49
50
Odds Ratio
− log10 (adjusted pv)
Clomics
CD8 TRm (Residence memory) - Liver
Cytotoxic Memory T Cell - Spleen
B cell - Pancreas
Mature NK T cell - Kidney
Inh L1 PAX6 MIR101-1 - Brain
Cytotoxic T cell - Skin
VLMC L1-5 PDGFRA COLEC12 - Brain
CD141-positive Myeloid Dendritic cell - Respiratory System
Naive B cell - Lymph Node
Splenic White Pulp macrophage - Spleen
Splenic Marginal Zone macrophage - Spleen
CD4+ T - Small Intestine
CD4-positive, Alpha-Beta Cytotoxic T cell - Thoracic Thymus
Effector CD8-positive, Alpha-Beta T cell - Spleen
Kidney Resident macrophage - Kidney
CD8-positive, Alpha-Beta T cell - Spleen
CD4-positive Helper T cell - Spleen
neutrophil - Blood
Central Memory CD4-positive, Alpha-Beta T cell - Respiratory System
Central Memory CD8-positive, Alpha-Beta T cell - Respiratory System
lymphocyte - Uterus
Effector Memory CD8-positive, Alpha-Beta T Cell, Terminally differentiated - Blood
Hepatic Oval Stem cell - Liver
Regulatory T cell - Respiratory System
Dendritic Cell, human - Bone Marrow
hits
78
80
83
Odds Ratio
− log10 (adjusted pv)
PHM
B cell - Lymph Node
Flat Epithelial cells - Pancreas
Inh L1-6 PVALB COL15A1 (Chandelier) - Brain
Cytotoxic T Cell - Placenta
Central Memory CD4-positive, Alpha-Beta T cell - Respiratory System
Central Memory CD8-positive, Alpha-Beta T cell - Respiratory System
Cytotoxic T cell - Skin
Exc L5 FEZF2 PKD2L1 - Brain
Plasma cell - Skin
Splenic Red Pulp Stromal Cell - Spleen
CD8+ T - Small Intestine
CD8 TRm (Residence memory) - Liver
T cell - Respiratory System
monocyte - Kidney
Plasma cell - Lymph Node
Exc L5-6 FEZF2 FILIP1L - Brain
Regulatory T cell - Bone Marrow
Columnar/Cuboidal Epithelial cell - Liver
CD1c-positive Myeloid Dendritic cell - Liver
Epithelial Stem cells - Small Intestine
Gamma-Delta T cell - Spleen
Stem cell - Small Intestine
Cardiac Pacemaker Cell Of Sinoatrial node - Heart
lymphocyte - Heart
VLMC L1-5 PDGFRA COLEC12 - Brain
hits
35
36
37
Odds Ratio
− log10 (adjusted pv)

Fig S1.6. CMs mapped with different methods capture relevant genes in a given cell type. a. Significant (orange) and non-significant (gray) associations between the aCM score and expression of genes embedded within CMs. aCM score and gene expression associations with the Benjamini-Hochberg corrected p-value ≤ 0.05 were considered significant. **b.** Correlation of the CM activity (aCM) with the CM-embedded gene expression for CMs that are reproducible using all methods (universal), partially reproducible or method-specific. Brackets with stars indicate the Mann-Whitney U p-value strength for pairwise method comparisons of the absolute correlation values of CM activity (aCM score) and gene expression. P-values are non-significant (ns) for p-value > 0.05, * for 0.01 < p-value ≤ 0.05, ** for 0.001 < p-value ≤ 0.01, *** for 0.0001 < p-value ≤ 0.001, **** p-value ≤ 0.0001. **c.** ***From left to right***: VCMtools, Clomics, PHM. Log10 coefficient of variation (CV) of CPM expression values for genes falling into CMs of different sizes. Red squares indicate the average of the respective distribution. **d.** Standard deviation, average and median of normalized gene expression for genes overlapped by CMs (CM genes) and not overlapped by CMs (other genes). Red squares indicate the average of the respective distribution, dashed gray line indicates the "background average" calculated as an average of the respective statistics across CM and not CM gene groups. Brackets with stars indicate the Mann-Whitney U p-value strength for the tests performed between CM and not CM gene groups for the expression means (**μ**), medians and standard deviations (**σ**). P-values are non-significant (ns) for p-value > 0.05, * for 0.01 < p-value ≤ 0.05, ** for 0.001 < p-value ≤ 0.01, *** for 0.0001 < p-value ≤ 0.001, **** p-value ≤ 0.0001. **e.** ***From left to right***: VCMtools, Clomics, PHM. Gene Ontology terms for genes overlapped by CMs show enrichment for B-cell-specific annotations.

Figure S1.7

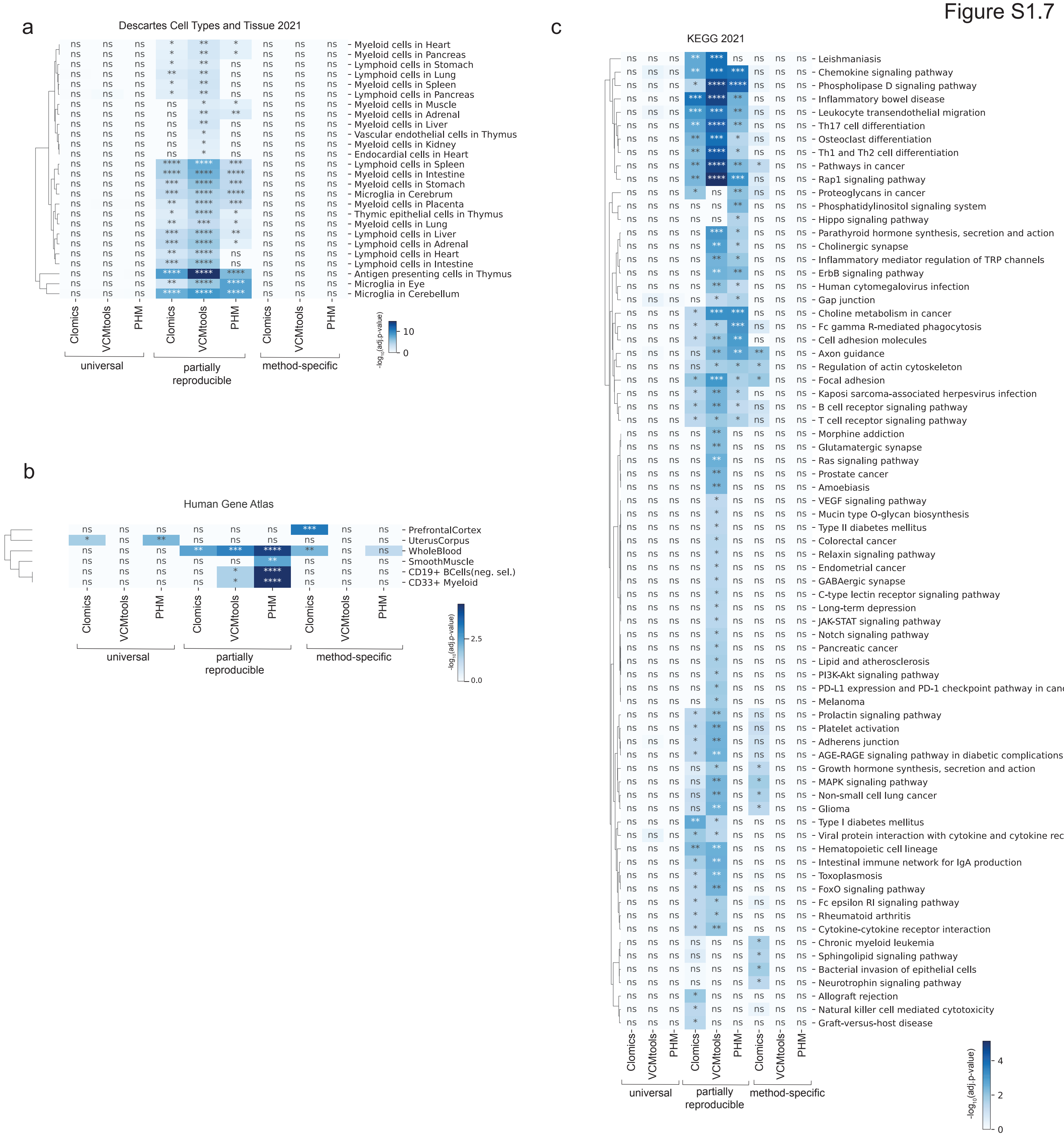
a
Descartes Cell Types and Tissue 2021
Myeloid cells in Heart
Myeloid cells in Pancreas
Lymphoid cells in Stomach
Lymphoid cells in Lung
Myeloid cells in Spleen
Lymphoid cells in Pancreas
Myeloid cells in Muscle
Myeloid cells in Adrenal
Myeloid cells in Liver
Vascular endothelial cells in Thymus
Myeloid cells in Kidney
Endocardial cells in Heart
Lymphoid cells in Spleen
Myeloid cells in Intestine
Myeloid cells in Stomach
Microglia in Cerebrum
Myeloid cells in Placenta
Thymic epithelial cells in Thymus
Myeloid cells in Lung
Lymphoid cells in Liver
Lymphoid cells in Adrenal
Lymphoid cells in Heart
Lymphoid cells in Intestine
Antigen presenting cells in Thymus
Microglia in Eye
Microglia in Cerebellum
Clomics
VCMtools
PHM
universal
partially reproducible
method-specific
-log10(adj.p-value)
10
0
b
Human Gene Atlas
PrefrontalCortex
UterusCorpus
WholeBlood
SmoothMuscle
CD19+ BCells(neg. sel.)
CD33+ Myeloid
Clomics
VCMtools
PHM
universal
partially reproducible
method-specific
-log10(adj.p-value)
2.5
0.0
c
KEGG 2021
Leishmaniasis
Chemokine signaling pathway
Phospholipase D signaling pathway
Inflammatory bowel disease
Leukocyte transendothelial migration
Th17 cell differentiation
Osteoclast differentiation
Th1 and Th2 cell differentiation
Pathways in cancer
Rap1 signaling pathway
Proteoglycans in cancer
Phosphatidylinositol signaling system
Hippo signaling pathway
Parathyroid hormone synthesis, secretion and action
Cholinergic synapse
Inflammatory mediator regulation of TRP channels
ErbB signaling pathway
Human cytomegalovirus infection
Gap junction
Choline metabolism in cancer
Fc gamma R-mediated phagocytosis
Cell adhesion molecules
Axon guidance
Regulation of actin cytoskeleton
Focal adhesion
Kaposi sarcoma-associated herpesvirus infection
B cell receptor signaling pathway
T cell receptor signaling pathway
Morphine addiction
Glutamatergic synapse
Ras signaling pathway
Prostate cancer
Amoebiasis
VEGF signaling pathway
Mucin type O-glycan biosynthesis
Type II diabetes mellitus
Colorectal cancer
Relaxin signaling pathway
Endometrial cancer
GABAergic synapse
C-type lectin receptor signaling pathway
Long-term depression
JAK-STAT signaling pathway
Notch signaling pathway
Pancreatic cancer
Lipid and atherosclerosis
PI3K-Akt signaling pathway
PD-L1 expression and PD-1 checkpoint pathway in canc
Melanoma
Prolactin signaling pathway
Platelet activation
Adherens junction
AGE-RAGE signaling pathway in diabetic complications
Growth hormone synthesis, secretion and action
MAPK signaling pathway
Non-small cell lung cancer
Glioma
Type I diabetes mellitus
Viral protein interaction with cytokine and cytokine rec
Hematopoietic cell lineage
Intestinal immune network for IgA production
Toxoplasmosis
FoxO signaling pathway
Fc epsilon RI signaling pathway
Rheumatoid arthritis
Cytokine-cytokine receptor interaction
Chronic myeloid leukemia
Sphingolipid signaling pathway
Bacterial invasion of epithelial cells
Neurotrophin signaling pathway
Allograft rejection
Natural killer cell mediated cytotoxicity
Graft-versus-host disease
Clomics
VCMtools
PHM
universal
partially reproducible
method-specific
-log10(adj.p-value)
4
2
0

Fig S1.7. **Gene ontology analysis for genes embedded in CMs identified using different methods**. CMs were categorized as being fully or partially reproducible between the methods, or as specific to one of the methods. Genes overlapped by the CMs in each category were analyzed for enrichment in gene ontology terms using the databases Descartes cell types and tissue (**a**), the human gene atlas (**b**) and KEGG pathways (**c**). Color schemes and asterisks indicate -log10 BH-adjusted p-values of the GO-term enrichment. P-value indications are non-significant (ns) for p-value > 0.05, * for 0.01 < p-value ≤ 0.05, ** for 0.001 < p-value ≤ 0.01, *** for 0.0001 < p-value ≤ 0.001, **** p-value ≤ 0.0001.

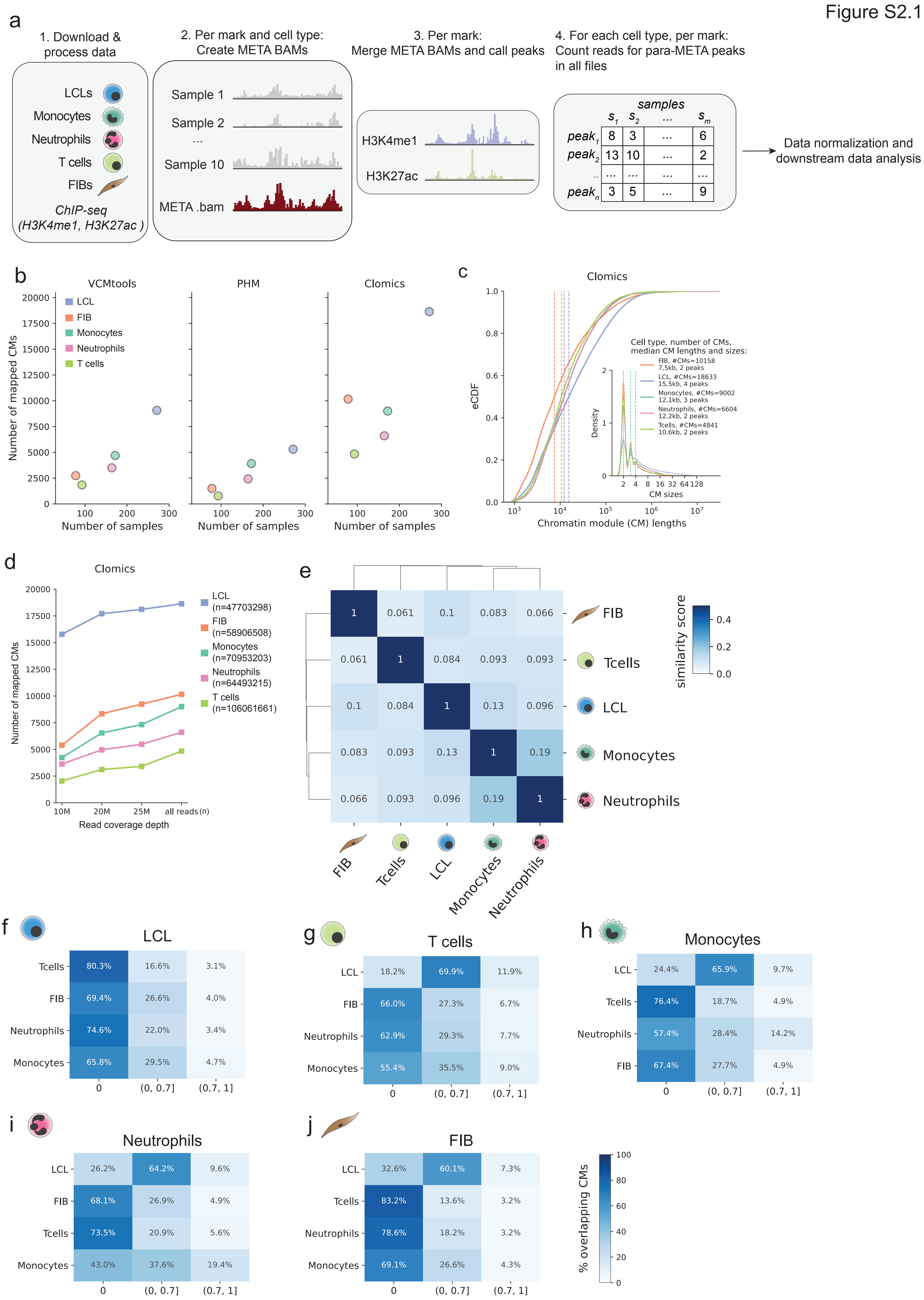

Panel a, step 4 count matrix:

	s_1	s_2	...	s_m
$peak_1$	8	3	...	6
$peak_2$	13	10	...	2
...	...	...	...	...
$peak_n$	3	5	...	9

Panel e (similarity score):

	FIB	Tcells	LCL	Monocytes	Neutrophils
FIB	1	0.061	0.1	0.083	0.066
Tcells	0.061	1	0.084	0.093	0.093
LCL	0.1	0.084	1	0.13	0.096
Monocytes	0.083	0.093	0.13	1	0.19
Neutrophils	0.066	0.093	0.096	0.19	1

f LCL

	0	(0, 0.7]	(0.7, 1]
Tcells	80.3%	16.6%	3.1%
FIB	69.4%	26.6%	4.0%
Neutrophils	74.6%	22.0%	3.4%
Monocytes	65.8%	29.5%	4.7%

g T cells

	0	(0, 0.7]	(0.7, 1]
LCL	18.2%	69.9%	11.9%
FIB	66.0%	27.3%	6.7%
Neutrophils	62.9%	29.3%	7.7%
Monocytes	55.4%	35.5%	9.0%

h Monocytes

	0	(0, 0.7]	(0.7, 1]
LCL	24.4%	65.9%	9.7%
Tcells	76.4%	18.7%	4.9%
Neutrophils	57.4%	28.4%	14.2%
FIB	67.4%	27.7%	4.9%

i Neutrophils

	0	(0, 0.7]	(0.7, 1]
LCL	26.2%	64.2%	9.6%
FIB	68.1%	26.9%	4.9%
Tcells	73.5%	20.9%	5.6%
Monocytes	43.0%	37.6%	19.4%

j FIB

	0	(0, 0.7]	(0.7, 1]
LCL	32.6%	60.1%	7.3%
Tcells	83.2%	13.6%	3.2%
Neutrophils	78.6%	18.2%	3.2%
Monocytes	69.1%	26.6%	4.3%

Fig S2.1. CM mapping and comparison across cell types. a. Schematic representation of the data processing pipeline. First, we downloaded the BAM files for all individuals available for a particular cell type (step 1). Ten BAM files per ChIP-seq histone modification and per cell type were downsampled to 15M reads and merged to create a “meta”-BAM file by stacking up the signal across the files (step 2). For each histone mark, we merged “meta”-BAM files across cell types and used this final merged meta bam file to call peaks (step 3). For each cell type, for each histone mark, we counted reads in the original BAM files based on the peaks obtained from the merged “meta”-BAM files (step 4). Known (based on the available meta data) and unknown covariates (based on principal component analyses) were removed. The resulting count matrices were used for downstream analyses including CM mapping. **b.** Dependency between the number of samples in each cell type and the number of mapped CMs per CM mapping method. **c.** Empirical Cumulative Density Function (eCDF) of CM lengths and sizes mapped using Clomics. **d**. Number of CMs mapped using Clomics in each cell type when using all available reads (indicated by *n* per cell type, see **Additional file 4: Table S3**) or when BAM files were downsampled to 25,20 and 10 million reads. **e.** Average pairwise similarity scores (F1-based) between cell types. **f-j.** Percentage of CMs mapped in a cell type (indicated above each heatmap) that overlap with CMs in other cell types at different similarity score ranges: 0 – no overlap, (0-0.7] – partial overlap, (0.7, 1] – high similarity/identical CMs. One-sided comparison of CM similarities in one cell type versus the others shows 43-83.2% of CMs not being captured in other cell types. When comparing CMs mapped in any cell type to CMs identified in LCLs, 60-70% of CMs falling into the partially overlapping CM group, which can be explained by the extensive number of CMs mapped in LCLs as compared to other cell types.

Figure S2.2

a

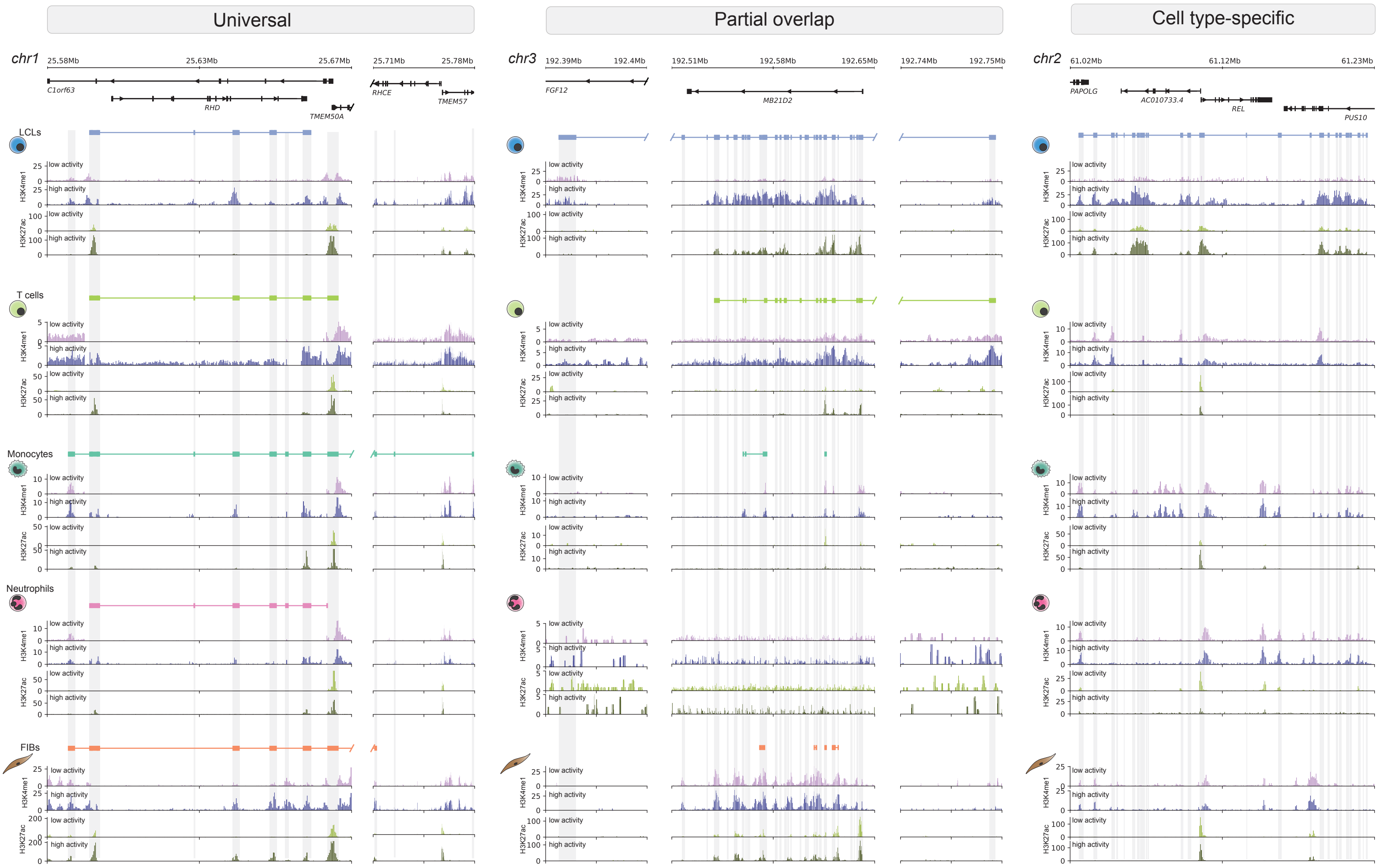

Universal
Partial overlap
Cell type-specific
chr1
25.58Mb
25.63Mb
25.67Mb
25.71Mb
25.78Mb
C1orf63
RHD
TMEM50A
RHCE
TMEM57
chr3
192.39Mb
192.4Mb
192.51Mb
192.58Mb
192.65Mb
192.74Mb
192.75Mb
FGF12
MB21D2
chr2
61.02Mb
61.12Mb
61.23Mb
PAPOLG
AC010733.4
REL
PUS10
LCLs
T cells
Monocytes
Neutrophils
FIBs
H3K4me1
H3K27ac
low activity
high activity

Fig S2.2. Examples of CMs shared between all, a subset of present in a single cell type. a. ***From left to right***: 1. Example of a "universal" CM, here in the *RHD* gene locus where covariable peaks are present in all cell types. 2. Example of a lineage-specific CM in LCLs (blue) and T cells (light green), spanning the *MB21D2* gene. 3. Example of an LCL-specific CM in the *REL* locus. The tracks below correspond to ChIP-seq profiles of H3K27ac and H3K4me1 for two individuals, one with the highest (dark blue (H3K4me1) and dark green (H3K27ac)) and one with the lowest (magenta (H3K4me1) and lime color (H3K27ac)) CM activity. The individuals at the extremes of aCM score were selected on the basis of the largest CM in the locus by calculating pseudo-aCM score in cell types with smaller/absent CMs. Shaded gray areas represent the genomic locations where ChIP-seq peaks were part of the CM in any cell type.

Figure S2.3

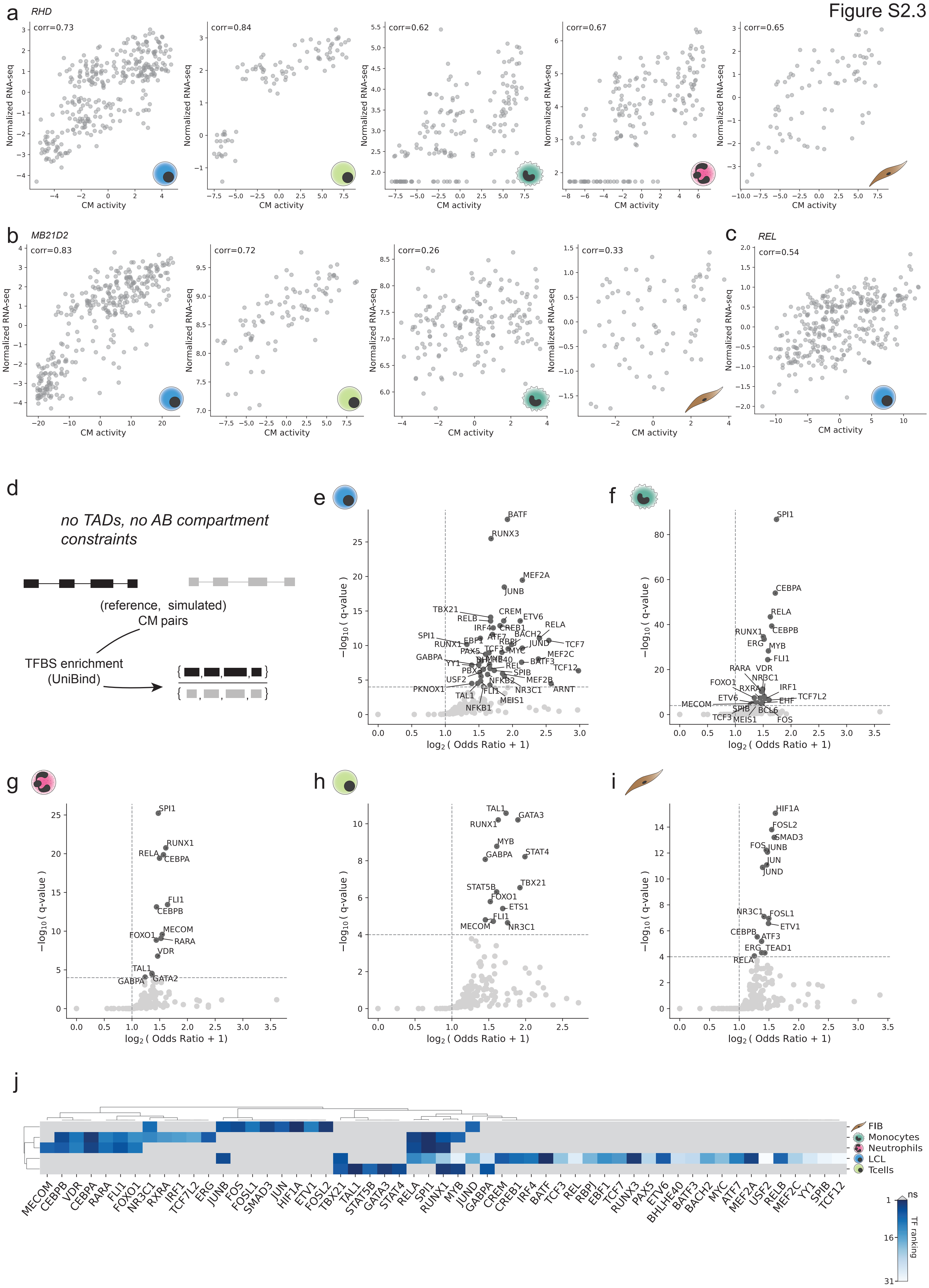
a
RHD
corr=0.73
corr=0.84
corr=0.62
corr=0.67
corr=0.65
Normalized RNA-seq
CM activity
b
MB21D2
corr=0.83
corr=0.72
corr=0.26
corr=0.33
c
REL
corr=0.54
d
no TADs, no AB compartment constraints
(reference, simulated) CM pairs
TFBS enrichment (UniBind)
e
f
g
h
i
−log10 (q-value)
log2 (Odds Ratio + 1)
BATF
RUNX3
MEF2A
JUNB
SPI1
CEBPA
RELA
CEBPB
HIF1A
FOSL2
SMAD3
TAL1
GATA3
STAT4
TBX21
j
FIB
Monocytes
Neutrophils
LCL
Tcells
TF ranking
ns

Fig S2.3. CM activity and TF binding associated with CM regions. a-c. Activity of CMs per cell type (if there is a CM; in the case of several CMs in a locus, the highest correlation between aCM score and RNA-seq data is shown) versus normalized RNA-seq values for *RHD*, *MB21D2* and *REL* genes. **d.** Schematic representation of the differential TFBS enrichment analysis strategy for the paired set of CMs and simulated CMs. Simulated CMs were generated without constraining the genomic reference location to being confined within TADs and A/B compartments. **e-i.** ***From left to right****:* data for LCLs, Monocytes*.* ***Second row from left to right****:* Neutrophils, T cells, Fibroblasts (FIB). TFBS enrichment results per cell type shown as the log2 Odds Ratio versus -log10 q-value of TFBS enrichment within individual cell types when contrasting mapped ("reference") CM peaks vs simulated CM peaks. **j.** Heatmap of TF ranking for TFBSs that passed the q-value threshold (q-value ≤ 1e-04) in at least one of the cell types indicated on the right side. Gray color indicates non-significant hits.

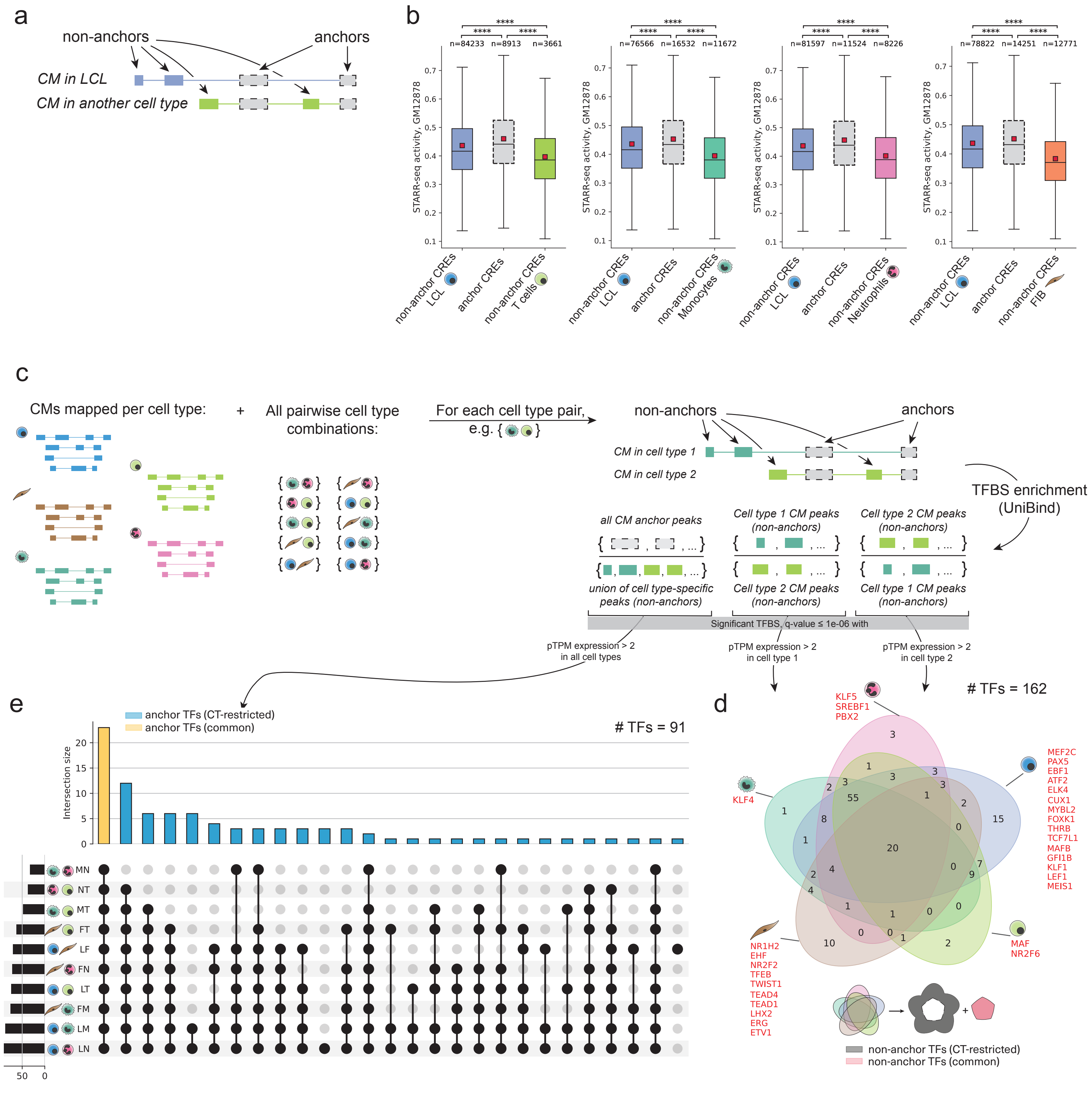
a
non-anchors
anchors
CM in LCL
CM in another cell type
b
STARR-seq activity, GM12878
n=84233 n=8913 n=3661
n=76566 n=16532 n=11672
n=81597 n=11524 n=8226
n=78822 n=14251 n=12771
****
non-anchor CREs LCL
anchor CREs
non-anchor CREs T cells
non-anchor CREs Monocytes
non-anchor CREs Neutrophils
non-anchor CREs FIB
c
CMs mapped per cell type:
+
All pairwise cell type combinations:
For each cell type pair, e.g. { }
non-anchors
anchors
CM in cell type 1
CM in cell type 2
TFBS enrichment (UniBind)
all CM anchor peaks
union of cell type-specific peaks (non-anchors)
Cell type 1 CM peaks (non-anchors)
Cell type 2 CM peaks (non-anchors)
Cell type 2 CM peaks (non-anchors)
Cell type 1 CM peaks (non-anchors)
Significant TFBS, q-value ≤ 1e-06 with
pTPM expression > 2 in all cell types
pTPM expression > 2 in cell type 1
pTPM expression > 2 in cell type 2
d
TFs = 162
KLF5
SREBF1
PBX2
KLF4
MEF2C
PAX5
EBF1
ATF2
ELK4
CUX1
MYBL2
FOXK1
THRB
TCF7L1
MAFB
GFI1B
KLF1
LEF1
MEIS1
NR1H2
EHF
NR2F2
TFEB
TWIST1
TEAD4
TEAD1
LHX2
ERG
ETV1
MAF
NR2F6
non-anchor TFs (CT-restricted)
non-anchor TFs (common)
e
anchor TFs (CT-restricted)
anchor TFs (common)
TFs = 91
Intersection size
MN
NT
MT
FT
LF
FN
LT
FM
LM
LN

Fig S2.4. TFBS- and STARR-seq-based classification based of anchor/non-anchor CM elements. a. Schematic representation of how CM CREs are categorized into groups of anchors (shared) and non-anchor (cell type-restricted) CREs in one comparison between two cell types. **b**. Activity of CM CREs, as measured with STARR-seq in GM12878, for pairwise comparisons of LCLs and all other cell types (***from left to right***: T cells, Monocytes, Neutrophils, FIB). Individual side-wise boxplots per panel correspond to STARR-seq activities at cell type-specific CREs for a given cell type pair, whereas the central boxplot shows activity at anchor CM CREs that are thus shared between the respective cell types. Mann–Whitney U test p-value indications are non-significant (ns) for p-value > 0.05, * for 0.01 < p-value ≤ 0.05, ** for 0.001 < p-value ≤ 0.01, *** for 0.0001 < p-value ≤ 0.001, **** p-value ≤ 0.0001. **c.** Schematic representation of the differential TFBS enrichment analysis strategy for CM peaks across all pairwise cell type comparisons. First, for a given cell type pair (in this example, we focused on the pair of "Monocytes and T cells"), for CM peaks from different cell types overlapping by at least 1bp, we split CM CREs into sets of anchor and non-anchor CREs. Next, we performed differential TFBS enrichment analysis with UniBind for 1) anchor vs non-anchor (union of cell type-specific) CREs, 2) non-anchor CREs in cell type 1 vs non-anchor CREs in cell type 2, and 3) non-anchor CREs in cell type 2 vs non-anchor CREs in cell type 1 (see Methods). For those TFs that passed the q-value threshold and pTPM cutoff, we obtained groups of TFs, defined based on enrichment at anchor and non-anchor CREs in cell type (CT)-restricted (TFBSs enriched in one or a few cell types) and CT-common manner (TFBSs enriched in all cell types). **d.** Venn diagram revealing the overlaps between TFBS enrichments within non-anchor CREs in a cell type-specific manner. TFBS enrichment in non-anchor CM CREs of one cell type vs non-anchor CM CREs in all other cell types. Significantly enriched TFBSs (q-value ≤ 1e-06) obtained through this comparison were additionally filtered based on TF expression (pTPM > 2 in at least one cell type), resulting in a total of 162 TF candidates. TF in red are ordered with respect to decreasing pTPM values. **e.** UpSet plot for TFBS enrichment results when contrasting anchor and non-anchor CREs. The total number of TFs that are considered here is 91.

Figure S2.5

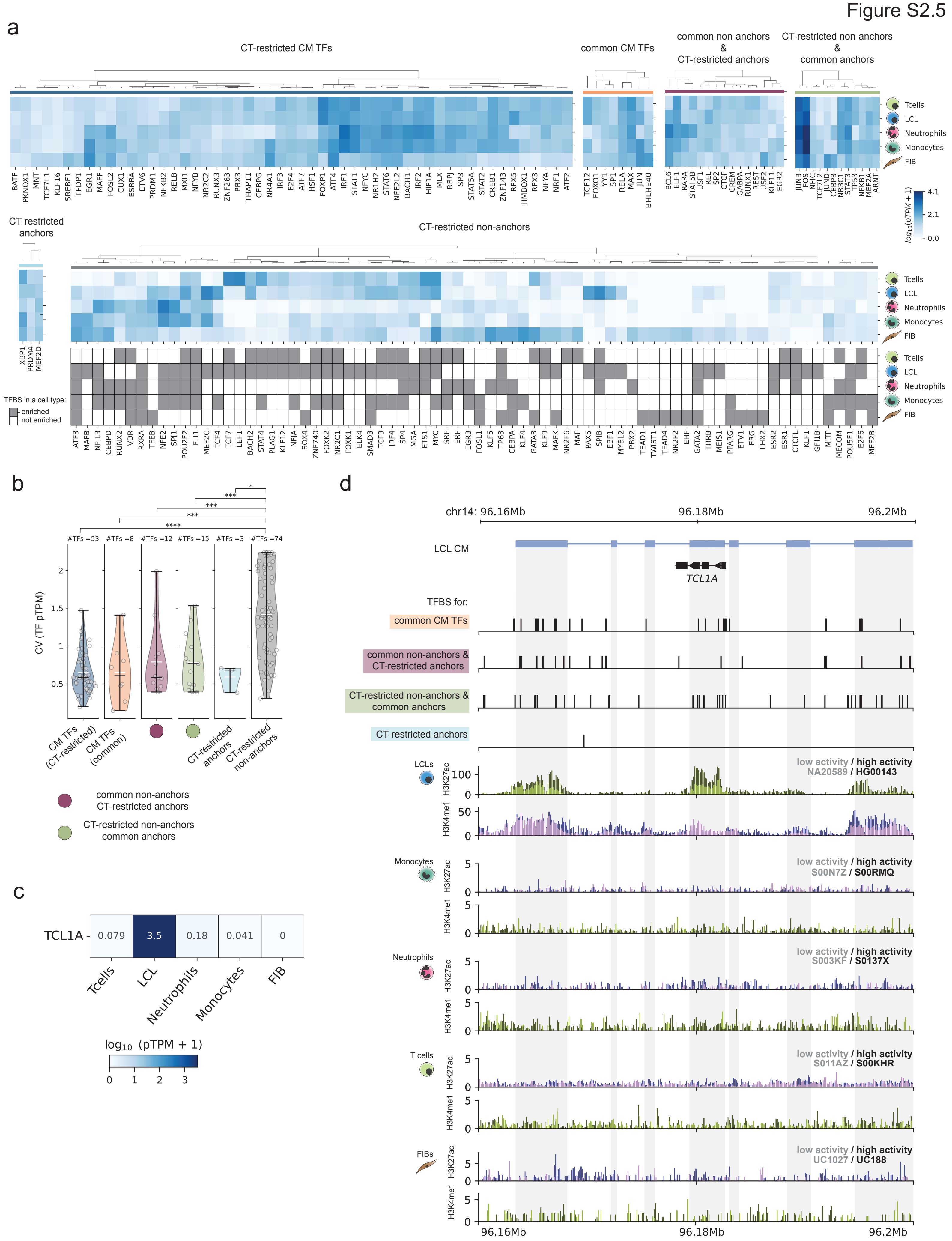
a
CT-restricted CM TFs
common CM TFs
common non-anchors & CT-restricted anchors
CT-restricted non-anchors & common anchors
CT-restricted anchors
CT-restricted non-anchors
Tcells
LCL
Neutrophils
Monocytes
FIB
TFBS in a cell type:
enriched
not enriched
b
CV (TF pTPM)
CM TFs (CT-restricted)
CM TFs (common)
CT-restricted anchors
CT-restricted non-anchors
common non-anchors CT-restricted anchors
CT-restricted non-anchors common anchors
c
TCL1A
0.079
3.5
0.18
0.041
0
log10 (pTPM + 1)
d
chr14: 96.16Mb
96.18Mb
96.2Mb
LCL CM
TCL1A
TFBS for:
common CM TFs
common non-anchors & CT-restricted anchors
CT-restricted non-anchors & common anchors
CT-restricted anchors
LCLs
Monocytes
Neutrophils
T cells
FIBs
H3K27ac
H3K4me1
low activity / high activity
NA20589 / HG00143
S00N7Z / S00RMQ
S003KF / S0137X
S011AZ / S00KHR
UC1027 / UC188

Fig S2.5. Differential enrichment of TF binding sites (TFBS) in a cell type-dependent manner. a. Expression of TFs per cell type and TF category, indicated in *log10 (protein transcripts per million (pTPM) + 1)*. The gray/white heatmap indicates CT-restricted non-anchors TFs, where gray color corresponds to a significantly enriched TFBS on a per cell type basis. **b.** Coefficient of variation of TF expression (pTPM) between cell types for TF categories identified through differential TFBS enrichment analysis. Stars indicate the respective p-value strength for the Mann-Whitney U test with an absence of brackets linking the groups indicating a non-significant difference. **c.** *TCL1A* expression (pTPM) across cell types. **d.** Tracks with black vertical lines indicate TFBSs for TFs falling into defined categories (see **Fig 2** for the respective legend). The bottom tracks correspond to ChIP-seq profiles of H3K27ac and H3K4me1 for two individuals, one with the highest (dark blue (H3K4me1) and dark green (H3K27ac)) and one with the lowest (magenta (H3K4me1) and lime color (H3K27ac)) CM activity in the *TCL1A* gene locus for all cell types. ***From top to bottom***: LCLs, Monocytes, Neutrophils, T cells, FIB. P-value indications are non-significant (ns) for p-value > 0.05, * for 0.01 < p-value ≤ 0.05, ** for 0.001 < p-value ≤ 0.01, *** for 0.0001 < p-value ≤ 0.001, **** p-value ≤ 0.0001.

Figure S3.1

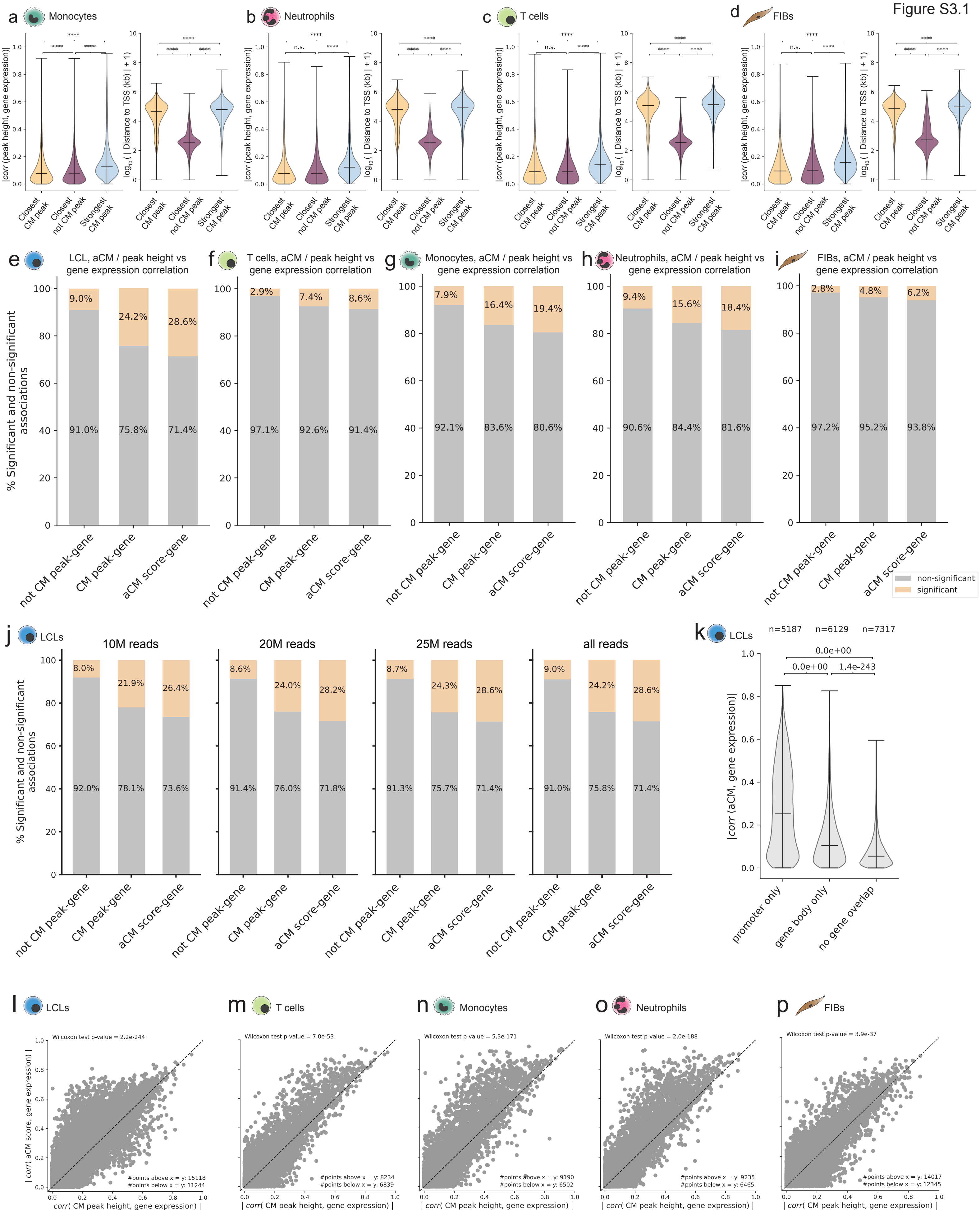

a Monocytes
b Neutrophils
c T cells
d FIBs
|corr (peak height, gene expression)|
log10 (| Distance to TSS (kb) | + 1)
Closest CM peak
Closest not CM peak
Strongest CM peak
n.s.
****
e LCL, aCM / peak height vs gene expression correlation
f T cells, aCM / peak height vs gene expression correlation
g Monocytes, aCM / peak height vs gene expression correlation
h Neutrophils, aCM / peak height vs gene expression correlation
i FIBs, aCM / peak height vs gene expression correlation
% Significant and non-significant associations
not CM peak-gene
CM peak-gene
aCM score-gene
non-significant
significant
9.0% 24.2% 28.6% 91.0% 75.8% 71.4%
2.9% 7.4% 8.6% 97.1% 92.6% 91.4%
7.9% 16.4% 19.4% 92.1% 83.6% 80.6%
9.4% 15.6% 18.4% 90.6% 84.4% 81.6%
2.8% 4.8% 6.2% 97.2% 95.2% 93.8%
j LCLs
10M reads
20M reads
25M reads
all reads
8.0% 21.9% 26.4% 92.0% 78.1% 73.6%
8.6% 24.0% 28.2% 91.4% 76.0% 71.8%
8.7% 24.3% 28.6% 91.3% 75.7% 71.4%
9.0% 24.2% 28.6% 91.0% 75.8% 71.4%
k LCLs
n=5187 n=6129 n=7317
0.0e+00
0.0e+00
1.4e-243
|corr (aCM, gene expression)|
promoter only
gene body only
no gene overlap
l LCLs
m T cells
n Monocytes
o Neutrophils
p FIBs
Wilcoxon test p-value = 2.2e-244
Wilcoxon test p-value = 7.0e-53
Wilcoxon test p-value = 5.3e-171
Wilcoxon test p-value = 2.0e-188
Wilcoxon test p-value = 3.9e-37
| corr(aCM score, gene expression) |
| corr(CM peak height, gene expression) |
#points above x = y: 15118
#points below x = y: 11244
#points above x = y: 8234
#points below x = y: 6839
#points above x = y: 9190
#points below x = y: 6502
#points above x = y: 9235
#points below x = y: 6465
#points above x = y: 14017
#points below x = y: 12345

Fig S3.1. CMs and CM activity explain gene regulatory variation better than individual peaks. a-d. ***From left to right***: Monocytes, Neutrophils, T cells, Fibroblasts (FIB). Violin plots indicating the correlation between each gene expression and the closest CM/not CM peak heights (left in each panel) and distance to the closest peak center from gene transcription start site (TSS) (right in each panel). For each CM that had a peak closest to the gene we also found the best-correlating CM peak, which is indicated as the 'Strongest CM peak' in light blue. Median absolute correlation values per cell type, per category are: Monocytes (Closest CM peak, 0.078; Closest not CM peak, 0.075; Strongest CM peak, 0.126), Neutrophils (Closest CM peak, 0.076; Closest not CM peak, 0.078; Strongest CM peak, 0.122), T cells (Closest CM peak, 0.09; Closest not CM peak, 0.09; Strongest CM peak, 0.144), FIB (Closest CM peak, 0.095; Closest not CM peak, 0.097; Strongest CM peak, 0.158). **e-i.** ***From left to right***: LCLs, T cells, Monocytes, Neutrophils, FIB. Number of significant (light orange) and non-significant (gray) peak-gene associations based on the correlation for three tested groups ***from left to right***: non-CM peak to gene, CM peak to gene, and aCM score to gene**. j.** Number of significant (light orange) and non-significant (gray) peak-gene associations based on the correlation for three tested groups ***from left to right (in each panel)***: non-CM peak to gene, CM peak to gene, and aCM score to gene stratified according to sequencing depth in LCLs. **k.** Violin plot depicting aCM-gene correlation stratified according to whether a gene was embedded in a CM with the promoter, without the promoter but with gene body only, or only being adjacent. P-values were calculated using a Mann-Whitney U test**. l-p.** ***From left to right***: LCLs, T cells, Monocytes, Neutrophils, FIB. Absolute correlation values between height of a peak belonging to a CM and closest gene expression versus absolute correlation values between aCM score and closest gene expression. Wilcoxon test p-value for the differences between the values on the x-axis and the values on the y-axis are indicated in the upper left corner.

Figure S3.2

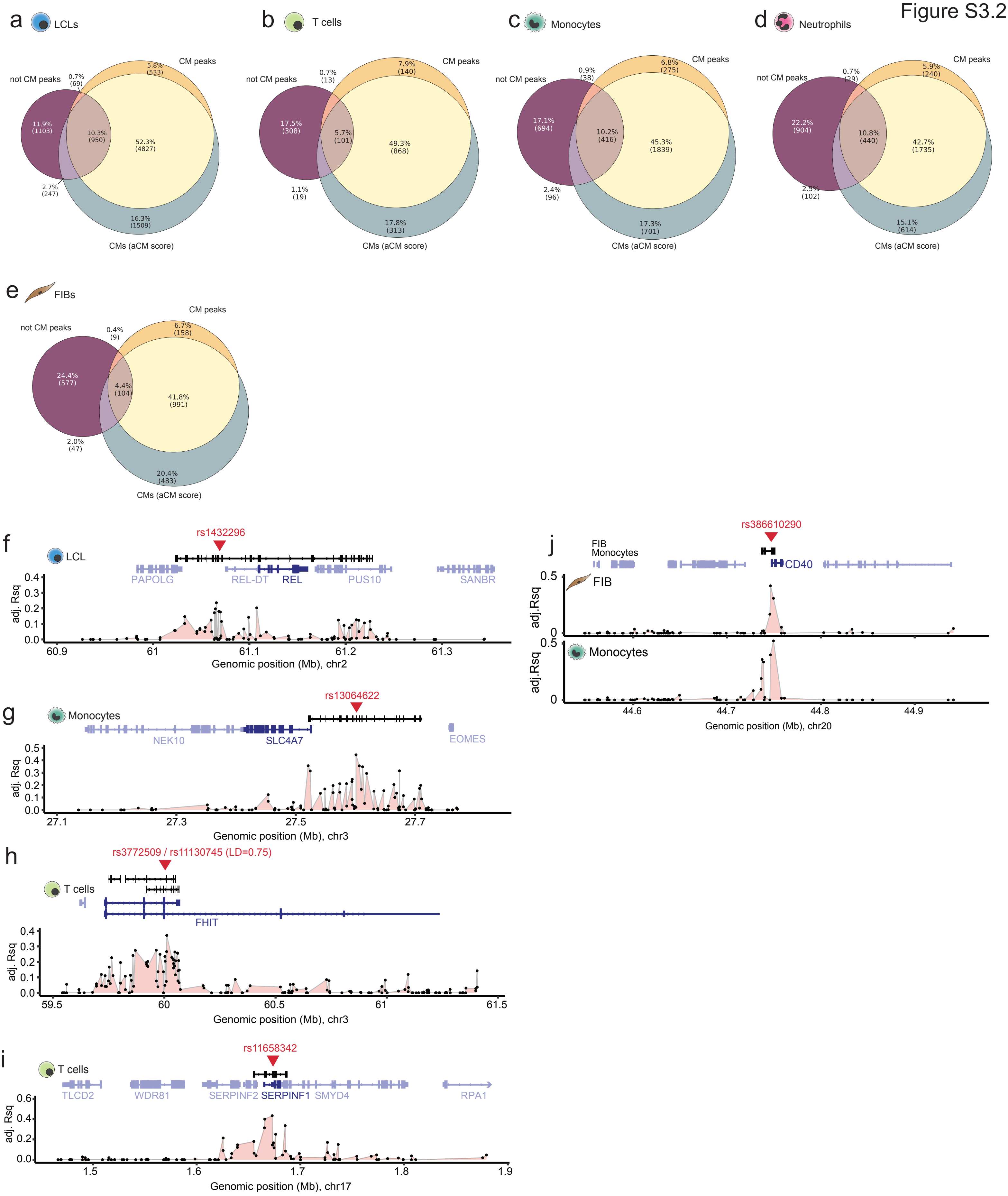

a
LCLs
CM peaks
not CM peaks
0.7% (69)
5.8% (533)
11.9% (1103)
10.3% (950)
52.3% (4827)
2.7% (247)
16.3% (1509)
CMs (aCM score)
b
T cells
CM peaks
not CM peaks
0.7% (13)
7.9% (140)
17.5% (308)
5.7% (101)
49.3% (868)
1.1% (19)
17.8% (313)
CMs (aCM score)
c
Monocytes
CM peaks
not CM peaks
0.9% (38)
6.8% (275)
17.1% (694)
10.2% (416)
45.3% (1839)
2.4% (96)
17.3% (701)
CMs (aCM score)
d
Neutrophils
CM peaks
not CM peaks
0.7% (29)
5.9% (240)
22.2% (904)
10.8% (440)
42.7% (1735)
2.5% (102)
15.1% (614)
CMs (aCM score)
e
FIBs
CM peaks
not CM peaks
0.4% (9)
6.7% (158)
24.4% (577)
4.4% (104)
41.8% (991)
2.0% (47)
20.4% (483)
CMs (aCM score)
f
rs1432296
LCL
PAPOLG
REL-DT
REL
PUS10
SANBR
adj. Rsq
Genomic position (Mb), chr2
g
rs13064622
Monocytes
NEK10
SLC4A7
EOMES
adj. Rsq
Genomic position (Mb), chr3
h
rs3772509 / rs11130745 (LD=0.75)
T cells
FHIT
adj. Rsq
Genomic position (Mb), chr3
i
rs11658342
T cells
TLCD2
WDR81
SERPINF2
SERPINF1
SMYD4
RPA1
adj. Rsq
Genomic position (Mb), chr17
j
rs386610290
FIB
Monocytes
CD40
FIB
adj.Rsq
Monocytes
adj.Rsq
Genomic position (Mb), chr20

Fig S3.2. cmQTLs often impact the strongest CM peak. a-e. *From left to right*: LCL, T cells, Monocytes, Neutrophils, FIB. Venn diagram indicating the percentages of genes falling into different significant peak-gene or aCM-gene association categories. **f-j.** Examples of CMs spanning genes in various cell types having a cmQTL (red triangle). The tracks below CMs and genes show the association strength (adjusted R^2 of the linear regression) between every peak in the locus and expression of the gene highlighted in dark blue. LD values are depicted in R2.

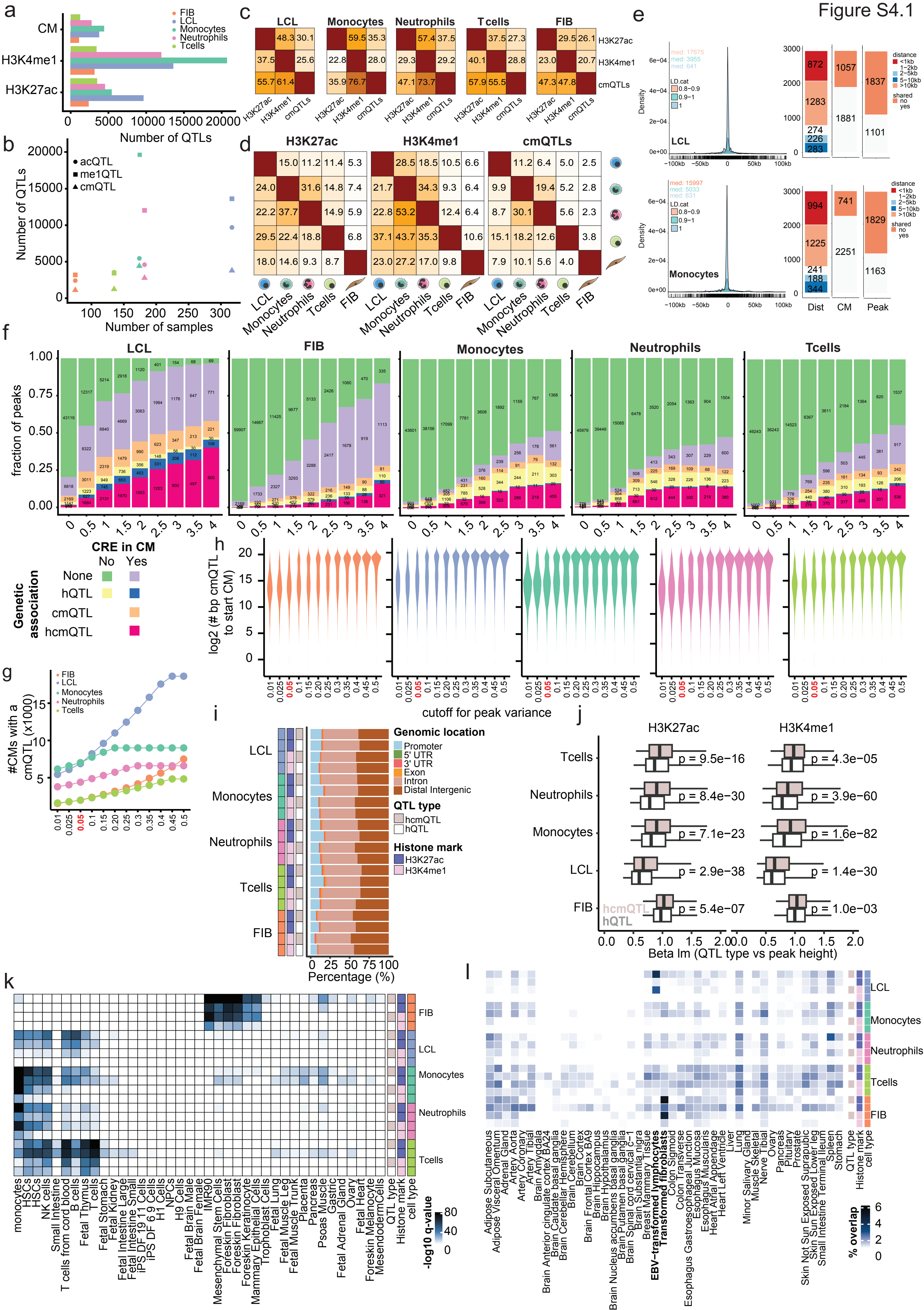
a
CM
H3K4me1
H3K27ac
FIB
LCL
Monocytes
Neutrophils
Tcells
Number of QTLs
b
Number of QTLs
Number of samples
acQTL
me1QTL
cmQTL
c
LCL
Monocytes
Neutrophils
T cells
FIB
H3K27ac
H3K4me1
cmQTLs
d
H3K27ac
H3K4me1
cmQTLs
e
LCL
Monocytes
Density
LD.cat
0.8–0.9
0.9–1
1
distance
<1kb
1–2kb
2–5kb
5–10kb
>10kb
shared
no
yes
Dist
CM
Peak
f
LCL
FIB
Monocytes
Neutrophils
Tcells
fraction of peaks
CRE in CM
No
Yes
None
hQTL
cmQTL
hcmQTL
Genetic association
h
log2 (# bp cmQTL to start CM)
cutoff for peak variance
g
#CMs with a cmQTL (x1000)
i
Genomic location
Promoter
5' UTR
3' UTR
Exon
Intron
Distal Intergenic
QTL type
hcmQTL
hQTL
Histone mark
H3K27ac
H3K4me1
Percentage (%)
j
H3K27ac
H3K4me1
Tcells
p = 9.5e−16
p = 4.3e−05
Neutrophils
p = 8.4e−30
p = 3.9e−60
Monocytes
p = 7.1e−23
p = 1.6e−82
LCL
p = 2.9e−38
p = 1.4e−30
FIB
p = 5.4e−07
p = 1.0e−03
hcmQTL
hQTL
Beta lm (QTL type vs peak height)
k
-log10 q-value
QTL type
Histone mark
cell type
l
% overlap
EBV−transformed lymphocytes
Transformed fibroblasts

Figure S4.1

Fig S4.1. Categorization of cmQTLs and hQTLs. a. Total number of QTLs mapped per category. **b.** Relation between the number of mapped QTLs and number of included individuals. **c.** Percentage of QTLs that are also in LD (R^2 > 0.8) with another type of QTL. **d.** Percentages of QTLs shared (or in LD (R^2 > 0.8)) between cell types. **e**. Density plot: Mapped cmQTLs that locate in peaks and LD variants in peaks, plotted in terms of genomic distance from each other and stratified according to degree of LD. Numbers represent median distance of LD variants to cmQTLs. Bar chart: categorization of distance metrics from all LD variants to the cmQTLs in the density plot, and categorized in whether they locate in the same CRD and the same peak. **f.** H3K27ac peaks were binned by degree of interindividual variance. Numbers on the x-axis represent the number of standard deviations (SD) by which the peaks are variable. For example, 0 is the bin 0-0.5 SD, 0.5 indicates the bin 0.5-1 SD, and 4 represents more than 4 SD variable. Each number on the plot represents the number of peaks in each category. The y-axis represents the cumulative percentage per bin. **g**. Number of CMs with a cmQTL per q-value cut-off. The q-value in red marks the q-value used throughout the manuscript. **h**. Dependency between the q-value used (as in **g**) and the genomic distance of the highest-ranked cmQTL to the start of the CM. From a q-value of 0.15 and higher, the distance of the candidate cmQTL to the CM drastically increases, indicating these are less likely to be causal or acting in *cis*. **i.** Histone QTLs were divided in two groups: histone QTL only (hQTL) and histone + cmQTL (hcmQTL). Genomic locations of the variants in each group are shown. **j.** Boxplots showing the beta values versus height of the peak that the histone QTL affects. P-values were calculated using a Wilcoxon test. **k.** Enrichment of variants in open chromatin regions of each of the indicated cell types. A higher -log10 p-value indicates stronger enrichment. **l.** Percentage of variants overlapping or in LD (R^2 > 0.8) with eQTLs in different GTEx tissues.

Figure S4.2

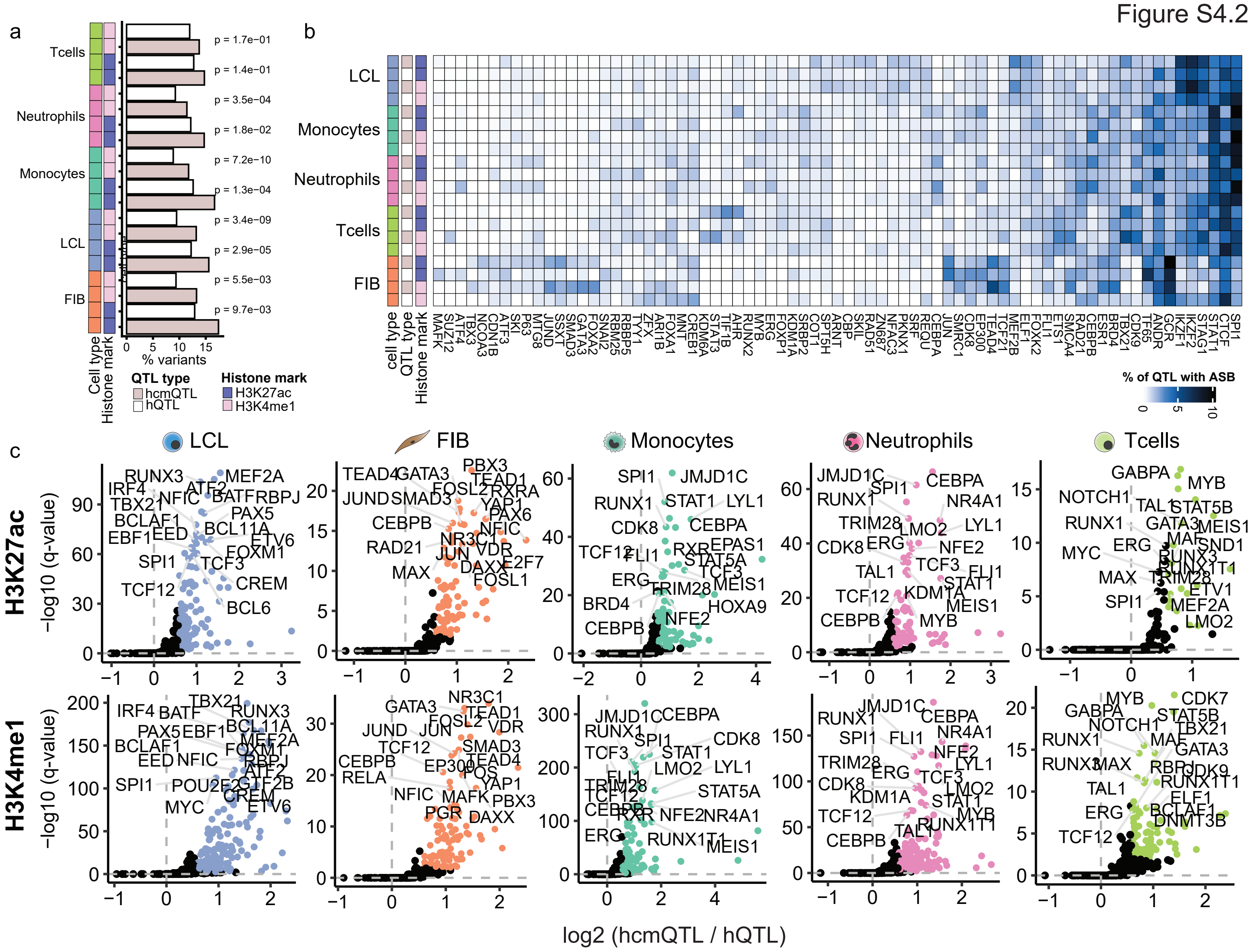
a
Tcells
Neutrophils
Monocytes
LCL
FIB
p = 1.7e−01
p = 1.4e−01
p = 3.5e−04
p = 1.8e−02
p = 7.2e−10
p = 1.3e−04
p = 3.4e−09
p = 2.9e−05
p = 5.5e−03
p = 9.7e−03
% variants
Cell type
Histone mark
QTL type
hcmQTL
hQTL
Histone mark
H3K27ac
H3K4me1
b
LCL
Monocytes
Neutrophils
Tcells
FIB
cell type
QTL type
Histone mark
% of QTL with ASB
c
LCL
FIB
Monocytes
Neutrophils
Tcells
H3K27ac
H3K4me1
−log10 (q-value)
log2 (hcmQTL / hQTL)

Fig S4.2. hcmQTLs enrich for binding of cell type-specific TFs. a. Percentage of genetic variants with at least one allele-specific binding (ASB) event. P-values were calculated using a Fisher's exact test. **b.** Heatmap indicating the percentage of variants that have an ASB associated with each of the indicated TFs. **c.** Scatter plots showing the log2 enrichment of TF binding in 200 bp windows around hcmQTLs compared to hQTLs. Q-values were obtained using a Benjamini-Hochberg correction of p-values associated with the enrichment.

Figure S4.3

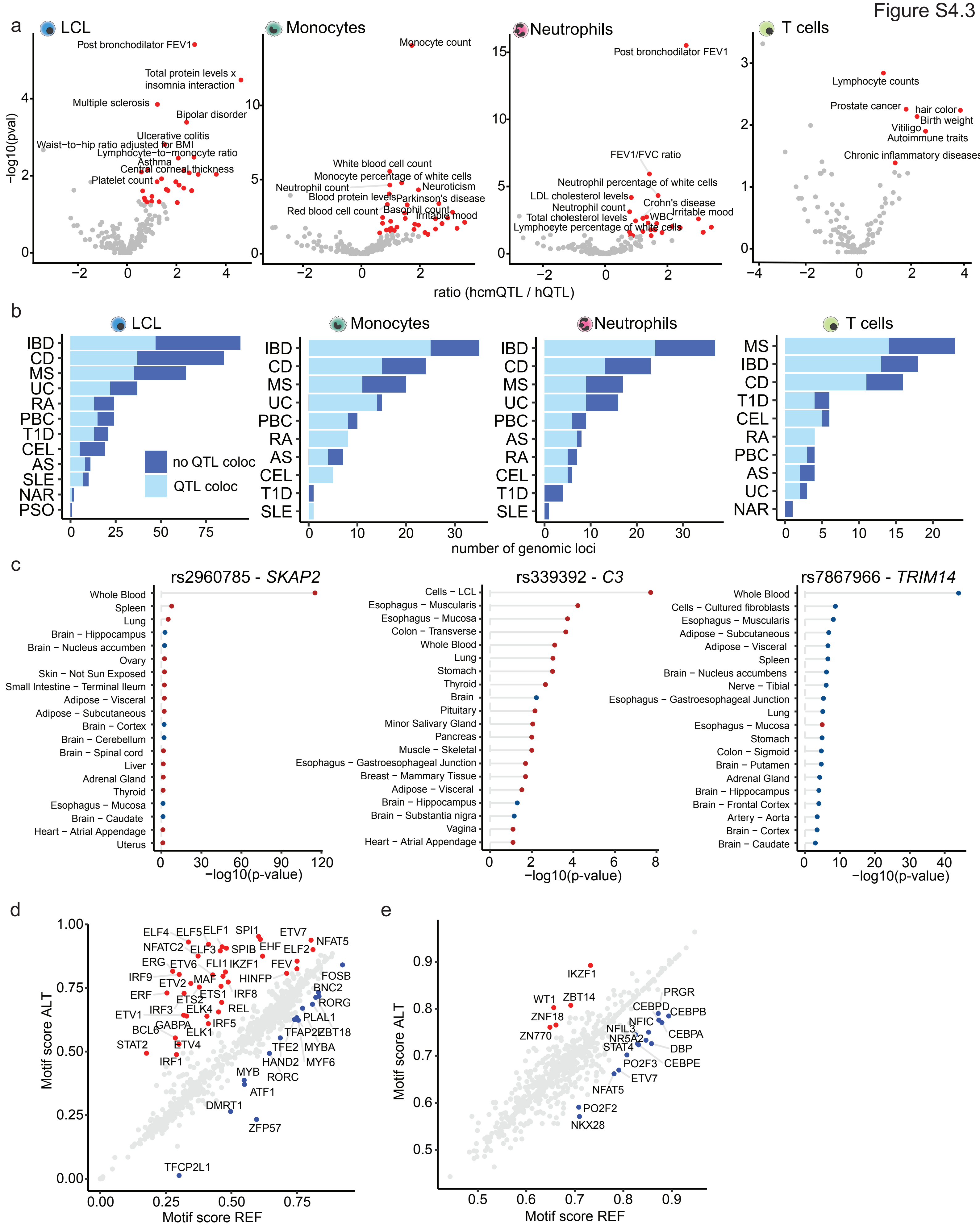

a
LCL
Monocytes
Neutrophils
T cells
−log10(pval)
ratio (hcmQTL / hQTL)
Post bronchodilator FEV1
Total protein levels x insomnia interaction
Multiple sclerosis
Bipolar disorder
Ulcerative colitis
Waist−to−hip ratio adjusted for BMI
Lymphocyte−to−monocyte ratio
Asthma
Central corneal thickness
Platelet count
Monocyte count
White blood cell count
Monocyte percentage of white cells
Neutrophil count
Neuroticism
Blood protein levels
Parkinson's disease
Red blood cell count
Basophil count
Irritable mood
FEV1/FVC ratio
Neutrophil percentage of white cells
LDL cholesterol levels
Crohn's disease
Neutrophil count
Total cholesterol levels
WBC
Lymphocyte percentage of white cells
Lymphocyte counts
Prostate cancer
hair color
Birth weight
Vitiligo
Autoimmune traits
Chronic inflammatory diseases
b
LCL
Monocytes
Neutrophils
T cells
IBD
CD
MS
UC
RA
PBC
T1D
CEL
AS
SLE
NAR
PSO
no QTL coloc
QTL coloc
number of genomic loci
c
rs2960785 - SKAP2
rs339392 - C3
rs7867966 - TRIM14
Whole Blood
Spleen
Lung
Brain − Hippocampus
Brain − Nucleus accumben
Ovary
Skin − Not Sun Exposed
Small Intestine − Terminal Ileum
Adipose − Visceral
Adipose − Subcutaneous
Brain − Cortex
Brain − Cerebellum
Brain − Spinal cord
Liver
Adrenal Gland
Thyroid
Esophagus − Mucosa
Brain − Caudate
Heart − Atrial Appendage
Uterus
Cells − LCL
Esophagus − Muscularis
Colon − Transverse
Stomach
Brain
Pituitary
Minor Salivary Gland
Pancreas
Muscle − Skeletal
Esophagus − Gastroesophageal Junction
Breast − Mammary Tissue
Brain − Substantia nigra
Vagina
Cells − Cultured fibroblasts
Brain − Nucleus accumbens
Nerve − Tibial
Colon − Sigmoid
Brain − Putamen
Brain − Frontal Cortex
Artery − Aorta
−log10(p-value)
d
e
Motif score ALT
Motif score REF
ELF4 ELF5 ELF1 SPI1 ETV7 NFAT5
NFATC2 ELF3 SPIB EHF ELF2
ERG ETV6 FLI1 IKZF1 FEV
IRF9 MAF HINFP FOSB
ETV2 ETS1 IRF8 BNC2
ERF ETS2 RORG
IRF3 ELK4 REL
ETV1 PLAL1
BCL6 GABPA IRF5 TFAP2C ZBT18
ELK1 TFE2 MYBA
STAT2 ETV4 HAND2 MYF6
IRF1 MYB RORC
ATF1
DMRT1
ZFP57
TFCP2L1
IKZF1
PRGR
WT1 ZBT14
CEBPD CEBPB
ZNF18 NFIC
NFIL3 CEBPA
ZN770 NR5A2
STAT4 DBP
PO2F3 CEBPE
ETV7
NFAT5
PO2F2
NKX28

Fig S4.3. Mapping cell type-specific disruption of epigenome organization by GWAS QTLs using CMs. **a.** Log2 observed / expected ratio of hcmQTLs compared to hQTLs for overlap or in LD ($R2 > 0.8$) with GWAS variants. P-values were calculated using a Fisher's exact test. All significant observations ($p < 0.05$) are indicated in red. **b.** Number of autoimmune risk loci with a CM. Color represents whether the candidate cmQTL colocalizes with the GWAS signal at these loci (posterior probability > 0.8 and at least 1 variant that has a p-value of 1e-5 for both GWAS and variant-aCM association). Abbreviations are: spondylitis (AS), Celiac Disease (CEL), Crohn's Disease (CD), Juvenile dermatomyositis (DM), Inflammatory Bowel Disease (IBD), Multiple Sclerosis (MS), primary biliary cirrhosis (PBC), psoriasis (PSO), Rheumatoid Arthritis (RA), Systemic Lupus Erythematosus (SLE), Type 1 Diabetes (T1D) and Ulcerative Colitis (UC). **c.** P-values of associations of rs2960785, rs339392 and rs7867966 with gene expression of *SKAP2*, *C3* and *TRIM14*, respectively, in different sample types derived from the GTEx catalogue. Colours represent whether the minor allele results in increased (red) or decreased (blue) gene expression. **d-e.** Predicted impact of rs339392 (**d**) and rs7867966 (**e**) on the TF motif score (scaled 0 (no match) to 1 (perfect match)).

Figure S4.4

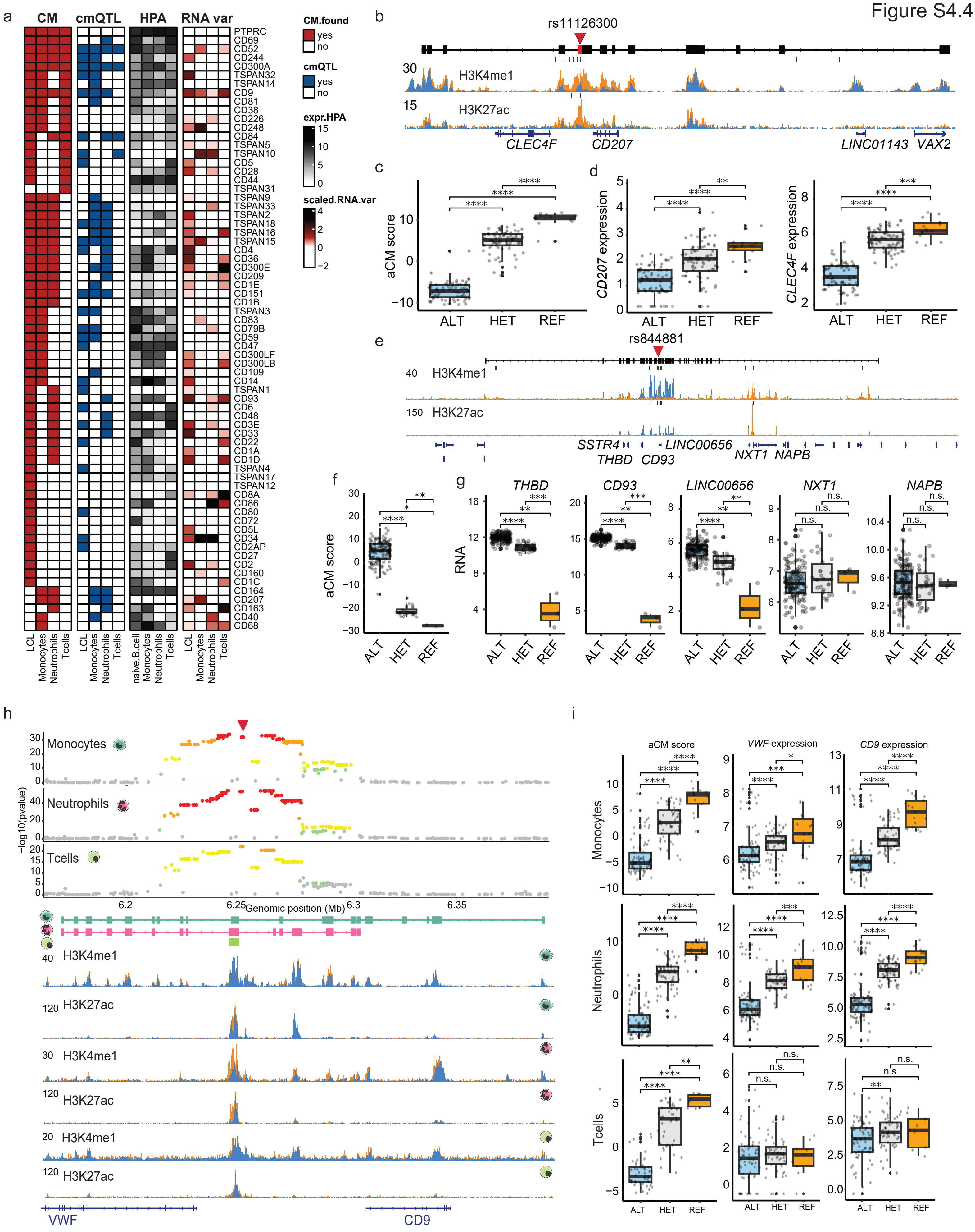
a
CM
cmQTL
HPA
RNA var
CM.found
yes
no
cmQTL
yes
no
expr.HPA
scaled.RNA.var
LCL
Monocytes
Neutrophils
Tcells
naive.B.cell
b
rs11126300
H3K4me1
H3K27ac
CLEC4F
CD207
LINC01143
VAX2
c
aCM score
ALT
HET
REF
d
CD207 expression
CLEC4F expression
e
rs844881
SSTR4
THBD
CD93
LINC00656
NXT1
NAPB
f
g
RNA
h
Monocytes
Neutrophils
Tcells
−log10(pvalue)
Genomic position (Mb)
VWF
CD9
i
aCM score
VWF expression
CD9 expression

Fig S4.4. Genetic variants impacting the epigenome layout around surface receptors through CMs. **a.** Heatmap showing surface markers for which a CM was mapped on the genomic locus (column CM), whether a cmQTL was associated (column cmQTL), the normalized protein levels of these genes in different immune cells based on the Human Protein Atlas (column HPA) and the degree of interindividual variation in gene expression (column RNA var). **b.** Example depicting the *CD207* locus in Monocytes, one example individual per reference (orange) and alternative (blue) genotype. The red triangle indicates the cmQTL and green stripes indicate locations where hQTLs were mapped. **c.** Boxplot of the aCM score stratified on the rs11126300 genotype. **d.** Boxplot of *CD207* and *CLEC4F* expression stratified on the rs11126300 genotype. **e.** Example depicting the *CD93* locus in Monocytes, with one example individual per reference (orange) and alternative (blue) genotype. The red triangle indicates the cmQTL and green stripes indicate locations where hQTLs were mapped. **f.** Boxplot of the aCM score stratified on the rs884881 genotype. **g.** Boxplot of gene expression stratified on the rs884881 genotype. Note that expression of *THBD*, *CD93* and *LINC00656*, which are embedded in the CM, follow the aCM pattern, while the genes *NXT1* and *NAPB* that are not embedded in the CM are similarly expressed between rs884881 genotypes. **h.** Example depicting the *VWF* – *CD9* locus in Monocytes, Neutrophils and T cells. The mapped CM in T cells is small, whereas the CM is extended and covers also putative CREs in *VWF* and *CD9* in Monocytes and Neutrophils. While the same variants seem associated with the shared CM region, there is only an impact on gene expression in Monocytes and Neutrophils. One representative individual for the ALT genotype (blue) and the REF genotype (orange) is shown. Note that all ALT and REF tracks originate from cells from the same individual. **i.** Boxplot of the aCM score and gene expression stratified on the genotype of the shared top variant (indicated by the red triangle in (**h**)). P-value indications are non-significant (ns) for p-value > 0.05, * for 0.01 < p-value ≤ 0.05, ** for 0.001 < p-value ≤ 0.01, *** for 0.0001 < p-value ≤ 0.001, **** p-value ≤ 0.0001.

Figure S5.1

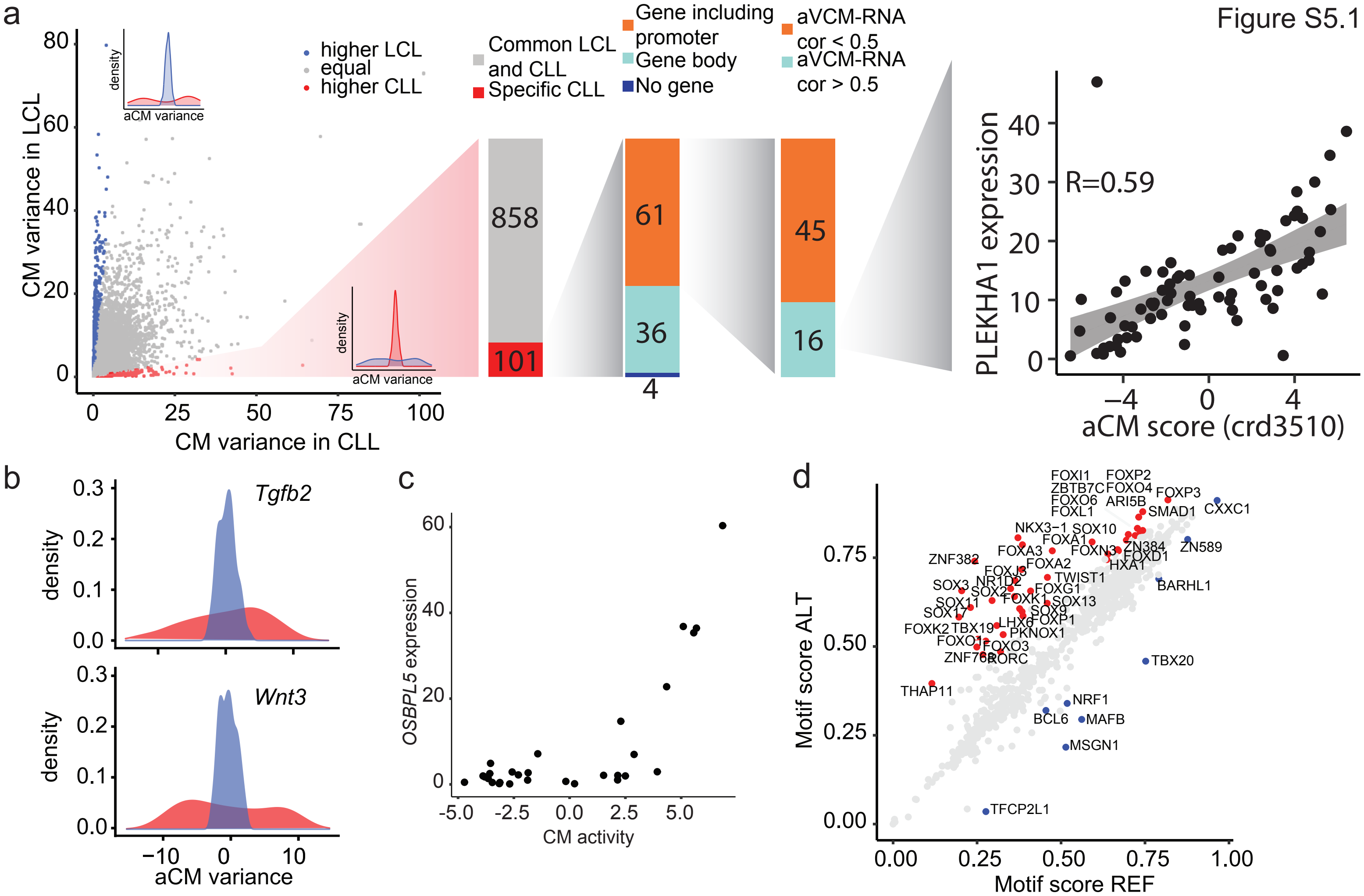

a
higher LCL
equal
higher CLL
Common LCL and CLL
Specific CLL
Gene including promoter
Gene body
No gene
aVCM-RNA cor < 0.5
aVCM-RNA cor > 0.5
CM variance in LCL
CM variance in CLL
aCM variance
density
858
101
61
36
4
45
16
R=0.59
PLEKHA1 expression
aCM score (crd3510)
b
Tgfb2
Wnt3
density
aCM variance
c
OSBPL5 expression
CM activity
d
Motif score ALT
Motif score REF
FOXI1
FOXP2
ZBTB7C
FOXO4
FOXP3
FOXO6
ARI5B
SMAD1
CXXC1
FOXL1
NKX3-1
SOX10
FOXA1
ZN589
FOXA3
FOXN3
ZN384
FOXD1
ZNF382
FOXA2
HXA1
FOXJ3
TWIST1
BARHL1
SOX3
NR1D2
SOX2
FOXG1
SOX11
FOXK1
SOX13
SOX17
SOX9
FOXP1
FOXK2
TBX19
LHX6
PKNOX1
FOXO1
FOXO3
ZNF768
RORC
TBX20
THAP11
NRF1
BCL6
MAFB
MSGN1
TFCP2L1

Fig S5.1. Distinct CMs are formed in CLL and LCL. a. Stringent filtering on the interindividual variance of genomic loci harbouring CMs in either CLL or LCL. Loci that were at least 9 times more variable in either cell type were considered differential, and consequently filtered further based on whether the CM was specifically found in CLL only, if the promoter was included and if the CM activity correlated with expression of the embedded genes. **b.** Example of two regions that show higher interindividual variation in CLL. **c**. Correlation between aCM activity and *OSBPL5* expression. Each dot represents one individual. **d**. Predicted impact of rs895555 on the TF motif score (scaled 0 (no match) to 1 (perfect match)).

Figure S5.2

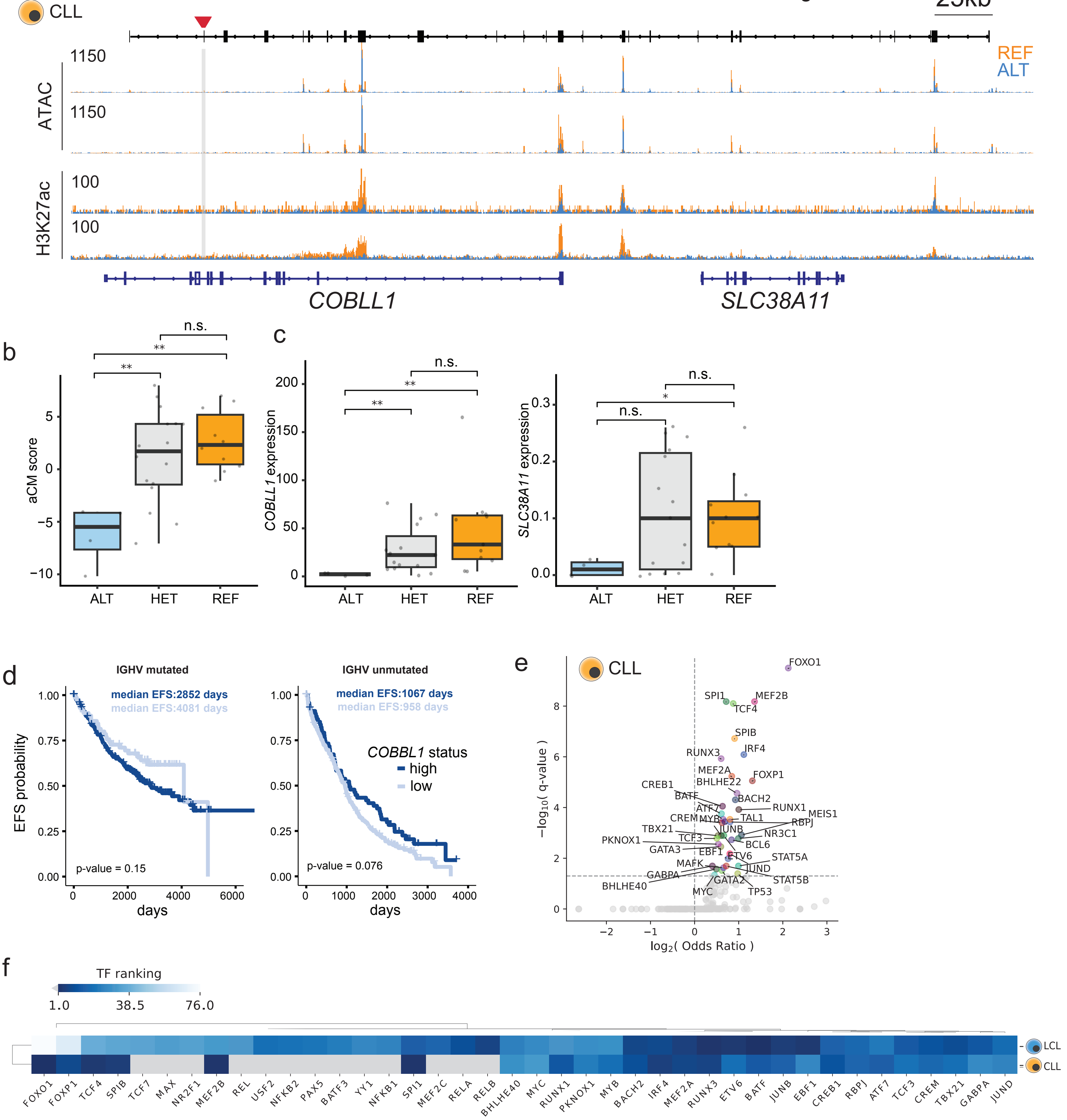
a
CLL
25kb
REF
ALT
ATAC
1150
1150
H3K27ac
100
100
COBLL1
SLC38A11
b
aCM score
n.s.
**
**
ALT
HET
REF
c
COBLL1 expression
SLC38A11 expression
n.s.
*
d
IGHV mutated
median EFS:2852 days
median EFS:4081 days
p-value = 0.15
IGHV unmutated
median EFS:1067 days
median EFS:958 days
COBLL1 status
high
low
p-value = 0.076
EFS probability
days
e
CLL
−log10(q-value)
log2(Odds Ratio)
FOXO1
SPI1
MEF2B
TCF4
SPIB
RUNX3
IRF4
MEF2A
FOXP1
BHLHE22
CREB1
BATF
BACH2
ATF7
RUNX1
MEIS1
CREM
MYB
TAL1
RBPJ
TBX21
JUNB
NR3C1
PKNOX1
TCF3
BCL6
GATA3
EBF1
ETV6
STAT5A
MAFK
JUND
GABPA
GATA2
STAT5B
BHLHE40
MYC
TP53
f
TF ranking
1.0
38.5
76.0
LCL
CLL
FOXO1
FOXP1
TCF4
SPIB
TCF7
MAX
NR2F1
MEF2B
REL
USF2
NFKB2
PAX5
BATF3
YY1
NFKB1
SPI1
MEF2C
RELA
RELB
BHLHE40
MYC
RUNX1
PKNOX1
MYB
BACH2
IRF4
MEF2A
RUNX3
ETV6
BATF
JUNB
EBF1
CREB1
RBPJ
ATF7
TCF3
CREM
TBX21
GABPA
JUND

Fig S5.2. Distinct CMs are formed in CLL and LCL. **a.** Example depicting the *COBLL1* locus which is induced in a subset of CLL patients. **b.** CM activity stratified by genotype of the highest-ranked candidate associated variant. **c.** Expression of *COBBL1* and *SLC38A11* stratified by genotype of the highest-ranked candidate-associated variant. **d**. Event-free survival of CLL patients stratified on *COBLL1* expression for both IGHV-mutated and -unmutated CLL status. P-values were obtained using a log-rank test. **e.** Log2 odds ratio versus -log10 q-value of TFBS enrichment within individual cell type when contrasting mapped CM peaks vs simulated CM peaks in CLL (yellow cell). **f.** Heatmap showing the TF ranking for those TFBSs that passed the q-value threshold (0.05) in at least one of the cell types indicated on the right side. Gray color indicates non-significant hits as defined in (**e.**). P-value indications are non-significant (ns) for p-value > 0.05, * for 0.01 < p-value ≤ 0.05, ** for 0.001 < p-value ≤ 0.01.
