# Supplementary material for: Non-coding variants impact cis-regulatory coordination in a cell type-specific manner
Source: Genome Biol. 2024 Jul 18;25:190. doi: 10.1186/s13059-024-03333-4 (PMC11256678; doi:10.1186/s13059-024-03333-4)
Supplement: Supplementary file 9 — Additional file 9. Peer review history. [file 13059_2024_3333_MOESM9_ESM.docx]

**Review history**

**First round of review**

**Reviewer 1**

Pushkarev and colleagues compared three methods to identify chromatin modules in one data set and then applied the selected method to identify chromatin modules (CM) in existing data for five blood cell types. They compared the CMs to expression of nearby genes, identified transcription factors in CM regions, and showed examples at genome-wide association study loci at which molecular QTL had previously been identified. While many of the principles observed are consistent with previous reports, the specific findings and examples are extensive and new.

This is the first systematic approach to compare the three methods on the same dataset, which includes two histone ChIP-seq marks measured in 317 lymphoblastoid cell lines, and the first to comprehensively compare CM in the 5 cell types. The 106 GWAS loci that colocalized with cmQTL, the 69 surface marker loci with CMs, and the regulatory elements detected in disease provide possible mechanisms by which the noncoding variants function.


1. How do the parameters of the three methods affect CM discovery? Were only default parameters used or was each method optimized for this data set? Do the methods differ in their dependence on sequencing depth or other aspects of data quality that may affect choice of the best method with other input data?

2. The conclusion (lines 106-109) that the number of identified modules is saturated does not seem consistent with the data presented (figure S1.1d), at least for PHM. The PHM line appears to show that larger sample sizes beyond ~300 would continue to identify additional modules, as would be expected for a method that uses genetic variants in the analysis. The conclusion should be revised.

3. Methods for mapping QTLs should be further described. What density of genetic variant data were used? What window size was used around histone marks or modules?

4. For the conclusion on lines 251-253, does the gain in detection of genes correlated with CM vs individual peaks simply depend on sequencing depth and thus power to detect correlation? Does down-sampling of CM reads to be equivalent to a single peak remove the gain?

5. The finding that many CM are not affected by the 'focal genetic variant' depends substantially on how that variant was selected. At these loci, are genetic variants present in the other CM peaks and are they correlated with the 'focal' variant? Similarly, how do the thresholds for defining a QTL influence the conclusion that [only] half of CMs are linked to a variant? Is genotype imputation comprehensive enough to identify all candidate causal variants?

6. Two examples of CLL-dependent associations are described at OSBPL5 and COBBL1. How many total disease-dependent loci were detected? Please add discussion of the relative value of this strategy to characterize GWAS loci compared to the other strategies.

7. The many examples of associations and colocalizations would be strengthened by inclusion of experimental data that validate the effects of altered modules or variants on gene expression. If experimental studies are outside the scope of this manuscript, then wording should be more cautious that assuming that association means 'impact' or that modules 'alter' expression.


Minor comments:
1. In Figure 2b, the ChIP-seq tracks for two individuals on the same line is very small and hard to see. Can the colors be more distinct? The legend should explain if the bars are stacked or overlaid, and if overlaid, which color is plotted last/on top. If space permits, separate tracks for the two individuals would be even better. Consider using more informative labels of tracks than id numbers, which are not needed for interpretation.

2. Line 363-364 says that a variant is blood-cell specific and should be reworded. The variant is present in genomes of all cell types. Is the intended wording that the cmQTL association is cell-type specific?

3. The abbreviation IGHV needs to be defined (line 440, Figure 5)

**Reviewer 2**

In the manuscript titled "Non-coding variants impact cis-regulatory coordination in a cell specific manner", Pushkarev and colleagues perform a comprehensive evaluation of a number of established methods (VCMtools, Clomics and PHM) designed for the analysis of hubs of covarying cis-regulatory elements (CREs), referred to as chromatin modules (CM). The authors apply the three approaches using a published dataset, and they conclude that Clomics stands out as the most suitable tool for CM mapping based on several metrics.

Pushkarev et al. further investigated epigenomic and genotype data from five different cell types using the Clomics-derived CMs. The authors characterised the obtained CMs and illustrated their analyses with specific examples that showcased the potential role of CMs in regulating gene expression, emphasising their putative implications in disease onset and progression.

The study has value and is well aligned with the scope of Genome Biology. The major aspects that must be addressed before the article can be considered for publication are listed below, followed by a list of minor aspects that also need to be considered.


Major points:

1. One major aspect of the paper is the claim to provide a resource and general guidelines for selection of a suitable approach for the detection of CMs. This part of the paper is currently not visible enough, and the manuscript should be revised to make it clearer how the tools need to be tuned for the data and which settings are recommended based on the author's experiences. The recommendations should focus on the quality and reproducibility of the result sets, ideally assessed by a synthetic dataset or by the subsamples, instead of runtime or usability. The authors should ideally consider adding a visual aid such as a flowchart or diagram to help readers better understand the guidelines and which parameters are advised.

2. The selection of Clomics is not comprehensible from reading about the usability and runtime of the tools and is also not evident from the metrics presented in the supplementary figures. In addition to giving the -by far- largest result set, the authors emphasise that Clomics is a good method by showing one specific locus selected based on previous experimental studies. Here, it would be useful to understand how relevant this example is to the rest of the CMs identified only by Clomics. The authors need to acknowledge the limitations of Clomics and assess the true positive and false positive rate by checking the Clomics-specific CMs more thoroughly. SFig.1H shows that Clomics has a low reproducibility between iterations, particularly in contrast to VCMtools, which needs some more interpretation. From the same point of view, it must be made clear whether the CM sets defined in Fig. 1c centre are based on results from a single call or reflect the robust CMs from all 5 iterations.

3. To accept that Clomics-specific CMs are truly more interesting than the CMs detected by all three methods requires a more detailed genome-wide summary and description of CMs detected by all methods, as well as the method-specific hits along with their associated genes. The intersection of robustly detected CMs from three tools could help to understand if Clomics-specific CMs yield a different biology than commonly detected modules. Additionally, the authors could include correlations of CM activity (especially for method-specific CMs) and gene expression. This will test if CMs specific to the Clomics method show a higher correlation with gene activity compared to the common CMs or CMs specific to the other methods.

4. The authors must also provide a general summary of the peak datasets, including quality control metrics such as the number of mapped reads and the distribution of peaks called in each cell type. This aspect is particularly important to ensure that the variations in the number of CMs detected between cell types are not due to technical differences between the datasets used. For instance, LCLs were reported to have the highest number of CMs but the results in SFig. 2.1. suggest that this observation could be a consequence of using a higher sample size. A bootstrapping approach on downsampled samples will help to assess how much of the detected CMs are due to biological differences or result from an increased sample number.

5. The expression state of a gene linked with a CM is an important aspect of establishing the CM-gene connection. The manuscript currently misses a clear explanation of how CMs are annotated with gene expression data, whether each CM is allocated to only one gene or how this information is incorporated. Further detail needs to be added to make clear how the RNA-seq was matched to the same individuals as chromatin data, and how the RNA-Seq was processed and normalised across cells and cell types.

6. In the analysis part of the paper, the writing usually follows the same principle of first introducing interesting examples known in the literature followed by showcasing them in their data. Here, the paper would clearly benefit from a more general analysis of results, with a clear focus on the CMs instead of only providing examples. Often, it remains unclear how the examples are chosen (e.g. Fig5b), or how representative they are for the dataset. The authors must provide a clear summary of how often the same biological principle is observed in the top-ranking candidates of that particular analysis (e.g. top x% from identified groups, such as CM subgroups, strongest QTLs, etc.), or genome-wide.

7. The figures are visually very appealing. However, some plots are difficult to digest and I would suggest replacing circle diagrams with bar charts and Venn diagrams with upset plots to increase the readability of the data and also to ease cross-panel comparisons of the data (e.g. Fig1c center, SFig. 3.2).

8. The authors must clarify how common anchors are defined. For example, in Fig. 2c, the bottom diagram indicates that common anchors are found in all cell types, but in SFig 2.3c diagram anchors are defined based on pairwise comparisons.

9. The manuscript also lacks an explanation of why a combination of Micro-C and Hi-C datasets is used, and exactly how these two very different data types are combined in the analysis.

10. The supplementary tables require further clarifications and comments to ensure that they can actually serve as a resource for follow-up studies. Columns like "dummy" - even though they have only "false" entries across all lines - are actually more confusing than helpful. The authors must also add the hQTL data.

11. Overall, the paper could benefit from more concise writing, cutting down on some sections. I would suggest combining the sections on cmQTLs (lines 304-330) into the chapter "CM formation appears driven by functionally distinct groups of TFs". Likewise, the chapters in which examples of the CM implications in disease are provided (lines 384-454) could be shorter and more focused on one or two important take-home messages. Figures 4 and 5 are extremely dense and could be distilled into one figure with a concise message, whilst the descriptive parts could be moved to the supplementary material.

Minor comments

12. Figure labels and legends are often not clear. For instance, labels indicate a correlation but lack information on which dimensions are being compared (e.g. Fig 3), or track indicators are fully missing (e.g. Fig 2). In aggregating plots, sample sizes/number of individuals must be indicated, and the queried cell types or background sets used to establish correlations and enrichments must always be clear.

13. Authors must add information about how is the CMs partial overlap defined. (e.g. is it sufficient to find a single nucleotide overlap in at least one peak of the CM?)

14. In Fig1a, the schematic is not very intuitive and could be improved to serve as an outline of the manuscript. Some visual guidance on why some peak combinations are detected as CMs would be very helpful.

15. Fig. 2b should be revised to make the intention clearer. It would be helpful to annotate the peak tracks so it is evident which peaks are part of the modules in each cell type and whether those peaks are part of a CM.

16. Fig 2c - the label "not reproducible" is confusing. To make it distinct from the reproducibility shown in SFig. 1.1h, I suggest using "method-specific" instead.

17. SFig. 2.2a could be added to the main figure so that it becomes clear that LCLs are used to define the anchors.

18. SFig. 2.1.b shows that Clomics has a high number of method-specific CMs. However, the difference between VCMTools and PHM appears to be cell-type dependent. The purpose of the plot should be made clear to the reader. Was it to compare different cell types or/and methods? Either way, the plots might be easier to interpret if split based on the intended message. I would advise removing the correlations from the plot since only a few data points are available and the correlation lines are certainly driven by the high counts observed in the LCLs with many samples.

19. The plots in Fig 3b/SFig3.1a-d are difficult to interpret. In addition to suffering from heavy overplotting and using too big dot sizes, the presented groups also have very different properties. Since the "closest not CM peak" is certainly always at the TSS, it should be compared to the subset of closest CM peaks. The current plot suggests that any peak is as strongly correlated as CM peaks around the TSS while more distal CM peaks cannot be evaluated as a matched control group is missing.

20. line 325 - not clear why ASB is an indicator of TF density

21. line 320 - missing reference for the statement

22. Fig. 5e - what is represented by each data point?

**Authors’ response to reviewers**

Reviewer #1:
Pushkarev and colleagues compared three methods to identify chromatin modules in one data set and then applied the selected method to identify chromatin modules (CM) in existing data for five blood cell types. They compared the CMs to expression of nearby genes, identified transcription factors in CM regions, and showed examples at genome-wide association study loci at which molecular QTL had previously been identified. While many of the principles observed are consistent with previous reports, the specific findings and examples are extensive and new.
This is the first systematic approach to compare the three methods on the same dataset, which includes two histone ChIP-seq marks measured in 317 lymphoblastoid cell lines, and the first to comprehensively compare CM in the 5 cell types. The 106 GWAS loci that colocalized with cmQTL, the 69 surface marker loci with CMs, and the regulatory elements detected in disease provide possible mechanisms by which the noncoding variants function.

1. How do the parameters of the three methods affect CM discovery? Were only default parameters used or was each method optimized for this data set? Do the methods differ in their dependence on sequencing depth or other aspects of data quality that may affect choice of the best method with other input data.

We thank the reviewer for this question. In the original manuscript, we used the default window size parameters for CM mapping with all methods. Clomics (200 peaks, corresponding to a ~1Mb window centered at the peak (Delaneau et al., Science, 2019), VCMtools (1Mb; 500 kb left and right of the peak) and PHM (1Mb; 500 kb left and right of the peak)). These window sizes are commonly used in the field for mapping local cis-regulatory interactions leveraging the observation that interactions between CREs preferentially occur within TADs (median size ~1Mb). We have now also specifically added this information to the methods section (Lines 157-160, 603-605). To identify significant peak-to-peak correlations/associations, we used the default background correlation for Clomics (value of 3). The p-value and posterior probability thresholds were selected empirically for VCMtools and PHM respectively, based on the LCL data. This resulted in the empirical p-value threshold for VCMtools (Benjamini-Hochberg adjusted p-value 0.001) and posterior probability threshold for PHM (0.8), which we used for CM mapping across all cell types.
To address the Reviewer’s question in a systematic way, we performed CM mapping for a range of window sizes and peak interaction thresholds. It should be noted that the original Clomics software did not allow adapting the window parameter explicitly, so we adapted the Clomics script accordingly. We have included the updated version of Clomics on GitHub (https://github.com/DeplanckeLab/Chromatin_modules).

Parameter searching for each CM mapping approach was performed within the following ranges for LCLs:

1. Clomics: background correlation threshold (1.5 – 4, step=0.5) and the number of peaks per window (starting at 25, and then 50 – 400 peaks).

2. VCMtools: p-value thresholds (1e-7 – 1e-2, geometric progression with the common ratio=0.1, and a max value of 0.05) and window sizes (10 kb – 1Mb).

3. PHM: posterior probability threshold (0.5 – 0.9, step=0.1, and a max value of 0.99) and window sizes (10 kb – 1Mb).

We aggregated the summary statistics for each method and each parameter in the newly added Fig S1.2-1.4. Together, we observed better robustness of Clomics with respect to the number of mapped CMs upon changes in parameter thresholds. Based on these analyses, we provide recommended parameter ranges for CM mapping for each method and added insightful Tables and Figures describing the impact of each parameter (Fig S1.3, S1.4 and Table S2).
To answer the last question of the Reviewer, we down sampled the data to 10, 20, and 25 million reads, mapped CMs with all tools and compared the summary statistics across methods and down-sampled versions (Fig S1.2). All tools showed a similar pattern with respect to the number of down-sampled reads. While 10 million reads already yielded many CMs, the number of mapped modules, as well as their similarity across different batches was the largest for 20 and 25 million reads. This indicates that sequencing 20 million reads is already sufficient for mapping of CMs, although deeper sequencing may allow the identification of additional CMs. Based on these findings, we now add the recommendation to sequence at least 20 million reads per sample, which in fact aligns with the observations and recommendations from the ENCODE consortium (Landt, Genome Research, 2012).
We have further adapted the text (lines 124-155) to make it more comprehensive and extended the section on method stability with respect to the parameters, which are now summarized in Fig S1.1, S1.2-4 and Table S2. We have also expanded the GitHub page of the project by adding tutorials on CM mapping. To further support the availability of the methods and data, we have developed an intuitive webserver (reachable at chromo.epfl.ch) that allows users to query the CMs and respective cmQTLs that were mapped in the current manuscript, or to upload their own data.

2. The conclusion (lines 106-109) that the number of identified modules is saturated does not seem consistent with the data presented (figure S1.1d), at least for PHM. The PHM line appears to show that larger sample sizes beyond ~300 would continue to identify additional modules, as would be expected for a method that uses genetic variants in the analysis. The conclusion should be revised.

We thank the Reviewer for pointing this out and revised the conclusion accordingly (Lines 110-119).

3. Methods for mapping QTLs should be further described. What density of genetic variant data were used? What window size was used around histone marks or modules?

We acknowledge that it could be helpful to further elaborate on the methods used for QTL mapping and therefore added more information to the Methods section (section Mapping of quantitative trait loci (QTLs); lines 702-707). Specifically, we added the source of the genetic variants (SNP arrays for LCLs and FIBs; whole genome sequencing for Monocytes, Neutrophils and T cells). We also comment specifically on the utilized methodology and window size. There are several established approaches in the field, which include Matrix eQTL (Shabalin, Bioinformatics, 2012) and QTLtools (Delaneau et al., Nat. Com., 2017), and we used the latter as it performed well in previous benchmarking studies (Delaneau et al., Nat. Com., 2017).

4. For the conclusion on lines 251-253, does the gain in detection of genes correlated with CM vs individual peaks simply depend on sequencing depth and thus power to detect correlation? Does down-sampling of CM reads to be equivalent to a single peak remove the gain?

To answer this question, we used CMs mapped in LCLs with the data down sampled to 10, 20 and 25 million reads. We calculated the CM activity scores (aCM scores) for CMs mapped within each group and then correlated the scores with expression of the closest genes. We consistently observed across all down-sampled datasets, that the aCM score correlates more often with gene expression than the signal in individual peaks (Fig S3.1j). This indicates that the patterns we observe are not related to sequencing depth, and do not necessarily get stronger upon increasing the sequencing depth. In addition, we investigated if the aCM-gene expression correlation depends on whether the promoter or gene body is embedded in the CM. We found that the median aCM-gene expression correlation is the strongest when the promoter of a gene is embedded in the CM, and the association gets weaker when only the gene body is part of a CM. When neither the promoter nor the gene body is part of the CM, the median correlation is low (Fig S3.1k). This further shows that the aCM allows to better associate co-variable changes in chromatin with gene expression variation than using individual peaks, especially when the gene promoter is embedded in a CM. We have added these analyses to the revised manuscript (lines 311-313).

5. The finding that many CM are not affected by the 'focal genetic variant' depends substantially on how that variant was selected. At these loci, are genetic variants present in the other CM peaks and are they correlated with the 'focal' variant?

For cmQTL mapping, the variant with the best association to the CM (lowest p-value) is selected as the cmQTL. But as the reviewer implies, there are always variants that are in perfect LD (R2=1) with the cmQTL and will thus have similar association p-values. In addition, there will be variants that are still considered in LD (R2 > 0.8) but that may have slightly lower (worse) aCM score association p-values. To assess the potential impact of these LD variants, we selected the cmQTLs that locate in a peak and assessed where the variants in LD localize depending on their R2 values (R2=0.8-0.9, R2=0.9-1, and R2=1). This clearly revealed that most of the variants locate in the same CM, and that approximately half locate in the same peak. When variants in perfect LD (R2=1) are considered, these locate on average within 500bp of the mapped cmQTL and thus mostly in the same CRE (Fig S4.1e). Together, this indicates that the effects that cmQTLs have on distal peaks are unlikely to be caused by variants that are in LD. We now discuss this on lines 327-331.
Similarly, how do the thresholds for defining a QTL influence the conclusion that [only] half of CMs are linked to a variant? Is genotype imputation comprehensive enough to identify all candidate causal variants?
The variants used for LCLs and FIBs are derived from 2.5M SNP arrays (yielding information on ~8M SNPs in total after imputation) and those for Monocytes, Neutrophils and T cells from whole genome sequencing data (yielding information on ~7M SNPs). As the typical genome varies at ~4-5M sites compared to the reference genome (1000G paper, Nature, 2015), the SNP numbers we used should cover the majority of these sites. We now discuss this in the methods on lines 702-707. With respect to how many CMs are linked to a variant, we used a q-value cut-off at 0.05 to correct for multiple testing. It is conceivable that the cmQTLs detected with less stringent q-value cut-offs would become significant when the sample size is increased. We thus quantified the effect of different q-value thresholds (0.01 – 0.5) on the number of CMs with a variant and the distance of variants to their respective CMs. This revealed that while relaxing the q-value cut-off allows attributing a variant to all CMs for most cell types (Fig S4.1g), the distance between the focal variant and the CM increases substantially after q-value thresholds of 0.10-0.15 (Fig S4.1h). These associations may thus be less relevant (or at least not occurring in cis). We have now added a description of this to the legend of Fig S4.1.

6. Two examples of CLL-dependent associations are described at OSBPL5 and COBBL1. How many total disease-dependent loci were detected? Please add discussion of the relative value of this strategy to characterize GWAS loci compared to the other strategies.

We have extended our initial analyses with the quantification of the total number of loci detected in the context of CLL (n=16, now included in Fig S5a). We now also discuss this in the manuscript accordingly, please refer to lines 473-475.

7. The many examples of associations and colocalizations would be strengthened by inclusion of experimental data that validate the effects of altered modules or variants on gene expression. If experimental studies are outside the scope of this manuscript, then wording should be more cautious that assuming that association means 'impact' or that modules 'alter' expression.

While we have previously experimentally validated the involvement of a variant in CM activity modulation (Llimos et al., Nature Comm., 2022), we agree with the Reviewer that using the word ‘impact’ and related terms could be too strong for unvalidated loci, and we have adapted the text accordingly. We have in particular changed strong verbs like impact to ‘associated with’. See. for example, lines 366, 367, 388, 413, 421, 427, 449 and 478.

Minor comments:
1. In Figure 2b, the ChIP-seq tracks for two individuals on the same line is very small and hard to see. Can the colors be more distinct? The legend should explain if the bars are stacked or overlaid, and if overlaid, which color is plotted last/on top. If space permits, separate tracks for the two individuals would be even better. Consider using more informative labels of tracks than id numbers, which are not needed for interpretation.

We have adjusted the colors and the tracks to improve the interpretation. We have now retained a smaller simpler version of the panel as Figure 2b, and moved the entire panel with improved annotation and increased in size to Fig S2.2a.

2. Line 363-364 says that a variant is blood-cell specific and should be reworded. The variant is present in genomes of all cell types. Is the intended wording that the cmQTL association is cell-type specific?

Indeed, the cmQTL association is not strictly speaking blood-specific as the associations in other tissues are significant as well. We have adapted the text accordingly by stating that the variant has the strongest association with gene expression in blood cells (lines 415-416).

3. The abbreviation IGHV needs to be defined (line 440, Figure 5)

We have now defined IGHV in the text, line 484.

Reviewer #2:
In the manuscript titled "Non-coding variants impact cis-regulatory coordination in a cell specific manner", Pushkarev and colleagues perform a comprehensive evaluation of a number of established methods (VCMtools, Clomics and PHM) designed for the analysis of hubs of covarying cis-regulatory elements (CREs), referred to as chromatin modules (CM). The authors apply the three approaches using a published dataset, and they conclude that Clomics stands out as the most suitable tool for CM mapping based on several metrics.

Pushkarev et al. further investigated epigenomic and genotype data from five different cell types using the Clomics-derived CMs. The authors characterised the obtained CMs and illustrated their analyses with specific examples that showcased the potential role of CMs in regulating gene expression, emphasising their putative implications in disease onset and progression.
The study has value and is well aligned with the scope of Genome Biology. The major aspects that must be addressed before the article can be considered for publication are listed below, followed by a list of minor aspects that also need to be considered.

Major points:
1. One major aspect of the paper is the claim to provide a resource and general guidelines for selection of a suitable approach for the detection of CMs. This part of the paper is currently not visible enough, and the manuscript should be revised to make it clearer how the tools need to be tuned for the data and which settings are recommended based on the author's experiences. The recommendations should focus on the quality and reproducibility of the result sets, ideally assessed by a synthetic dataset or by the subsamples, instead of runtime or usability. The authors should ideally consider adding a visual aid such as a flowchart or diagram to help readers better understand the guidelines and which parameters are advised.
We thank the Reviewer for this suggestion, and it aligns with some of the points raised by Reviewer 1. To provide insights into the parameters for mapping of CMs, we have now explored a range of window sizes and peak association thresholds, which we consider the critical parameters as they control the false discovery rate, CM size and location in functionally relevant genomic regions. It should be noted that the original Clomics software did not allow adapting the window parameter explicitly, so we adapted the Clomics script accordingly. We have included the updated version of Clomics on GitHub (https://github.com/DeplanckeLab/Chromatin_modules).

Parameter searching for each CM mapping approach was performed within the following ranges for LCLs:

1. Clomics: background correlation threshold (1.5 – 4, step=0.5) and the number of peaks per window (starting at 25, and then 50 – 400 peaks).

2. VCMtools: p-value thresholds (1e-7 – 1e-2, geometric progression with the common ratio=0.1; max value of 0.05) and window sizes (10 kb – 1Mb).

3. PHM: posterior probability threshold (0.5 – 0.9, step=0.1, and a max value of 0.99) and window sizes (10 kb – 1Mb).

We aggregated the summary statistics for each method and each parameter (Figure S1.2-1.4). Together, we observed better robustness of Clomics with respect to the number of mapped CMs upon changes in parameter thresholds. Based on these analyses, we provide recommended parameter ranges for CM mapping for each method and added insightful Tables and Figures describing the impact of each parameter (Fig S1.3, S1.4 and Table S2).
In addition, we aimed to provide guidelines on the required sequencing depth to map CMs, as this is the costly aspect of epigenome profiling and, with the current sequencing regimes, an important limiting factor. To this end, we down-sampled the data to 10, 20, and 25 million reads, mapped CMs with all tools and compared the summary statistics across methods and down-sampled versions (Fig S1.2). All tools showed a similar pattern with respect to the number of down-sampled reads. While 10 million reads already yielded many CMs, the number of mapped modules, as well as their similarity across different batches was the largest for 20 and 25 million reads. This indicates that sequencing 20 million reads is already sufficient for mapping of CMs, although deeper sequencing may allow the identification of additional CMs. Based on this, we now add the recommendation to sequence at least 20 million reads per sample, which in fact aligns with the observations and recommendations from the ENCODE consortium (Landt, Genome Research, 2012).
We have further adapted the text (Lines 124-155) to make it more comprehensive and extended the section on method stability with respect to the parameters, which are now summarized in Figures S1.1, S1.2-1.4 and Table S2. We have also expanded the GitHub page of the project by adding tutorials on CM mapping. To further support the availability of the methods and data, we have developed an intuitive webserver (reachable at chromo.epfl.ch) that allows users to query the CMs and respective cmQTLs that were mapped in the current manuscript, or to upload their own data.
2.1. The selection of Clomics is not comprehensible from reading about the usability and runtime of the tools and is also not evident from the metrics presented in the supplementary figures. In addition to giving the -by far- largest result set, the authors emphasise that Clomics is a good method by showing one specific locus selected based on previous experimental studies. Here, it would be useful to understand how relevant this example is to the rest of the CMs identified only by Clomics. The authors need to acknowledge the limitations of Clomics and assess the true positive and false positive rate by checking the Clomics-specific CMs more thoroughly. SFig.1H shows that Clomics has a low reproducibility between iterations, particularly in contrast to VCMtools, which needs some more interpretation. From the same point of view, it must be made clear whether the CM sets defined in Fig. 1c centre are based on results from a single call or reflect the robust CMs from all 5 iterations.
We agree that the manuscript would benefit from better explaining the rationale underlying our tool prioritization. To better understand the stability of the methods and their performance with respect to the quantitative outputs, we expanded the method comparison section with the following analyses: 1. Evaluation of the effect of varying the total number of reads on CM mapping, and 2. Evaluation of the method parameter effects on CM mapping, lines 110-119, 124-155. One of the critical assumptions behind the window size of 1Mb is that cis-regulatory interactions tend to be restricted by TAD boundaries. Therefore, we included the chromosome conformation data on A/B compartments and TADs into the step of parameter selection under the assumption that CMs should be mostly located in active A compartments, and fall into or overlap with TADs as observed previously (Waszak et al., Cell, 2015; Delaneau et al., Science, 2019). Together, the metrics that we used to assess method performance were:
1. the Number of mapped CMs, 2. the Median CM length, 3. the Coefficient of Variation (CV) of CM length, 4. Average CM reproducibility scores across the set of parameters, 5. the Fraction of CMs overlapping A/B compartments or TADs, 6. the Fraction of CMs overlapping genes and the Median association strength of the aCM score with embedded gene expression.

Number and size of CMs:
The methods differed by their sensitivity to the selected thresholds. For all methods, an increase in the stringency of the significance cut-off led to a lower number of CMs mapped with a shorter length, which is intrinsic to how the methods identify CMs and leads to the retention of only those CMs with the strongest peak-to-peak associations. In terms of window size, increasing it leads to more and increasingly longer CMs in VCMtools and PHM, while for Clomics, the number and length of the CMs remain relatively constant. This consistency of Clomics under parameter change suggests that CMs mapped with this approach are thus more likely to capture biological signal (Figure S1.3a-c).

Average CM reproducibility scores across the set of parameters:
We quantified the reproducibility of the mapped CMs within each method across the range of parameters. This indicated the robustness of Clomics outputs (i.e., an F1 score of overall average reproducibility = 0.545), as compared to VCMtools (F1 score = 0.208) and PHM (F1 score = 0.365). PHM showed the highest reproducibility scores for the posterior probability thresholds > 0.7 across all window sizes, indicating that the approach is robust under a stringent cut-off, whereas VCMtools requires more careful selection of both window sizes and p-value cut-offs to allow for consistent detection of CMs (Fig S1.4).

Fraction of CMs overlapping A/B compartments or TADs:
An informative quality indicator for CMs mapped using a given set of parameters is to what extent these CMs partially/fully overlap TADs and A/B compartments, as such overlap is expected and was also observed with the parameters that we used in the original manuscript. When we varied the parameters, we observed that in particular VCMtools showed variability in the percentage of CMs overlapping different categories with low overlap when using smaller window sizes (<0.25Mb) and more stringent p-value thresholds. PHM showed consistent proportions of overlap for window sizes less than 0.25Mb and more variability across posterior probability thresholds with increasing window size, whereas Clomics was relatively consistent across windows sizes and p-value cut-offs > 2 (Fig S1.3d-f).

Altogether, these analyses indicate that Clomics is the most consistent tool for CM mapping as it is least sensitive to variation of the p-value threshold and window size parameters. Our findings also provide support for the parameters that we used in the original manuscript. This is because they yield a good balance between the number of detected CMs and overlap with genomic features such as TADs and A/B compartments, while retaining stringent significance cut-offs. Based on the metrics presented here, we defined ranges of parameters as well as suggested values for each method as a guideline for CM mapping (Table S2).

2.2. SFig.1H shows that Clomics has a low reproducibility between iterations, particularly in contrast to VCMtools, which needs some more interpretation.

Indeed, CMs mapped with VCMtools showed higher average reproducibility across pairwise comparisons of CMs for different sample sizes. To investigate this further, we explored how well the methods preserve CM peak composition across randomized sample size groups with respect to the number of peaks in CMs. Specifically, in the randomized setting for chr22, per sample size group, for each CM mapped with the full set of samples, we calculated the average of CM peak occurrences in 5 randomized calls as the number of times the exact CM peak appeared in randomized calls to the total number of calls. This analysis showed that VCMtools preserves the CM peak composition also for smaller CM sizes (2-3 peaks), whereas Clomics trends toward higher average scores for CMs with a larger number of peaks and larger sample sizes (now included in Fig S1.1i). This can be explained by the fact that Clomics calculates the background correlation threshold on a per chromosome basis by considering all pairwise peak correlations and dynamically defines the cutting node of the tree where overall peak correlations are X times (default = 3) higher than the background. The background threshold may vary depending on the sample composition and small changes in peak correlations may affect the definition of a “cutting” node, and therefore CM peak composition. This may lead to splitting of the “supposed to be” CMs into smaller modules, and consequently, to CMs with lower, as compared to VCMtools, average peak correlations (Fig S1.2b). VCMtools on the other hand ignores the full chromosomal context and computes correlations only within sliding windows of XMb (default=1Mb), which, together with a stringent p-value threshold, ensures the detection of strong associations. Altogether, this rationalizes the lower reproducibility scores across randomized sample batches within Clomics, as compared to VCMtools. We would also like to note that the reproducibility score that we define as the F1 score on the Jaccard indices for CM pair peak overlaps greatly penalizes partially overlapping peaks, which pushes the final score farther away from 1 to 0. For the observations related to amongst others the parameter testing and relation of aCM to gene expression, we consider Clomics as the tool to recommend. However, we do acknowledge that the lower reproducibility upon subsampling may be a limitation of Clomics compared to VCMtools and comment on this in the discussion section of the manuscript (lines 501-509).

To address the concerns of the Reviewer regarding the relevance of the example shown in Figure 1c to the rest of the CMs identified only by Clomics, we would like to combine the answers to this point with the answer to the questions raised in pt.”3: To accept that Clomics-specific CMs are truly more interesting than the CMs detected by all three methods requires a more detailed genome-wide summary and description of CMs detected by all methods, as well as the method-specific hits along with their associated genes. The intersection of robustly detected CMs from three tools could help to understand if Clomics-specific CMs yield a different biology than commonly detected modules. Additionally, the authors could include correlations of CM activity (especially for method-specific CMs) and gene expression. This will test if CMs specific to the Clomics method show a higher correlation with gene activity compared to the common CMs or CMs specific to the other methods.”

To define whether Clomics-specific CMs can aid in advancing our understanding of gene regulation, we compared how well CM activity (defined by the respective aCM score) correlates with gene expression for the CMs mapped using each method. We observed that Clomics in general captures a higher percentage of significant aCM-gene expression correlations compared to VCMtools and PHM (Fig S1.5j), which also extends to the Clomics-specific CMs (Fig S1.5k) and is independent of the sequencing depth (Fig S3.1j). This could in part relate to the fact that Clomics also captures a higher percentage of genes, including gene promoters embedded into CM CREs (Fig S1.2h-i), which in turn renders Clomics CMs, both the shared and specific modules, capture more CREs relevant to regulating gene expression compared to VCMtools and PHM. Intersection of the gene categories embedded in CMs (shared or specific to a particular method) revealed that cell type-specific genes (which we postulate to be enriched in CMs in general) are mainly captured in the ‘shared between methods’ groups and that only Clomics-specific CMs are associated with additional GO terms, which may also be partly related to B cell biology such as “Chronic Myeloid Leukemia” (Fig S1.7).
Together, these analyses show that mapping CMs with Clomics allows increasing the set of genes for which gene regulatory information can be inferred, as Clomics capture more CMs for which a higher proportion shows a significant correlation between the aCM score and respective gene expression, lines 176-191, 199-201, 203-206.

“… it must be made clear whether the CM sets defined in Fig. 1c centre are based on results from a single call or reflect the robust CMs from all 5 iterations.
This overlap is based on CMs mapped by a single call using all data for each method. We adapted this in the text accordingly (lines 157-160).

4. The authors must also provide a general summary of the peak datasets, including quality control metrics such as the number of mapped reads and the distribution of peaks called in each cell type. This aspect is particularly important to ensure that the variations in the number of CMs detected between cell types are not due to technical differences between the datasets used. For instance, LCLs were reported to have the highest number of CMs but the results in SFig. 2.1. suggest that this observation could be a consequence of using a higher sample size. A bootstrapping approach on downsampled samples will help to assess how much of the detected CMs are due to biological differences or result from an increased sample number.

To generate a peak set to compare samples throughout the manuscript, we had performed the following in the original manuscript: For each cell type, we used 10 individuals with 15 million reads to create a merged bam files (one for each mark, 150M reads in total). We then merged these merged bam files into a ‘meta bam file’ (750M reads in total), which thus contains 50 individuals in total for 5 different cell types. We called peaks on these meta bam files, enabling the generation of a meta peak set that should not be impacted by differences in quality or read counts. All downstream analyses were based on this merged peak set. We now discuss this in more detail in the Methods section (Lines 582-591) and have added a table that shows the total reads per sample and the number of reads falling into peaks (Table S3).

To assess if read counts were responsible for the difference in total mapped CMs among the distinct cell types, we used the down-sampled data of 10, 20 and 25 million reads for each cell type, and used this as input for CM mapping. We observed that more CMs were mapped in LCLs and FIB than in other cell types, even when we used down sampled bam files with 10 million reads per sample (Fig S.1.2a, S.2.1d). We hypothesize that one underlying reason could be that LCLs are culture-adapted and more homogeneous, while, for example, the primary blood cells (Monocytes, Neutrophils and T cells) were taken directly from human donors and are thus subject to more variability in general. We now discuss these findings on line 221-227. In addition, these analytical strategies supported our original conclusions on the minimum required number of samples for 20 and 25 million reads, and with 10 million reads the requirement changes to a minimum of ~200 samples for Clomics and VCMtools, and at least 300 for PHM (Fig S1.2b-e).

5. The expression state of a gene linked with a CM is an important aspect of establishing the CM-gene connection. The manuscript currently misses a clear explanation of how CMs are annotated with gene expression data, whether each CM is allocated to only one gene or how this information is incorporated. Further detail needs to be added to make clear how the RNA-seq was matched to the same individuals as chromatin data, and how the RNA-Seq was processed and normalised across cells and cell types.

We thank the reviewer for this suggestion. We have now improved the description part of the CM annotation with respect to expression data on lines 97, 211-212 and extended the Methods section with additional details regarding the RNA-seq data processing (lines 578, 580-581).

6. In the analysis part of the paper, the writing usually follows the same principle of first introducing interesting examples known in the literature followed by showcasing them in their data. Here, the paper would clearly benefit from a more general analysis of results, with a clear focus on the CMs instead of only providing examples. Often, it remains unclear how the examples are chosen (e.g. Fig5b), or how representative they are for the dataset. The authors must provide a clear summary of how often the same biological principle is observed in the top-ranking candidates of that particular analysis (e.g. top x% from identified groups, such as CM subgroups, strongest QTLs, etc.), or genome-wide.

When preparing the figures of the manuscript, we felt it was important to communicate to the readers not only how CMs were mapped, but also how CMs can be observed in a locus, which types of peaks and genes are embedded and where the QTL is located. Some of the examples we used in the manuscript were chosen based on manual curation to provide representative CM cases. Where possible, we have now included the reasoning as to how specific CM examples were chosen. For CLL specifically, we have now included the analyses that allowed us to shortlist the examples presented in Figure 5 and Figure S5.1-5.2 (now included in Fig S5.1a).

7. The figures are visually very appealing. However, some plots are difficult to digest and I would suggest replacing circle diagrams with bar charts and Venn diagrams with upset plots to increase the readability of the data and also to ease cross-panel comparisons of the data (e.g. Fig1c center, SFig. 3.2).

We have adapted the figures according to the Reviewer’s suggestions. Please find the updated panels in the revised manuscript (changes in the main involve Fig 1a,c; Fig 2a,b; Fig 3b). We have also extended the figure legends to improve the interpretation of the figure panels.

8. The authors must clarify how common anchors are defined. For example, in Fig. 2c, the bottom diagram indicates that common anchors are found in all cell types, but in SFig 2.3c diagram anchors are defined based on pairwise comparisons.

The common anchors are defined based on the intersection of all pairwise combinations across cell types of the differential TFBS enrichment analysis for common CM peaks vs the union of cell type-specific CM peaks. We have adapted the description in the main text of the manuscript (Line 267).

9. The manuscript also lacks an explanation of why a combination of Micro-C and Hi-C datasets is used, and exactly how these two very different data types are combined in the analysis.

We used Micro-C (500 bp resolution) and ultra-deep Hi-C (also at 500bp resolution) as complimentary data sets. This is because Micro-C relies on a somewhat different sample preparation strategy compared to Hi-C, allowing us to assess if the observations are related to or affected by the type of utilized chromosome conformation-capturing methodology. The agreement between the Micro-C and Hi-C-derived results can now be found in Fig S1.5d.

10. The supplementary tables require further clarifications and comments to ensure that they can actually serve as a resource for follow-up studies. Columns like “dummy” – even though they have only “false” entries across all lines – are actually more confusing than helpful. The authors must also add the hQTL data.

We have adapted the supplementary tables (Tables S1-S7) to make them more comprehensible and added a new table with the hQTL data (Table S6).

11. Overall, the paper could benefit from more concise writing, cutting down on some sections. I would suggest combining the sections on cmQTLs (lines 304-330) into the chapter "CM formation appears driven by functionally distinct groups of TFs". Likewise, the chapters in which examples of the CM implications in disease are provided (lines 384-454) could be shorter and more focused on one or two important take-home messages. Figures 4 and 5 are extremely dense and could be distilled into one figure with a concise message, whilst the descriptive parts could be moved to the supplementary material.

We have adapted the text to make it more accessible and precise, resulting in total in a ~20% shorter Results section. We decided to keep Figures 4 and 5 separate as they are meant to convey different messages but we have moved some panels to the supplementary figures to increase their readability and interpretation (for example we moved two panels from main Figure 5 to Fig S5.1-5.2).

Minor comments:
12. Figure labels and legends are often not clear. For instance, labels indicate a correlation but lack information on which dimensions are being compared (e.g. Fig 3), or track indicators are fully missing (e.g. Fig 2). In aggregating plots, sample sizes/number of individuals must be indicated, and the queried cell types or background sets used to establish correlations and enrichments must always be clear.

We have adapted the labels and legends of the Figures to make them clearer and more interpretable.

13. Authors must add information about how is the CMs partial overlap defined. (e.g. is it sufficient to find a single nucleotide overlap in at least one peak of the CM?)

Indeed, the partially overlapping CMs are identified based on at least a single nucleotide overlap. We have adapted the Methods section, lines 643-645, as well as the legend of Figure 1 to mention this explicitly.

14. In Fig1a, the schematic is not very intuitive and could be improved to serve as an outline of the manuscript. Some visual guidance on why some peak combinations are detected as CMs would be very helpful.

We have adapted panel “a” of Figure 1 to make it more self-explanatory.

15. Fig. 2b should be revised to make the intention clearer. It would be helpful to annotate the peak tracks so it is evident which peaks are part of the modules in each cell type and whether those peaks are part of a CM.

We have adapted and moved the original panel 2b to the supplement (now Fig S2.2a) and kept only the CMs mapped in the different cell types (new Figure 2b) which makes it more focused on the key message and thus hopefully clearer to the reader.

16. Fig 2c – the label “not reproducible” is confusing. To make it distinct from the reproducibility shown in Sfig. 1.1h, I suggest using “method-specific” instead.

We have adapted the labels according to the Reviewer’s suggestion.

17. Sfig. 2.2a could be added to the main figure so that it becomes clear that LCLs are used to define the anchors.

We would like to emphasize the difference between the analyses explained in lines 250-265 and lines 267-281. To assess the episomal regulatory activity of the CREs, we used available STARR-seq data from the GM12878 LCL cell line. We performed pairwise comparisons between LCLs and every other cell type to stratify the CM-embedded CREs into those shared between the compared pair of cell types (i.e. CREs that we will refer to as ‘anchors’) and those specific to one of the cell types (i.e. CREs that we will refer to as “non-anchors”) (Fig S2.4a,c). The following analysis (lines 267-281) of TFBS enrichment focuses all possible pairwise comparisons across cell types to define “anchors”, which we also illustrate in Fig S2.4c. Together, we would like to retain the Fig S2.2a in its original position (now present in S2.4a) as we believe it enhances the clarity and differentiation between the two types of analyses.

18. SFig. 2.1.b shows that Clomics has a high number of method-specific CMs. However, the difference between VCMTools and PHM appears to be cell-type dependent. The purpose of the plot should be made clear to the reader. Was it to compare different cell types or/and methods? Either way, the plots might be easier to interpret if split based on the intended message. I would advise removing the correlations from the plot since only a few data points are available and the correlation lines are certainly driven by the high counts observed in the LCLs with many samples.

We have adapted panel S2.1b by splitting the plot based on the method and removing the linear fit. The plot now shows the differences between the cell types in terms of number of CMs mapped with each method.

19. The plots in Fig 3b/SFig3.1a-d are difficult to interpret. In addition to suffering from heavy overplotting and using too big dot sizes, the presented groups also have very different properties. Since the "closest not CM peak" is certainly always at the TSS, it should be compared to the subset of closest CM peaks. The current plot suggests that any peak is as strongly correlated as CM peaks around the TSS while more distal CM peaks cannot be evaluated as a matched control group is missing.

We have adapted the panels by adding violin plots showing the total difference between the groups and replaced the scatter plots with line plots to prevent overplotting. In addition, we now show the comparison between the closest CM peak and closest non-CM peak, as well as the closest peak vs the ‘strongest’ CM peak (Fig 3b, Fig S3.1a-d).

20. line 325 - not clear why ASB is an indicator of TF density

The argument was that if a QTL would be associated with more ASB, that this would imply greater TF binding enrichment. However, we understand that this statement can be confusing and have therefore removed the sentence.

21. line 320 - missing reference for the statement

We have added the reference to the statement (Line 376).

22. Fig. 5e - what is represented by each data point?
Each data point represents an individual. We have adapted the legend of Figure 5e (now S5.1c) to make this clear.

**Second round of review**

**Reviewer 1**

The authors have addressed my comments and extended the value of the manuscript

**Reviewer 2**

We would like to thank the authors for their efforts to answer our concerns and questions. The current version of the manuscript shows considerable improvements. Although most of our major points have been addressed in the new version of the manuscript, some aspects still require further clarification before publishing.

1. One of the earlier major concerns was the insufficient information regarding the parameters used for the three methods tested, making it difficult to assess whether Clomics is the best choice to call CMs. We acknowledge the extensive efforts put into testing and, in the case of Clomics, implementing different parameters. However, it is still not clear how the range of the parameter search was picked for each method, to be convinced that the choice of paramenters was fair. For instance, does the same search space apply to other cell types?

2. Another challenging aspect of the current manuscript is how to interpret the results when the parameters used in the methods were different. For instance, VCMtools and PHM use windows in Mb, whilst Clomics uses peaks within windows. First, why were different window sizes tested between VCMtools and PHM? Is the number of peaks in Clomics equivalent to a certain window size in VCMtools/PHM? How were the parameters picked for each method (e.g. the step size)? Could the authors elaborate on the choices made to more convincingly reassure that the comparisons across methods is fair with the chosen parameters?

3. What do red boxes represent in Figures S1.3 to S1.4? Are the red boxes highlighting the optimal conditions for the three methods compared? How were these chosen?

4. The integration of CM modules with 3D chromatin organization is very descriptive (starting on line 157), and could be strengthened by providing the enrichment over the expected values, to improve the quality of the analysis and help showcase that CMs are enriched in particular features of chromatin organization. For example, CMs overlap 25.7-31.9% with the B compartment and 59.4-63.9% with the A compartment. Is this overlap significantly different than expected, given the typical proportion of A and B compartments in the genome?

5. According to the text, CMs found within TAD boundaries constitute 68.1-73.2%. Are these CMs confined within TAD boundaries or just overlapping by one single base? Statements like this one require more careful clarification.

6. In lines 158-160, why is 'n' so different between methods? Why is 'n' so high in Clomics compared to the other methods? How were the other final parameters determined?

7. In Figure 1c, the CMs are compared across the three methods, showing that Clomics identified around 30% more CMs. Is the ChIP-Seq signal distribution across high and low peak activity in the Clomics-only CMs similar to those CMs identified by all methods? In other words, is Clomics more sensitive at detecting CMs with certain types/quality of peaks? Likewise, the interactions in the SPIC examples seem to be much weaker and considerably less compared to interactions detected in the MB21D2 CM.

8. In Figure S1.6a, it is not clear what is plotted as ‘gene expression’? Are these values TPMs? FPKMs? Given the difference in the number of genes that overlap CMs vs those that do not, it would be a better metric to plot the median of expression.

9. Is there a reason for using a ‘meta BAM’ file (lines 561-563) instead of taking peaks or regions that are common across different individual samples? Are the samples that were merged produced using the same technology?

10. In the section “Cell type specificity of interindividual variation” (starting on line 207), the authors have enhanced the details of gene expression data processing. However, the manuscript still lacks an explanation of how CMs are associated with genes. For instance, is each CM assigned to a single gene or multiple genes? Are they associated based on distance? Are any thresholds applied? It is essential to elaborate further and fully to enable reproducibility.

11. In lines 217-219, the authors enumerate the cells they focus on but omit LCLs. Is it a typo or is there a more specific reason to exclude LCLs? It is important to explain in the manuscript.

12. It is very interesting to see that the number of reads does not influence the rank of the cells based on the number of CMs detected (Figure 2.1d) and that this could be due to the heterogeneity of the cells. The plot is however confusing, did all cell types have the same number of ‘all reads’? Important to clarify and improve the manuscript itself on this point.

13. Figure S2.1f-j and lines 229-233: the discrepancy between the figure labelling and the text can be confusing. The figure depicts the overlapping score whilst the text mentions the similarity score. Are these two terms equivalent? Sticking with one definition option would ease the understanding of the text. It is important to fix this problem.

14. The examples shown in Figures 2b and S2.3a-c are very interesting. However, to support the more generalised claim made in lines 235-236, these correlations should be done genome-wide. Alternatively, the statement should be more cautiously phrased to acknowledge that the claim may apply only to specific examples.

15. Details on how CREs are defined are missing (CM formation appears to be driven by functionally distinct groups of TFs’ starting from line 238). The authors only stated that the CRE annotations for LCLs were obtained from ChromHMM and SCREEN databases. How were CRE annotations from other cell types obtained? Did the CREs overlap gene promoters, exons, and introns or were exclusively extragenic? This could be important because, for instance, when comparing CMs in LCL with CMs in other cell types, the CREs identified as common anchors and that showed the highest activity, might represent gene promoters which are active in respective cell types. In contrast, CREs identified as non-anchors could represent potential enhancer regions, which in this case would explain their reduced level of activity compared to anchor CREs and their higher degree of cell-type specificity.

16. In Figure S2.4 b legend, information on the statistical test applied is missing. Please add.

17. In Figure 2c, does “CT-restricted” stand for “cell-type-restricted”? Please add to the legend and text.

18. The manuscript still lacks a clear explanation of how anchors and non-anchors are defined (line 267). Are anchors common CREs in at least one pairwise comparison, or do they need to be common to all cell types? This explanation should be clearly stated in the Methods section. A step-by-step schematic would aid in understanding the analysis and replicating it.

19. What is the expression status of the transcription factors identified in Figures 2 and S2.4? Are all these transcription factors expressed in the cell lines compared?

20. Figure S2.5a-d panels are not mentioned in the text and can therefore be removed.

21. In Figure S2.5e, what does “p” in “pTPM” stand for?

22. In Figures 3b and S3.1a-d, the plots in the left panels display the distribution of absolute values of the correlations between peak height and gene expression for peaks in different states. While the differences between the peaks in different states are significant for most of the analysis, the median values indicate a weak correlation between peak heights and expression. We believe that these results should be interpreted accordingly, also in the text.

23. Missing reference to results section in lines 305-306.

24. In lines 305-306, the authors mention that "in all conditions, the CREs closest to TSS have the highest correlation to gene expression". This statement is partially incorrect because, according to Figures 3b and S3.1a-d, the differences in correlations between the closest CM peak and the closest non-CM peak are significant only for LCLs and Monocytes, and not for the other cell types.

25. In lines 311-313, it should be "significantly higher correlation" instead of "significantly stronger". As mentioned before, the median correlation values point to a weak correlation between CMs and gene expression. Are the CMs relevant for the study of gene expression?


Minor points:
26. Check figure legends and text for consistency in explaining abbreviations; in some cases, abbreviations are only explained after their first occurrence.

27. Double-check for typos and inconsistencies in abbreviations. For example, sometimes figures are referred to as "Figure X," while other times they are referred to as "Fig. X." Additionally, supplemental labelling is missing (i.e., S in Fig. S2.4a in line 255).

**Authors’ response to reviewers**

Reviewer #2: We would like to thank the authors for their efforts to answer our concerns and questions. The current version of the manuscript shows considerable improvements. Although most of our major points have been addressed in the new version of the manuscript, some aspects still require further clarification before publishing.


1. One of the earlier major concerns was the insufficient information regarding the parameters used for the three methods tested, making it difficult to assess whether Clomics is the best choice to call CMs. We acknowledge the extensive efforts put into testing and, in the case of Clomics, implementing different parameters. However, it is still not clear how the range of the parameter search was picked for each method, to be convinced that the choice of parameters was fair. For instance, does the same search space apply to other cell types?

We thank the reviewer for acknowledging the extensive parameter testing efforts. For each method, there were two critical parameters to test: the maximum distance between a peak pair (CREs) that is considered for association testing and the strength of such association.

To select a range of windows, we used the commonly used window size of 0.5Mb as the reference point. We would like to note here that the window size of 0.5Mb, in the parameter selection part, refers to the distance from a focal peak to any another peak to its left or right, which is equivalent to using a 1Mb window (e.g. in the PHM definition) centered at the peak. Therefore, the tested window sizes from 0.01Mb to 1Mb to the left/right of the peak correspond to respectively 0.02Mb - 2Mb windows centered at peaks. We selected the 0.25Mb step to ensure a diverse range of window sizes while avoiding extensive search space due to its computational burden in the case of multiple parameter combinations. Clomics is somewhat different in concept in terms of how it is designed and it uses a defined number of peaks around a focal peak rather than a specific genomic window. To obtain a similar window search space to VCMtools and PHM, we picked the range from 25 to 400 peaks (with the default of 200 peaks approximating a 1Mb window; see also Delaneau et al., Science, 2019).

To choose the peak association threshold per method, we also aimed to test a range of options from the least to the more stringent ones. In case of VCMtools, we started with the 0.05 p-value threshold and chose a few more stringent options up to 1e-6. One could possibly test intermediate thresholds, such as 0.005, 0.0005, etc., yet we decided to exclude those from consideration to speed up computations for all pairwise combinations of tested parameters. Clomics background correlation thresholds, as well as posterior probabilities of peak interaction under the causality hypothesis for PHM, were also selected in such a way to ensure a broad range of tested values and respective outcomes. It is thereby important to note that in the original PHM study (Kumasaka et al., Nature Genetics 2019), the authors used a 0.5 posterior probability threshold to map causal peak interactions in ATAC-seq data for 100 LCL samples. To map such causal peak interactions in ChIP-seq data, however, we observed that such threshold is quite lenient, which motivated us to implement more stringent values and to select 0.8 as our “default” option.

While the step sizes for window and peak association strength thresholds may be different between the methods and, while there still be a certain, difficult-to-avoid level of subjectivity in their selection, it is evident that, based on Figures S1.3 and S1.4, the current parameter ranges allow us to reach the low and high extremes of the measured statistics (for the distribution of sizes and number of mapped CMs). Moreover, we define reasonable parameter combinations per method with respect to the statistics of interest, which are also described in Table S2. We thereby hope that our efforts of reducing the parameter search space will be useful to prospective users when looking for their own parameter combination for a method of interest.

We have now added additional details in the legend of Fig S1.3 stating: “Across all panels, red rectangles indicate the optimal parameter combination that represents a compromise between the number of CMs versus the distribution, size and overlap with TADs and A/B compartments of these CMs”. We also added a more extensive description to the methods (lines 689-707). Note though that we have not extensively tested the full range of parameter combinations for cell types other than LCLs.


2. Another challenging aspect of the current manuscript is how to interpret the results when the parameters used in the methods were different. For instance, VCMtools and PHM use windows in Mb, whilst Clomics uses peaks within windows. First, why were different window sizes tested between VCMtools and PHM? Is the number of peaks in Clomics equivalent to a certain window size in VCMtools/PHM? How were the parameters picked for each method (e.g. the step size)? Could the authors elaborate on the choices made to more convincingly reassure that the comparisons across methods is fair with the chosen parameters?

These points align strongly with those mentioned in point 1 and we therefore kindly refer the reviewer to our answer above. Related to the point of the window sizes, we used the same window sizes in tests for VCMtools and PHM. If the reviewer meant to indicate the difference between VCMtools/PHM versus Clomics, we meant to use a similar variation in window sizes which was challenged by the fact that Clomics uses peak number instead of genomic windows. We now further elaborate on how we aimed to standardize this in the methods section (method section “Parameter selection for CM mapping”, lines 645-675).


3. What do red boxes represent in Figures S1.3 to S1.4? Are the red boxes highlighting the optimal conditions for the three methods compared? How were these chosen?

Indeed, the red boxes represent the parameter combinations which we considered to be optimal. This information is now included in the legends of Fig S1.3 and S1.4. Namely, we added a description for the red boxes to Fig S1.3 stating: “Across all panels, red rectangles indicate the optimal parameter combination that represents a compromise between the number of CMs versus the distribution, size and overlap with TADs and A/B compartments of these CMs”.

The parameter search we conducted revealed that the optimal parameters we suggest align well with the default settings for Clomics. However, we have made adjustments for VCMtools by reducing the window size to 1Mb (compared to 2Mb in the original study; Waszak et al., Cell, 2015), and for PHM by increasing the posterior probability cutoff to 0.8 (compared to 0.5 used in the original study with LCL ATAC-seq data; Kumasaka et al., Nature Genetics, 2019). Even though no ‘hard’ criteria were used to define the best parameter combinations, we believe that the indicated choices represent a good balance between the number of identified CMs, length and size distribution, and degree of overlap with TADs and A/B compartments. We now explicitly state this in the manuscript on line 154.


4. The integration of CM modules with 3D chromatin organization is very descriptive (starting on line 157), and could be strengthened by providing the enrichment over the expected values, to improve the quality of the analysis and help showcase that CMs are enriched in particular features of chromatin organization. For example, CMs overlap 25.7-31.9% with the B compartment and 59.4-63.9% with the A compartment. Is this overlap significantly different than expected, given the typical proportion of A and B compartments in the genome?

A compartments take up to 40% and B compartments 60% of the total genome length (e.g. Liu et al., Nature Communications, 2021). To formally address whether the observed percentages of CMs fully overlapping with A (59.4-63.9%) and B (25.7-31.9%) compartments deviate from the expected values, we have included a bootstrapping analysis. Specifically, we assessed the overlap of CMs with TADs, A/B compartments and controls, consisting of CMs shuffled along chromosomes (see Methods, lines 689-707). Mapped CMs showed higher overlap with TADs and A compartments compared to 95% confidence intervals obtained for bootstrapped CMs. This analysis is now described on lines 163-167: “The methods showed high concordance with respect to previously reported CM characteristics [17–21], such as CM length (median 8.6-31.7kb) and size (median 2-4 peaks) (Fig S1.5a), localization (59.4-63.9% CMs fully contained in A (active), compared to 30.5-33.3% for bootstrapped CMs; 25.7-31.9% CMs fully contained in B (inactive) compartments, compared to 50.0-53.9% for bootstrapped CMs; 68.1-73.2% CMs fully contained within TAD boundaries, compared to 44.5-49.3% for bootstrapped CMs (Fig S1.3d-f, indicated with red rectangles)), enrichment in 3D interactions between CM-embedded CREs (as inferred using either a Hi-C 500bp or Micro-C 500bp dataset; Fig S1.5d) and active chromatin states (Fig S1.5e-g) (Methods).”


5. According to the text, CMs found within TAD boundaries constitute 68.1-73.2%. Are these CMs confined within TAD boundaries or just overlapping by one single base? Statements like this one require more careful clarification.

We thank the reviewer for pointing this out. These values correspond to the CMs confined within TAD boundaries. We have clarified this in the manuscript (lines 163-167), methods (lines 691-694) and the legend of Fig S1.3.


6. In lines 158-160, why is 'n' so different between methods? Why is 'n' so high in Clomics compared to the other methods? How were the other final parameters determined?

This is related to the fact that Clomics has a higher power to detect more subtle correlations between peaks (CREs) due to a background-aware thresholding. We would like to direct the reviewer to lines 185-206 (and now also newly included lines 175-180) where we have discussed this in more detail.


7. In Figure 1c, the CMs are compared across the three methods, showing that Clomics identified around 30% more CMs. Is the ChIP-Seq signal distribution across high and low peak activity in the Clomics-only CMs similar to those CMs identified by all methods? In other words, is Clomics more sensitive at detecting CMs with certain types/quality of peaks? Likewise, the interactions in the SPIC examples seem to be much weaker and considerably less compared to interactions detected in the MB21D2 CM.

We have split the peaks into several groups: 1) peaks from CMs that were identified exclusively by one method (“method-specific peaks”), 2) peaks from CMs identified by more than one method (“peaks from CMs shared between methods”) and 3) peaks that were not identified as part of a CM (“not CM peaks”). Next, we calculated the average of the normalized peak counts and the standard deviation of peak signal intensity across individuals. Clomics-specific CM peaks showed largely comparable median statistics for peak heights and the standard deviation of peak heights across methods. In all cases, the method-specific peaks are at least equal in height to those captured by multiple methods, indicating that their detection is not related to power in terms of peak ‘height’. Peaks from Clomics-specific CMs did show somewhat of a broader range of average peak heights and their standard deviation across individuals compared to VCMtools- or PHM-specific CM peaks (now included in Fig S1.5i and discussed on lines 175-180). Together, this suggests that Clomics may have slightly higher power to detect covariable peaks across different peak height ranges compared to VCMtools or PHM, which is the case for the SPIC CM example in Fig 1c.


8. In Figure S1.6a, it is not clear what is plotted as ‘gene expression’? Are these values TPMs? FPKMs? Given the difference in the number of genes that overlap CMs vs those that do not, it would be a better metric to plot the median of expression.

We thank the reviewer for pointing this out. We had used CPM-normalized RNA-seq counts (now Fig S1.6c), as the coefficient of variation is best defined using positive count values. We have adapted the x-axis of the plot, and adjusted the associated figure legend and the Methods section (lines 714-730) to define this. In addition, we also noticed some redundancy in previous panels a and c, and therefore updated the figure by removing the panel a and adding the boxplots for median expression values for CM and not CM gene categories to the panel d. We would like to note that in Fig S1.6d, we used regressed out, variance stabilized and DESeq2 normalized RNA-seq data (see Methods, lines 591-594). This information is now added to the legend of Fig S1.6.


9. Is there a reason for using a ‘meta BAM’ file (lines 561-563) instead of taking peaks or regions that are common across different individual samples? Are the samples that were merged produced using the same technology?

We followed the strategy of creating a ‘meta BAM’ for calling a unified peak set, as this is more commonly done in population epigenomics studies (for example, Kumasaka et al., Nature Genetics, 2019; Delaneau et al., Science, 2019; Girdhar et al., Nature Neuroscience, 2022). All samples were sequenced on an Illumina Hiseq 2000.


10. In the section “Cell type specificity of interindividual variation” (starting on line 207), the authors have enhanced the details of gene expression data processing. However, the manuscript still lacks an explanation of how CMs are associated with genes. For instance, is each CM assigned to a single gene or multiple genes? Are they associated based on distance? Are any thresholds applied? It is essential to elaborate further and fully to enable reproducibility.

We would like to direct the reviewer to lines 714-730 (“CM overlap with A/B compartments, TADs, chromatin state annotations and genes” in the Methods section) where we described how CMs were associated to genes: “For every gene, we defined the promoter region as +-500bp from the TSS, and gene body as gene coordinates without promoter region. Gene coordinates (promoter start, gene body end) were overlapped with CM peaks with bedtools [95] intersect with the –wo to report the base pair overlap of CMs with the gene coordinates without restrictions on the overlap size (1bp was considered sufficient). If CM peaks overlapped more than one gene, the statistics were calculated for every CM-gene pair. To calculate the coefficient of variation (CV) of CM-embedded gene expression, we used counts per million (CPM)-normalized counts. To explore the differences between standard deviation, mean and median gene expression of genes embedded within CMs or genes outside of CMs, and all other aCM-gene correlation analyses, we used regressed out, variance stabilized and DESeq2 normalized RNA-seq data (see ChIP-seq, ATAC-seq and RNA-seq data processing). For the aCM/peak height to gene correlation analyses, we found the closest gene TSS to the entry of interest with bedtools [95] closest, and then proceeded with correlating either the aCM score or peak height with the closest gene expression. If several genes were equidistant, we considered all of them in the analyses.”.


11. In lines 217-219, the authors enumerate the cells they focus on but omit LCLs. Is it a typo or is there a more specific reason to exclude LCLs? It is important to explain in the manuscript.

We had mentioned before in the text that we mapped CMs for LCLs, and that is why it was omitted here. But we agree with the reviewer that this may confuse the reader, and have now adapted the sentence accordingly to “based on the preceding analyses, in this and following sections, we specifically focus on CMs mapped with Clomics in LCLs (n=18633), FIBs (n=10158), Monocytes (n=9002), Neutrophils (n=6604) and T cells (n=4841)”, lines 227-229.


12. It is very interesting to see that the number of reads does not influence the rank of the cells based on the number of CMs detected (Figure 2.1d) and that this could be due to the heterogeneity of the cells. The plot is however confusing, did all cell types have the same number of ‘all reads’? Important to clarify and improve the manuscript itself on this point.

We thank the reviewer for pointing this out, and the reviewer is correct that not every cell type had an equal total number of “all reads”. We have extended the figure panel S2.1d where we now indicate the average total number of reads per cell type. These numbers per sample and per cell type are outlined in Table S3, which we now also explicitly state on line 604.


13. Figure S2.1f-j and lines 229-233: the discrepancy between the figure labelling and the text can be confusing. The figure depicts the overlapping score whilst the text mentions the similarity score. Are these two terms equivalent? Sticking with one definition option would ease the understanding of the text. It is important to fix this problem.

These terms are indeed equivalent, and we apologize if this may have been confusing. We have adapted the text to match the description of the figure legend. The text now states: “This showed that the majority of CMs are cell type-specific while some cell types, such as Monocytes and Neutrophils, have a relatively higher proportion of overlapping CMs (Fig S2.1f-j).”, lines 241-243.


14. The examples shown in Figures 2b and S2.3a-c are very interesting. However, to support the more generalized claim made in lines 235-236, these correlations should be done genome-wide. Alternatively, the statement should be more cautiously phrased to acknowledge that the claim may apply only to specific examples.

To prevent overstating based on the examples, we have switched the two sentences around and the text now reads (lines 243-246): “Together, this demonstrates the prevalent lineage- and cell type-specificity of the regulatory landscapes captured in the form of CMs. Example loci for each category are shown in Fig 2b and Fig S2.2a, where cell type-specificity is also reflected in the association between CM activity and gene expression (Fig S2.3a-c).”


15. Details on how CREs are defined are missing (CM formation appears to be driven by functionally distinct groups of TFs’ starting from line 238). The authors only stated that the CRE annotations for LCLs were obtained from ChromHMM and SCREEN databases. How were CRE annotations from other cell types obtained? Did the CREs overlap gene promoters, exons, and introns or were exclusively extragenic? This could be important because, for instance, when comparing CMs in LCL with CMs in other cell types, the CREs identified as common anchors and that showed the highest activity, might represent gene promoters which are active in respective cell types. In contrast, CREs identified as non-anchors could represent potential enhancer regions, which in this case would explain their reduced level of activity compared to anchor CREs and their higher degree of cell-type specificity.

What we considered as ‘CREs’ throughout the manuscript are all peaks mapped using the H3K27ac and H3K4me1 signals, which in essence denote CREs. The common set of peaks we created is identical for all cell types, hence CREs (peaks) have the same coordinates across cell types yet vary in their chromatin mark enrichment, which, as hypothesized, is driven by cell type-specific gene regulatory programs. We have now added this information to the Methods section (lines 585-590) to ensure that it is clear how CREs are defined and that the regions are shared on coordinate but not on epigenome enrichment between the cell types.


16. In Figure S2.4 b legend, information on the statistical test applied is missing. Please add.

We have now added information on the Mann–Whitney U test that was used in the legend of Fig S2.4.


17. In Figure 2c, does “CT-restricted” stand for “cell-type-restricted”? Please add to the legend and text.

“CT-restricted” indeed stands for “cell type-restricted” and we have added this information to the legend of Fig 2.


18. The manuscript still lacks a clear explanation of how anchors and non-anchors are defined (line 267). Are anchors common CREs in at least one pairwise comparison, or do they need to be common to all cell types? This explanation should be clearly stated in the Methods section. A step-by-step schematic would aid in understanding the analysis and replicating it.

Common anchors are defined as TFs whose binding sites are found to be enriched at shared CM peaks (anchors) in all pairwise comparisons of cell types (yellow bar in Fig S2.4e). We have now included an additional description on the definition of anchors in the Methods section (lines 785-808), expanded the illustration in Fig S2.4 and the associated figure legend, which now states: “Schematic representation of the differential TFBS enrichment analysis strategy for CM peaks across all pairwise cell type comparisons. First, for a given cell type pair (in this example, we focused on the pair of “Monocytes and T cells”), for CM peaks from different cell types overlapping by at least 1bp, we split CM CREs into sets of anchor and non-anchor CREs. Next, we performed differential TFBS enrichment analysis with UniBind for 1) anchor vs non-anchor (union of cell type-specific) CREs, 2) non-anchor CREs in cell type 1 vs non-anchor CREs in cell type 2, and 3) non-anchor CREs in cell type 2 vs non-anchor CREs in cell type 1 (see Methods). For those TFs that passed the p-value threshold and pTPM cutoff, we obtained groups of TFs, defined based on enrichment at anchor and non-anchor CREs in cell type (CT)-restricted (TFBSs enriched in one or a few cell types) and CT-common manner (TFBSs enriched in all cell types).”


19. What is the expression status of the transcription factors identified in Figures 2 and S2.4? Are all these transcription factors expressed in the cell lines compared?

To address this question, we have now added transcription factor (TF) expression data from the Human Protein Atlas in Fig S2.5a-b, and Methods, lines 595-600. The heatmaps in Fig S2.5a show TF expression (in log10 (protein transcripts per million (pTPM) + 1) per category. This highlights that many of the TFs are expressed in most of the cell types, whereas in particular the cell type-restricted non anchors display the most variable expression between cell types and thus represent the strongest cell type-specific category. This is further underlined by the violin plots in Fig S2.5b, which show the coefficient of variation (CV) of TF expression (measured in pTPM) per category, where each point indicates a TF. The brackets with stars on top indicate significant differences in the CV of TF expression between the categories, whereas the absence thereof indicates a non-significant difference (p-value > 0.05, Mann-Whitney U test).


20. Figure S2.5a-d panels are not mentioned in the text and can therefore be removed.

We thank the reviewer for pointing this out. These panels were not mentioned in the manuscript, and in particular after inclusion of the new S2.5a-b, they no longer add to the manuscript and we have therefore removed these panels.


21. In Figure S2.5e, what does “p” in “pTPM” stand for?

pTPM stands for protein-transcripts per million. We have now included this information in the legend of Fig S2.5.


22. In Figures 3b and S3.1a-d, the plots in the left panels display the distribution of absolute values of the correlations between peak height and gene expression for peaks in different states. While the differences between the peaks in different states are significant for most of the analysis, the median values indicate a weak correlation between peak heights and expression. We believe that these results should be interpreted accordingly, also in the text.

We have adapted the text to include a statement that the correlations are not very strong. It now reads: “This revealed that CM CREs show higher (albeit modest) correlations with expression of the closest gene at larger genomic distances compared to non-CM-embedded CREs.”, lines 316-319.


23. Missing reference to results section in lines 305-306.

We thank the reviewer for spotting this. We have removed the sentence as it will be discussed in more detail in the section following this sentence.


24. In lines 305-306, the authors mention that "in all conditions, the CREs closest to TSS have the highest correlation to gene expression". This statement is partially incorrect because, according to Figures 3b and S3.1a-d, the differences in correlations between the closest CM peak and the closest non-CM peak are significant only for LCLs and Monocytes, and not for the other cell types.

The reviewer is correct in this assessment which aligns with point 23. We have removed the sentence and replaced it with: “This revealed that CM CREs show higher (albeit modest) correlations with expression of the closest gene at larger genomic distances compared to non-CM-embedded CREs” (lines 316-319).


25. In lines 311-313, it should be "significantly higher correlation" instead of "significantly stronger". As mentioned before, the median correlation values point to a weak correlation between CMs and gene expression. Are the CMs relevant for the study of gene expression?

We have adapted the “stronger” to “higher” in the indicated lines of the manuscript. In terms of using CMs for explaining gene expression: the individual peaks in CMs on themselves may not significantly enhance the number of genes for which the expression can be explained from the epigenome. As the reviewer also indicates, the correlation between CM peaks and non-CM peaks to gene expression is only marginally stronger. However, when we consider the compound activity of multiple CM peaks (which we capture as the aCM score in the manuscript), we infer many more significant associations with gene expression (at least 2-fold, outlined in lines 308-326). This shows that CMs provide a means to identify co-regulated peaks that together dictate expression of a nearby gene, and aligns with the observation that, frequently, multiple CREs together are responsible for modulating gene expression. These observations support the notion that CMs can contribute to advancing our understanding of gene regulation. Moreover, they align with similar concepts put forth by others (e.g., highly interconnected enhancer communities, microdomains, chromatin nanodomains, etc. (for more details, please see our review (van Mierlo et al., Trends in Genetics, 2023)), and underline the observation that promoter-enhancer and enhancer-enhancer communication is widespread across the genome and an essential mechanism underlying gene regulation. Our experimental studies of CMs (Llimos et al., Nature Communications, 2022; Kribelbauer et al. BioRxiv, 2023), validate these observations, and indicate that CMs allow to uncover such communication in a fashion that is both orthogonal and complementary to, for example, chromosome conformation capture-based methodologies. We therefore believe that CMs are relevant for studying gene expression. We also discuss this in the Discussion section, lines 533-552.


Minor points:


26. Check figure legends and text for consistency in explaining abbreviations; in some cases, abbreviations are only explained after their first occurrence.

We have carefully assessed the text and legends to ensure that all abbreviations are defined at their first occurrence.


27. Double-check for typos and inconsistencies in abbreviations. For example, sometimes figures are referred to as "Figure X," while other times they are referred to as "Fig. X." Additionally, supplemental labelling is missing (i.e., S in Fig. S2.4a in line 255).

We have carefully assessed the text and legends to ensure that no typos or inconsistencies remain (this includes the change Fig 2.4a > Fig S2.4a).

**Third round of review**

**Reviewer 2**

We are satisfied with the revised work, except for the following points:

Major point 4: We couldn't find the reference provided by the authors as an example. Please provide the DOI.
Major point 5: There is inconsistency between the text and figure. The text refers to TAD boundaries, while figures refer to TAD. Please correct.
Major point 18. Common anchors are TFs or peaks harboring TF binding sites? It is not clear from the response provided in the rebuttal letter.
Major point 19. It will ease the interpretation if the authors split the CT-restricted non anchors per cell type, or label them somehow (S2.5.a)
Major points 22 and 24: Report in the text the median values for the correlations.

**Authors’ response to reviewers**

Reviewer #2: We are satisfied with the revised work, except for the following points:


Major point 4: We couldn't find the reference provided by the authors as an example. Please provide the DOI.

We were referring to the following paper, https://doi.org/10.1038/s41467-021-22666-3.


Major point 5: There is inconsistency between the text and figure. The text refers to TAD boundaries, while figures refer to TAD. Please correct.

We adapted the text, so that it is consistent between the main text and figure legends. Namely, we decided to use TADs instead of TAD boundaries, since TADs are defined by their start and end coordinates. Now, we explicitly mention this in the methods section “CM overlap with A/B compartments, TADs, chromatin state annotations and genes”: “TAD and A/B compartment (start, end) coordinates (for GM12878, GSE63525) were overlapped with CM coordinates (CM start and end) using bedtools [93] function intersect with the –wo to report the base pair overlap of CMs with the regions of interest.”


Major point 18.. Common anchors are TFs or peaks harboring TF binding sites? It is not clear from the response provided in the rebuttal letter.

We define common anchors as TFs, namely, and as indicated in the response to pt.18 from our previous rebuttal: “Common anchors are defined as TFs whose binding sites are found to be enriched at shared CM peaks (anchors) in all pairwise comparisons of cell types (yellow bar in Fig S2.4e).”


Major point 19. It will ease the interpretation if the authors split the CT-restricted non anchors per cell type, or label them somehow (S2.5.a)

We have provided an additional heatmap for the CT-restricted, non anchors category in Fig S2.5a with indication of presence/absence of a TFBS being enriched in each cell type.


Major points 22 and 24. Report in the text the median values for the correlations.

Now we report the median values in the respective figure legends, namely in Fig 3b and Fig S3.1a-d.
